# Synthesis of 2‐Substituted Adenosine Triphosphate Derivatives and their use in Enzymatic Synthesis and Postsynthetic Labelling of RNA

**DOI:** 10.1002/cbic.202500241

**Published:** 2025-05-21

**Authors:** Ugnė Šinkevičiūtė, Tania Sanchez‐Quirante, Samanta Rožánková, Lenka Poštová Slavětínská, Veronika Raindlová, Michal Hocek

**Affiliations:** ^1^ Institute of Organic Chemistry and Biochemistry Czech Academy of Sciences Flemingovo nam. 2 CZ‐16610 Prague 6 Czech Republic; ^2^ Department of Organic Chemistry Faculty of Science Charles University in Prague Hlavova 8 CZ‐12843 Prague 2 Czech Republic

**Keywords:** click reactions, DNA polymerases, nucleosides triphosphates, nucleotides, polyA polymerase, RNA polymerases, thiol‐ene addition

## Abstract

A series of adenosine triphosphate (ATP) derivatives bearing chloro, fluoro, amino, methyl, vinyl, and ethynyl groups at position 2 are synthesized and tested as substrates for RNA and DNA polymerases. The modified nucleotides work well in in vitro transcription with T7 RNA polymerase and primer extension (PEX) using engineered DNA polymerases (TGK, 2M) except for the bulkier 2‐vinyl‐ and 2‐ethynyl‐ATP derivatives that give truncated products. However, in single nucleotide incorporation followed by PEX, they still can be used for site‐specific incorporation of reactive modifications into RNA that can be further used for postsynthetic labeling through thiol‐ene or Cu‐catalyzed alkyne‐azide cycloadditions reactions. All modified ATPs work in polyadenylation catalyzed by poly(A) polymerase to form long 3′‐polyA tails containing the modifications that also can be used for labeling.

## Graphical abstract

A series of 2‐substituted adenosine 5′‐O‐triphosphate derivatives is synthesized and tested as substrates for polymerases. In vitro transcription with T7 RNA polymerase, primer extension with engineered DNA polymerases, or 3′‐labelling with poly(A) polymerase are used for enzymatic synthesis of base‐modified RNA. Further postsynthetic reactions with 2‐vinyl‐ or 2‐ethynyladenosine are used to attach fluorescent labels.

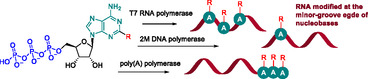

## 1 Introduction

RNA vaccines have shown great importance in combating SARS‐CoV‐2 pandemics,^[^ ^1^ ^]^ but in general, mRNA vaccines and other RNA therapeutics^[^ ^2^ ^]^ have great potential in treatment of many other diseases including cancer or neurodegenerations.^[^ ^3^ ^]^ Also, as natural eukaryotic mRNAs contain large portfolio of base modifications,^[^ ^4^ ^]^ which regulate their stability and translational efficacy, introduction of modified nucleobases into RNA is crucial for successful development of new RNA therapeutics. It has been demonstrated that mRNA vaccines need to contain modified bases, i.e., N1‐methylpseudouridine,^[^ ^5^ ^]^ 5‐methylcytosine,^[^ ^6^ ^]^ 5‐hydroxymethylcytosine,^[^ ^7^ ^]^ or 2,6‐diaminopurine,^[^ ^8^ ^]^ to decrease their immunogenicity and/or to alter formation and interactions of secondary structures. There is certainly a lot of space for studying other modified nucleobases in RNA and other therapeutic oligonucleotides, and hence, reliable methods of their syntheses are needed.

Longer RNAs are typically synthesized enzymatically^[^ ^9^ ^]^ using in vitro transcription (IVT) with T7 RNA polymerase (T7 RNAP) and ribonucleoside triphosphates (rNTPs) as building blocks. When using modified rNTPs in IVT, the modifications are incorporated uniformly into the whole RNA sequence^[^ ^10^ ^]^ or randomly in case of in cellulo metabolic labeling.^[^ ^11^ ^]^ Site‐specific modification of RNA is very challenging^[^ ^12^ ^]^ and it is used to be achieved mainly through ligation of shorter chemically synthesized fragments^[^ ^13^ ^]^ or by laborious and difficult to optimize position‐selective labeling by T7 RNAP^[^ ^14^ ^]^ based on omitting one nucleotide from IVT allows to pause synthesis and to incorporate modification at this position. Other approaches include posttranscriptional labeling through methyltransferases^[^ ^15^ ^]^ or ribozymes,^[^ ^16^ ^]^ or use of unnatural base‐pairs.^[^ ^17^ ^]^ Recently, we^[^ ^18^ ^]^ and others^[^ ^19^ ^]^ have developed conceptually different enzymatic synthesis of modified RNA through primer extension (PEX) using engineered DNA polymerases (i.e., TGK, SFM4‐3, and others). This approach enables to pause the synthesis, digest or exchange templates, and thereby, introduce site‐specific or segmental modifications. It has been demonstrated for number of sugar‐ and base‐modified nucleotides, and isotopically labeled nucleotides (for NMR). However, all the base‐modified rNTPs previously studied were 5‐substituted pyrimidine or 7‐substituted 7‐deazapurine nucleotides with modifications pointing out to the major‐groove and not interfering with Watson–Crick base‐pairing, and hence well tolerated by DNA polymerases. The modifications included fluorophores, affinity tags or reactive groups for posttranscriptional bioconjugations,^[^ ^20^ ^]^ or cross‐linking with proteins.^[^ ^21^ ^]^ In contrast, minor‐groove modifications are underexplored mainly because bulkier modifications would interfere with the base‐pairing, and hence, hamper the incorporation by polymerases. Nonetheless, some purine 2′‐deoxyribonucleoside triphosphates bearing diverse substituents at position 2 have been studied as substrates for polymerases and it was found that smaller groups, i.e., halogen, methyl, vinyl, ethynyl,^[^ ^22^, ^23^ ^]^ formyl,^[^ ^24^ ^]^ carboxy,^[^ ^25^ ^]^ or even allyl‐ and propargylamino,^[^ ^26^ ^]^ are tolerated by several DNA polymerases and the corresponding nucleotides are incorporated into DNA. The minor‐groove display of reactive vinyl, ethynyl, or formyl groups was used for conjugations through thiol‐ene, Cu‐catalyzed alkyne‐azide cycloadditions (CuAAC),^[^ ^22^, ^26^ ^]^ reductive aminations,^[^ ^24^ ^]^ or inverse‐electron demand Diels–Alder reactions.^[^ ^27^ ^]^ In ribonucleotides, besides above mentioned 2‐aminoadenine (base Z),^[^ ^8^ ^]^ only a handful of 2‐substituted purine rNTPs were prepared by enzymatic phosphorylations and used for 3′‐tail labeling of RNA with polyA polymerase.^[^ ^28^, ^29^ ^]^ Interestingly, also, 2‐ethynyladenosine nucleoside was used^[^ ^28^ ^]^ for in cellulo labeling of mRNA through polyadenylation. To complement and generalize the topic, here, we present a systematic study of the chemical synthesis of 2‐substituted adenosine triphosphate (ATP) derivatives and their substrate activities with T7 RNAP, engineered DNA polymerases, and polyA polymerase.

## 2 Results and Discussion

**Scheme 1:**
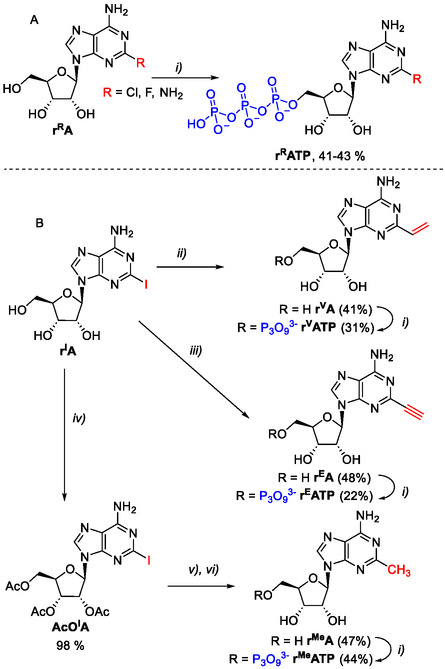
Synthesis of modified **r** ^**R**^ **NTP**s through A) triphosphorylation of commercial nucleosides or B) through cross‐coupling reactions at position 2. Reagents and conditions: i) 1. POCl_3_, PO(OMe)_3_, 0 °C, 3 h; 2. (Bu_3_NH)_2_H_2_P_2_O_7_, Bu_3_N, DMF, 0 °C, 1 h; 3. 1 m TEAB, 0–22 °C, 1 min; ii) potassium vinyl(trifluoro)borate, Cs_2_CO_3_, TPPTS, Pd(OAc)_2_, H_2_O/MeCN (2:1), 80 °C, 2.5 h; iii) 1. TMS‐acetylene, PdCl_2_(PPh_3_)_2_, CuI, Et_3_N, DMF, rt, 3 h; 2. K_2_CO_3_, MeOH, 22 °C, 1.5 h; iv) DMAP, TEA, Ac_2_O, MeCN, 22 °C, 1 h; v) Me_4_Sn, Pd(PPh_3_)_4_, NMP, 80 °C, 2 h; vi) K_2_CO_3_, MeOH, 22 °C, overnight.

For our study, a small series of six modified **r** ^**R**^ **ATP** were prepared with small modifications at position 2 (Cl, F, NH_2_, CH_3_, vinyl, and ethynyl). In the case of Cl, F, and NH_2_ modifications, commercially available nucleosides (**r** ^**Cl**^ **A**, **r** ^**F**^ **A**, **r** ^**NH**2^ **A**) were triphosphorylated^[^ ^30^ ^]^ to obtain desired triphosphates (**r** ^**Cl**^ **ATP**, **r** ^**F**^ **ATP**, **r** ^**NH**2^ **ATP**) in good 41%–43% yields (**Scheme** **1**A). In the case of Me, vinyl, and ethynyl, synthesis started with the commercially available 2‐iodoadenosine (**r** ^**I**^ **A**), followed by cross‐coupling reactions to introduce the C‐substituents at position 2. Thus, the Suzuki–Miyaura cross‐coupling reaction with vinyl(trifluoro)borate^[^ ^22^ ^]^ gave the 2‐vinyladenosine (**r** ^**V**^ **A**)^[^ ^31^ ^]^ in 41% yield. The Sonogashira cross‐coupling reaction with TMS‐acetylene followed by treatment with K_2_CO_3_ in methanol gave the 2‐ethynyladenosine (**r** ^**E**^ **A**)^[^ ^32^ ^]^ in 48% yield. For the introduction of a methyl group, the ribose was first protected by acetylation followed by the Stille coupling with tetramethylstanane and deprotection to give 2‐methyladenosine (**r** ^**Me**^ **A**).^[^ ^33^ ^]^ The 2‐substituted adenosines were then triphosphorylated by standard procedure^[^ ^30^ ^]^ to obtain final triphosphates (**r** ^**V**^ **ATP**, **r** ^**E**^ **ATP**, **r** ^**Me**^ **ATP**) in 22%–44% yield (Scheme 1B).

**Figure 1:**
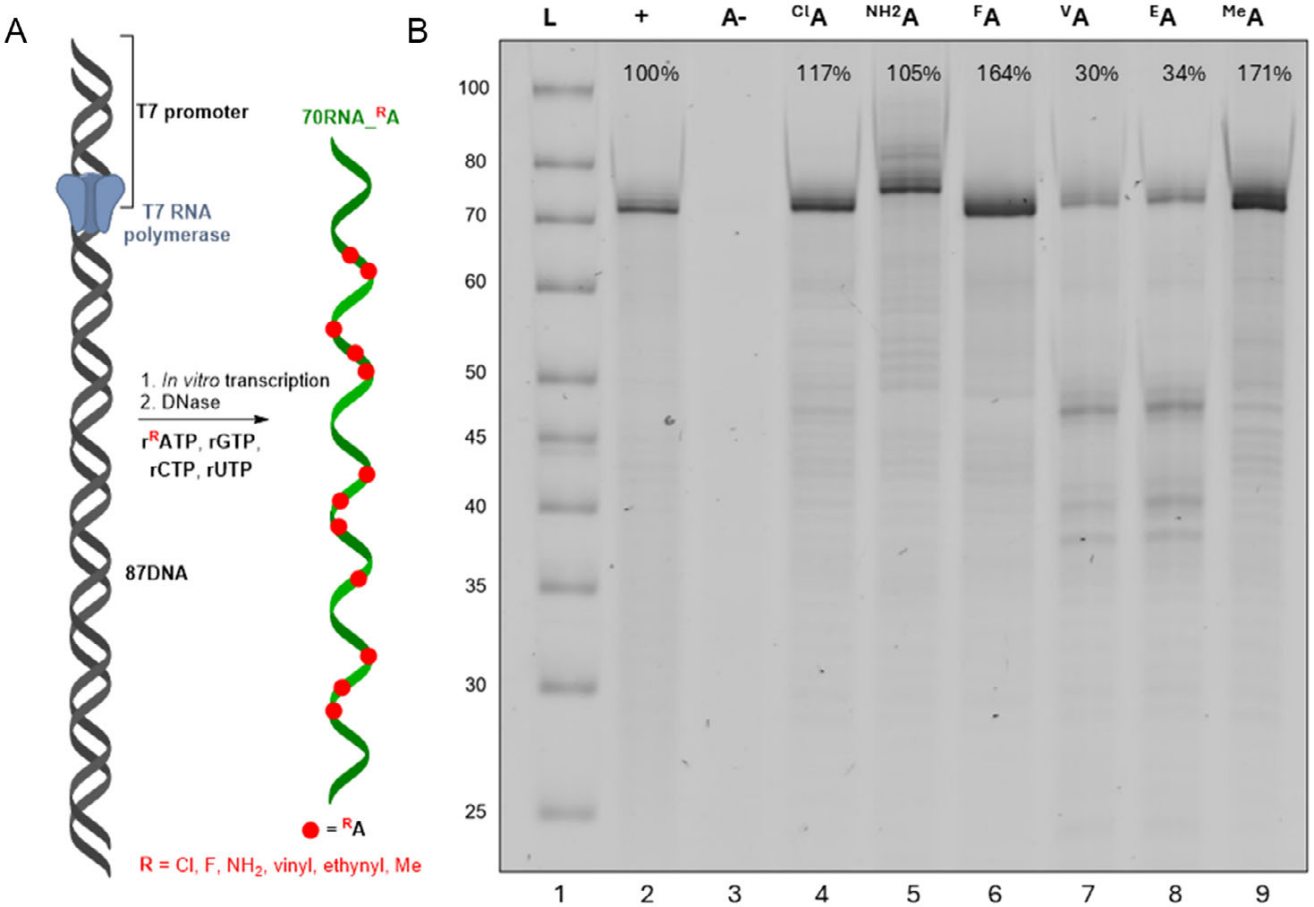
A) General scheme of IVT syntheses of modified RNA. B) 15% dPAGE of **70RNA_** ^**R**^ **A** products of IVT. Lane 1, **L ‐** ladder; Lane 2, **+**—positive control (four natural rNTPs); Lane 3, **A‐**—negative control (rGTP, rCTP, rUTP); Lane 4, ^**Cl**^ **A**–IVT with ^**Cl**^ **ATP**, rGTP, rCTP, rUTP; Lane 5, ^**NH**2^ **A**–IVT with **r** ^**NH**2^ **ATP**, rGTP, rCTP, rUTP; Lane 6, ^**F**^ **A**–IVT with **r** ^**F**^ **ATP**, rGTP, rCTP, rUTP); Lane 7, ^**V**^ **A**–IVT with **r** ^**V**^ **ATP**, rGTP, rCTP, rUTP; Lane 8, ^**E**^ **A**–IVT with **r** ^**E**^ **ATP**, rGTP, rCTP, rUTP; Lane 9, ^**Me**^ **A**–IVT with **r** ^**Me**^ **ATP**, rGTP, rCTP, rUTP. SYBR Gold staining.

With the series of modified **r** ^**R**^ **ATP** in hand, we explored their substrate activity with different polymerases. At first, we studied IVT with T7 RNAP using 87‐bp dsDNA template (**87DNA**) (**Figure** **1**, Table S1, Supporting Information) resulting in 70mer modified RNA products containing 12 modified ^**R**^ **A** nucleotides (**70RNA_** ^**R**^ **A**). The outcome was analyzed by denaturing polyacrylamide gel electrophoresis (dPAGE) (Figure 1, lanes 4–9). Although, all modifications were successfully incorporated giving the expected full‐length products **70RNA_** ^**R**^ **A** in yields comparable or higher than the IVT with natural rNTPs (Figure 1, lane 2), in the case of **r** ^**V**^ **ATP** and **r** ^**E**^ **ATP**, the incorporation was more problematic, resulting in a lower amounts of full‐length products (relative conversions 30%–34%) and formation of truncated side‐products (Figure 1, lanes 7, 8). All final modified RNAs were characterized and confirmed by UPLC‐MS analysis (Table S2, Supporting Information, Entry 1–7, copies of chromatograms and spectra Part 5 – Figure S12–S25, Supporting Information).

**Figure 2:**
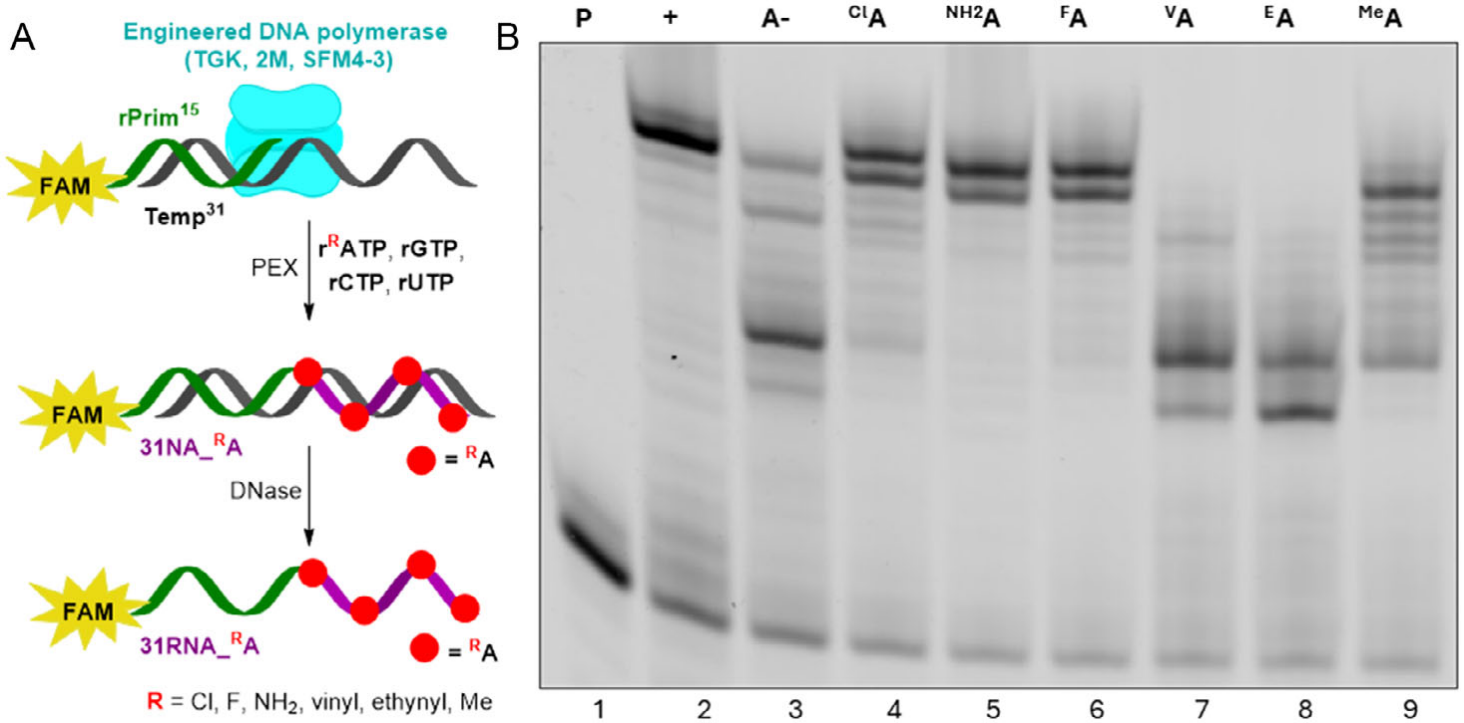
A) General scheme of PEX experiments and following RNAs generation. B) 15% dPAGE of 5′‐(6‐FAM)‐labeled **31NA_** ^**R**^ **A** after PEX reaction with TGK polymerase at 65 °C. Lane 1, **P** ‐ primer; Lane 2, **+**—positive control—PEX with four natural rNTPs; Lane 3, **A‐**—negative control—PEX with rGTP, rCTP, rUTP; Lane 4, ^**Cl**^ **A**–PEX with **r** ^**Cl**^ **ATP**, rGTP, rCTP, rUTP; Lane 5, ^**NH**2^ **A**–PEX with **r** ^**NH**2^ **ATP**, rGTP, rCTP, rUTP; Lane 6, ^**F**^ **A**–PEX with **r** ^**F**^ **ATP**, rGTP, rCTP, rUTP; Lane 7, ^**V**^ **A**–PEX with **r** ^**V**^ **ATP**, rGTP, rCTP, rUTP; Lane 8, ^**E**^ **A**–PEX with **r** ^**E**^ **ATP**, rGTP, rCTP, rUTP; and Lane 9, ^**Me**^ **A**–PEX with **r** ^**Me**^ **ATP**, rGTP, rCTP, rUTP.

Next, we tested the modified **r** ^**R**^ **ATP**s as substrates with engineered DNA polymerases (TGK,^[^ ^34^, ^35^ ^]^ 2M,^[^ ^36^ ^]^ or SFM4‐3^[^ ^37^ ^]^), that have been previously shown to be able to synthesize RNA or xenonucleic acids using PEX and single‐stranded DNA templates.^[^ ^18^, ^19^, ^34^, ^35^, ^36^, ^37^ ^]^ The PEX experiments were performed using a 15nt RNA primer (**rPrimer** ^**15**^) labeled at 5′‐end with 6‐carboxyfluorescein (6‐FAM) and 31nt long DNA template (**Temp** ^**31**^) with four possible sites for **r** ^**R**^ **ATP** incorporation (**Figure** **2**A, for sequences, see Table S1, Supporting Information). TGK (Figure 2B, Figure S2, Supporting Information) and 2M (Figure S3, Supporting Information) polymerases worked better than SFM4‐3 (Figure S4, Supporting Information) giving cleaner full‐length PEX products and less truncated byproducts with 2‐amino‐, 2‐halo‐, and 2‐methyladenine modifications. The resulting PEX products **31NA_** ^**R**^ **A**, (DNA–RNA hybrids) were analyzed by denaturing PAGE analysis (Figure 2B) and by UPLC‐MS after treatment with DNase. In case of **rA** ^**Cl**^ **TP**, **rA** ^**NH2**^ **TP,** and **rA** ^**F**^ **TP,** we observed formation of the expected full‐length **31RNA_** ^**R**^ **A** accompanied by *n* + 1 products formed by nontemplated addition of one rAMP or rGMP, while in case of **rA** ^**Me**^ **TP**, the full‐length product was just a minor component and the n − 1 truncated RNA was the main product (SI, Table S3, Supporting Information, Entry 2–5, copies of chromatograms and spectra Part 5 –Figure S30–S37, Supporting Information). Unfortunately, no full‐length products were obtained with **rA** ^**V**^ **TP** and **rA** ^**E**^ **TP** (Figure 2B, lanes 7–8 and Figure S2–S4, Supporting Information, lanes 7–8) even after optimization of concentrations or temperature.

**Figure 3:**
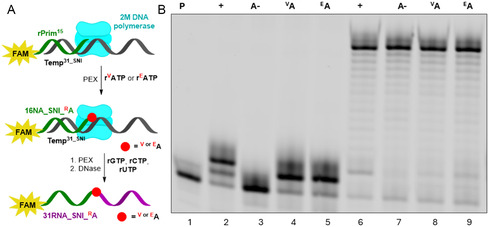
A) General scheme of SNI experiments with following RNAs generation. B) 15% dPAGE of 5′‐(6‐FAM)‐labeled **16NA_** ^**R**^ **A** and **31NA_** ^**R**^ **A** after SNI and PEX reactions with 2M polymerase. Lane 1, **P ‐** primer; Lane 2, **+**—positive control—SNI with natural rATP; Lane 3, **A‐**—negative control—water instead of any rNTP; Lane 4, ^**V**^ **A**–SNI with **r** ^**V**^ **ATP**; Lane 5, ^**E**^ **A**–SNI with **r** ^**E**^ **ATP**; and Lanes 6–9, PEX reactions of SNI products (from Lanes 2–5) with addition of rGTP, rCTP, and rUTP.

**Figure 4:**
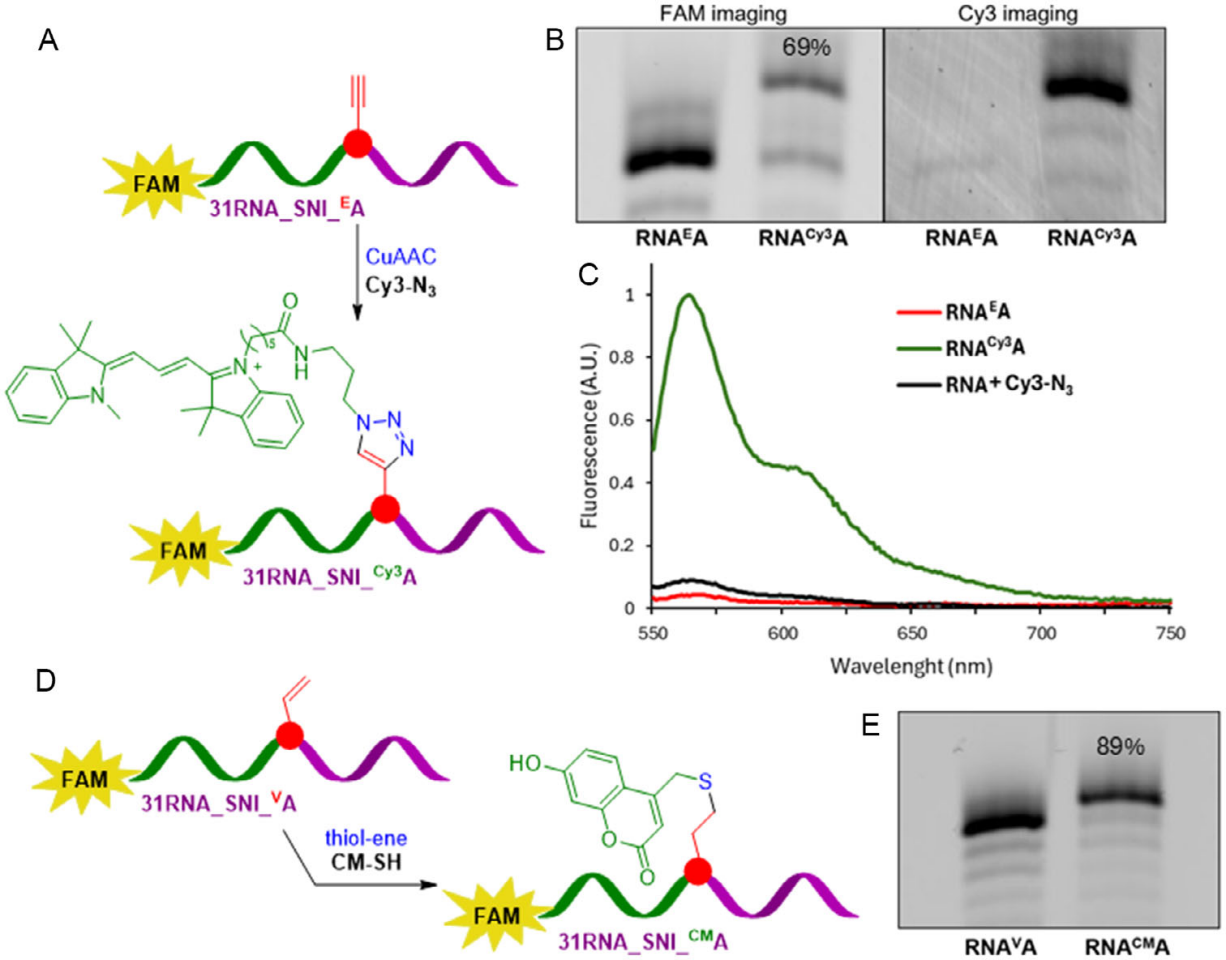
A) Schematic representation of postsynthetic modification reaction with Cy3–N_3_. B) 15% dPAGE analysis of SNI PEX product **31RNA_SNI_** ^**E**^ **A** and the product of subsequent CuAAC reaction **31RNA_SNI_** ^**Cy**3^ **A** (FAM and Cy3 scan), conversion 69%. C) Normalized emission spectra of **31RNA_SNI_** ^**Cy**3^ **A** compared to **31RNA_SNI_** ^**E**^ **A** before postsynthetic modification and after negative control reaction of nonmodified RNA with Cy3–N_3_. D) Schematic representation of postsynthetic modification reaction with CM–SH. E) 20% dPAGE analysis of SNI PEX product **31RNA_SNI_** ^**V**^ **A** and the product of subsequent thiol‐ene reaction **31RNA_SNI_** ^**CM**^ **A**, conversion 89%. Uncropped gels are given in Supporting Information, Part 3.

Although the PEX experiments with several incorporations of **r** ^**V**^ **ATP** and **r** ^**E**^ **ATP** did not lead to full‐length products, we decided to try single nucleotide incorporation (SNI) experiments followed by PEX with natural rNTPs. This approach has been previously used^[^ ^26^, ^38^, ^39^, ^40^ ^]^ for site‐specific incorporation of modifications into DNA and worked even with relatively bad substrates. The SNI experiments were set up with 6‐FAM‐labeled **rPrim** ^**15**^ and a new 31‐mer DNA template (**Temp** ^**31_SNI**^) (Table S1, Supporting Information) that was designed in a way that the modified **r** ^**R**^ **A** nucleotide would be incorporated first and the next nucleobase is different to prevent further extension of the primer in the absence of the complementary rNTP (**Figure** **3**A, for sequence see Table S1, Supporting Information). The analytical scale experiment was performed in 21 pmol while the semipreparative one in 84 nmol scale. The first step was the SNI using stoichiometric amounts of either **r** ^**V**^ **ATP** or **r** ^**E**^ **ATP** in the absence of any other rNTPs. In both cases, we observed successful incorporation of the modified nucleotide to obtain 16nt products (**16NA_SNI_** ^**V**^ **A** and **16NA_SNI_** ^**E**^ **A**) (Figure 3B, lane 4, 5). Then, the experiments continued by adding excess of the mixture of remaining three rNTPs (rGTP, rCTP, rUTP) and observed successful PEX to get clean full‐length products in nearly quantitative conversions (Figure 3B, lanes 8, 9). Both TGK (Figure S6, Supporting Information) and 2M (Figure 3B) DNA polymerases worked, but the 2M showed somewhat better efficacy in the extension with natural rNTPs to full‐length product (**31NA_SNI_** ^**V**^ **A** and **31NA_SNI_** ^**E**^ **A**) (Figure 3B, lane 8, 9). It should be noted that negative control experiment with mixture of rGTP, rCTP, and rUTP (lane 7) also gave full‐length product apparently due to one rGMP nucleotide misincorporation during the PEX (confirmed by UPLC‐MS – Figure S54–S55, Supporting Information [for 2M], Figure S58–S59, Supporting Information [for TGK], in SI). After the treatment with DNase, the identity of the modified RNA products **16RNA_SNI_** ^**R**^ **A** and **31RNA_SNI_** ^**R**^ **A** was confirmed by UPLC‐MS (Table S3, Supporting Information, Entry 6–9, copies of chromatograms and spectra Part 5 – Figure S38–S45, Supporting Information) The abovementioned experiments have shown that even though the vinyl and ethynyl modification in **rA** ^**V**^ **TP** and **rA** ^**E**^ **TP** might be sterically too bulky for multiple incorporation, the nucleotides can still be used for SNI followed by PEX, and hence, for site‐specific modification of RNA with clickable reactive groups suitable for further modification. Therefore, we further studied reactivity of **31RNA_SNI_** ^**V**^ **A** and **31RNA_SNI_** ^**E**^ **A** (**Figure** **4**). Similarly to our previous works,^[^ ^22^, ^26^ ^]^ we tested thiol‐ene reactions^[^ ^41^ ^]^ of the 2‐vinyladenine and CuAAC reactions^[^ ^42^, ^43^ ^]^ of the 2‐ethynyladenine in RNA with coumarine–methylthiol (**CM–SH**) or azide‐conjugated Cy3 (**Cy3–N**_**3**_) (Figure S7, Supporting Information). The CuAAC reaction of **31RNA_SNI_** ^**E**^ **A** with **Cy3–N**_**3**_ was performed in presence of CuBr, (tris(benzyltriazolylmethyl)amine) (TBTA), and sodium ascorbate in H_2_O/DMSO/tBuOH mixture overnight at 37 °C (Figure 4A). The dPAGE analysis showed good conversion of the click reaction and the product **31RNA_SNI_** ^**Cy3**^ **A** was visible both in FAM and Cy3 channels (Figure 4B, and Figure S8, Supporting Information). Also, the fluorescence emission spectroscopy (Figure 4C) and UPLC‐MS analysis confirmed covalent attachment of the Cy3 fluorophore. The thiol‐ene addition reaction of **31RNA_SNI_** ^**V**^ **A** with **CM–SH** was performed in 0.5 m TEAA buffer (pH 7) for 3 days at 37 °C (Figure 4D) and dPAGE analysis showed quantitative conversion (Figure 4E, and Figure S9, Supporting Information). In this case, the fluorescence of the coumarine was overlapped with the emission of the FAM label (data not shown) but the identity of the product **31RNA_SNI_** ^**CM**^ **A** was confirmed by dPAGE and UPLC‐MS analysis (Table S3, Supporting Information, Entry 10–11, copies of chromatograms and spectra Part 5 – Figure S46–S49).

**Figure 5:**
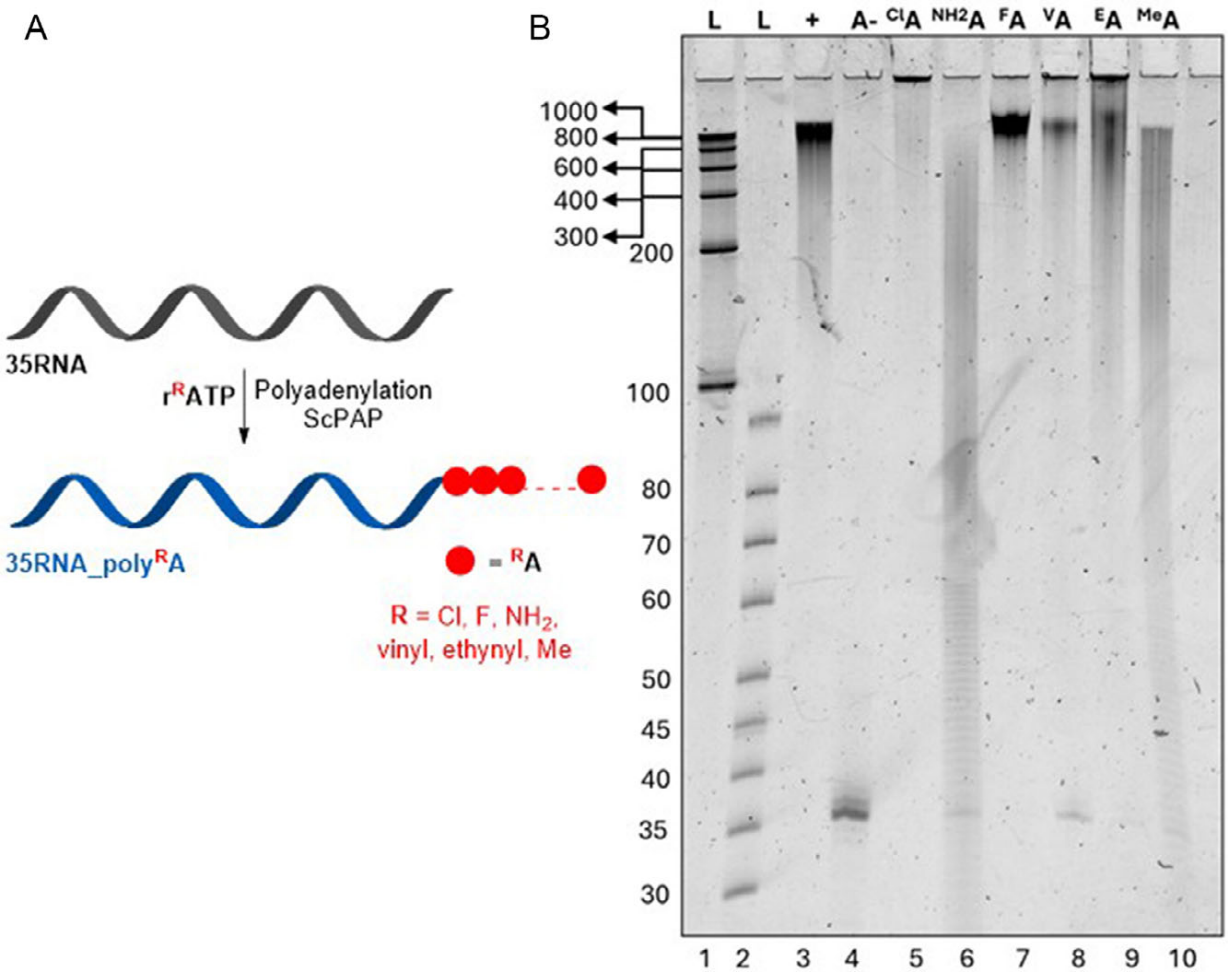
A) General scheme of polyadenylation experiments. B) 10% dPAGE of RNA products after polyadenylation reaction. Lanes 1,2, **L** ‐ ladder; Lane 3, **+**—positive control (natural **rATP**); Lane 4, **A‐**—negative control (water instead of natural or modified rATP); Lane 5, ^**Cl**^ **A**–**r** ^**Cl**^ **ATP** incorporation; Lane 6, ^**NH**2^ **A**–**r** ^**NH**2^ **ATP** incorporation; Lane 7, ^**F**^ **A**–**r** ^**F**^ **ATP** incorporation; Lane 8, ^**V**^ **A**–**r** ^**V**^ **ATP** incorporation; Lane 9, ^**E**^ **A**–**r** ^**E**^ **ATP** incorporation; and Lane 10, ^**Me**^ **A**–**r** ^**Me**^ **ATP** incorporation. SYBR Gold staining.

Inspired by recent work of Mitton‐Fry et al.^[^ ^28^ ^]^ where modified **r** ^**Cl**^ **ATP**, **r** ^**F**^ **ATP**, and **r** ^**NH**2^ **ATP** were successfully applied in polyadenylation of at 3′ end of RNA by yeast poly(A) polymerase (*Saccharomyces cerevisiae* (ScPAP)) to form long poly(A) tails containing modified adenosines, we wanted to test if our other three synthesized nucleotides: **r** ^**V**^ **ATP**, **r** ^**E**^ **ATP**, and **r** ^**Me**^ **ATP** could be used with poly(A) polymerase to form hypermodified poly(A) tails. The experiments were conducted with 35nt RNA template (**35RNA**) which was synthesized via IVT (**Figure** **5**A) (IVT details in Table S1, Supporting Information, Part 2.2.3, Table S2, Supporting Information ‐ Entry 8, Part 2.5, and Part 5, Figure S26,S27, Supporting Information). All the tested modified nucleotides were successfully incorporated forming long hypermodified poly(A) tails (Figure 5B). To our surprise, even **r** ^**V**^ **A** and **r** ^**E**^ **A** modifications were incorporated resulting in products longer than 1000 nt (Figure 5B, lane 8,9), while the use of **r** ^**Me**^ **ATP** gave shorter product of ≈1000 nt (Figure 5B, lane 10).

**Figure 6:**
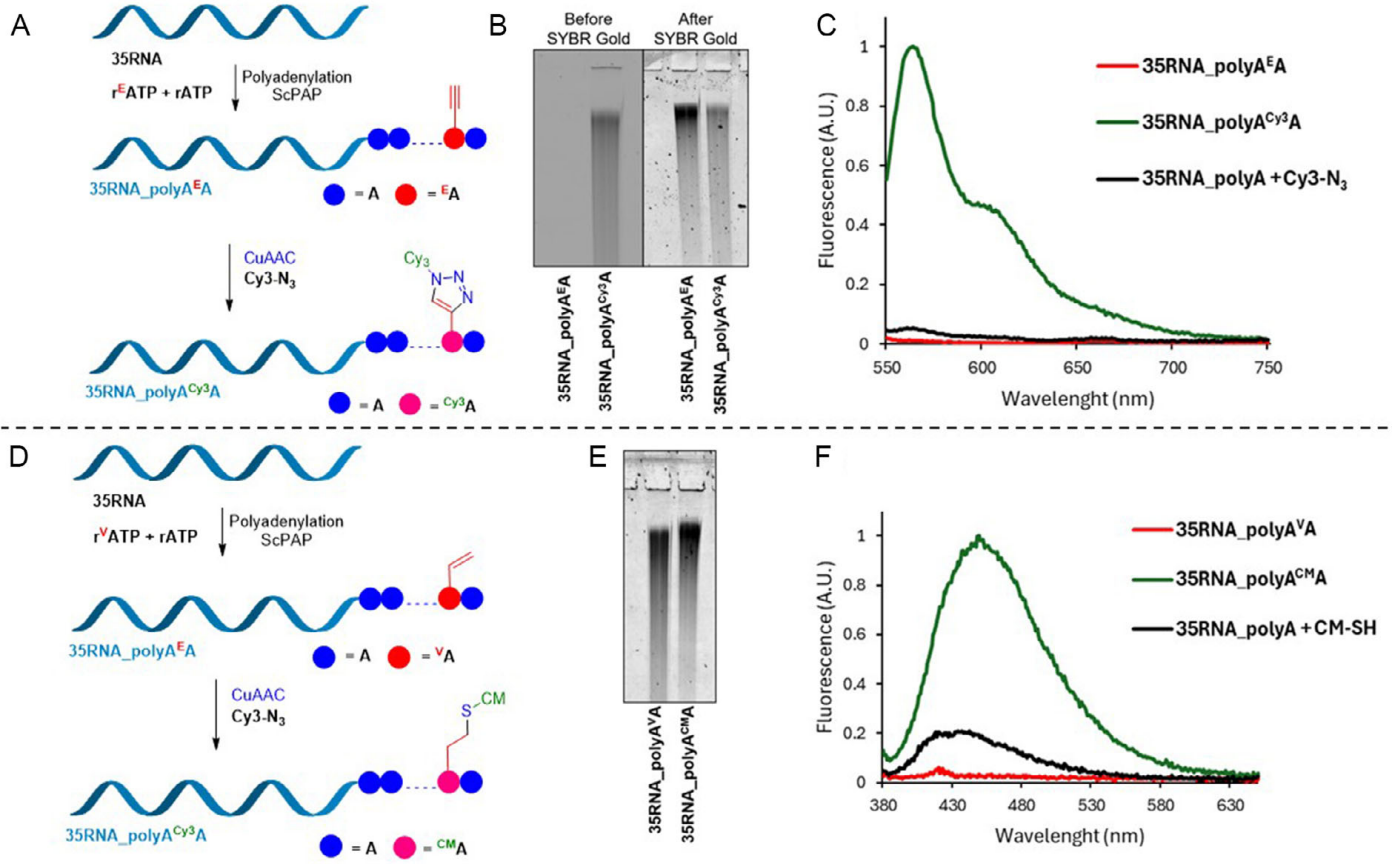
A) Schematic representation of postsynthetic modification reaction with Cy3–N_3_. B) 15% dPAGE analysis of polyadenylation reaction product **35RNA_polyA** ^**E**^ **A** and the product of subsequent CuAAC reaction **35RNA_polyA** ^**Cy**3^ **A** (Cy3 scan before and after SYBR Gold staining). C) Normalized emission spectra of **35RNA_polyA** ^**Cy**3^ **A** compared to **35RNA_polyA** ^**E**^ **A** before postsynthetic modification and nonmodified **35RNA_polyA** after negative control reaction with **Cy3–N**_**3**_. D) Schematic representation of postsynthetic modification reaction with **CM–SH**. E) 15% dPAGE analysis of polyadenylation reaction product **35RNA_polyA** ^**V**^ **A** and the product of subsequent thiol‐ene reaction **35RNA_polyA** ^**CM**^ **A** (Cy3 scan after SYBR Gold staining). F) Normalized emission spectra of **35RNA_polyA** ^**CM**^ **A** compared to **35RNA_polyA** ^**V**^ **A** before postsynthetic modification and nonmodified **35RNA_polyA** after negative control reaction with **CM–SH**. Uncropped gels can be seen in Supporting Information, Part 3.

Finally, we tested if we can use modified **rA** ^**V**^ **ATP** and **r** ^**E**^ **ATP** in mixture with excess of natural rATP to form poly(A) tails with random incorporation of vinyl or ethynyl modifications for postsynthetic labeling. The polyadenylation experiments were performed using a 9:1 ratio of rATP versus **r** ^**V**^ **ATP** or **r** ^**E**^ **ATP** followed by the CuAAC or thiol‐ene click reactions (**Figure** **6**, details in Supporting Information, Part 2.6). In both cases, the poly(A) tails were successfully formed and the follow up reactions of **35RNA_polyA** ^**E**^ **A** with **Cy3‐N**_**3**_ (Figure 6A) and of **35RNA_polyA** ^**V**^ **A** with **CM–SH** (Figure 6D) gave labeled products that were verified by dPAGE analyzes (Figure 6B,E, and Figure S10,S11, Supporting Information), where the **35RNA_polyA** ^**Cy3**^ **A** product was also visible without SYBR Gold staining in the Cy3 channel (Figure 6B). In both cases, the emission spectra (Figure 6C,F) confirmed the successful attachment of the Cy3 or CM fluorophores.

In conclusion, we have synthesized a small series of 2‐substituted rATP derivatives and tested them as substrates for T7 RNAP (in IVT), for TGK and 2M DNA polymerases (in PEX) and ScPAP (in polyadenylation reaction). The 2‐halo‐, 2‐amino‐, and 2‐methyl rATP derivatives were good substrates for all these polymerases (comparable or better than natural rATP) and worked in all the experiments. In contrast, the bulkier 2‐vinyl‐ and 2‐ethynyl nucleotides **rA** ^**V**^ **ATP** and **r** ^**E**^ **ATP** were significantly worse substrates with T7 RNAP giving only partial formation of full‐length products in IVT and even worse with TGK or 2M, where no formation of full‐length products was observed in PEX. In contrast, both **r** ^**V**^ **ATP** and **r** ^**E**^ **ATP** were successfully used for single nucleotide extension followed by PEX to synthesize site‐specifically modified RNA bearing vinyl or ethynyl group in the minor‐groove edge suitable for postsynthetic labeling though thiol‐ene or CuAAC click reactions. All tested **r** ^**R**^ **ATP** derivatives were successfully used in polyadenylation of RNA using ScPAP to form long 3′‐poly(A) tails either containing only modified nucleotides or random distribution of modifications and nonmodified adenosines. In the latter case, the postsynthetic click reactions were also used for the labeling of RNA tails with fluorophores. Apparently, the bulkier vinyl and ethynyl groups can partly interfere with the Watson–Crick base‐pairing in the active site of the template‐dependent polymerases (T7 RNAP or TGK or 2M DNAP) but have less negative effect in the template‐independent ScPAP. The presented approach can be used for minor‐groove edge labeling of RNA.

## Conflict of Interest

The authors declare no conflict of interest.

## Supporting information

Supplementary Material

## Data Availability

The data that support the findings of this study are available in the supplementary material of this article and all raw data are deposited at: https://doi.org/10.48700/datst.qgz5g‐hca80.
