# Supplementary material for: Synthesis of 2‐Substituted Adenosine Triphosphate Derivatives and their use in Enzymatic Synthesis and Postsynthetic Labelling of RNA
Source: Chembiochem. 2025 May 21;26(12):e202500241. doi: 10.1002/cbic.202500241 (PMC12177693; doi:10.1002/cbic.202500241)
Supplement: Supplementary file 1 — Supplementary Material [file CBIC-26-e202500241-s001.pdf]

Supporting information

1. Chemical synthesis 2
2. Enzymatic synthesis 10
General remarks 10
2.1 List of oligonucleotides used in this study 11
2.2 *In vitro* transcription 12
2.2.1 Preparation of double-stranded DNA templates 12
2.2.2 Synthesis of 70mer RNA 12
2.2.3 Synthesis of 35mer RNA 13
2.3 Primer extension experiments (PEX) 13
2.3.1 Multiple incorporations of modified r^{R}ATPs 13
2.3.2 Single nucleotide incorporation (SNI) experiments 16
2.3.3 Semi-preparative preparation of modified RNA 18
2.4 Post-synthetic modifications of modified RNA 19
2.4.1 Click reaction of 31RNA_SNI_^{E}A 19
2.4.2 Thiol-ene addition reaction of 31RNA_SNI_^{V}A 20
2.5 Polyadenylation experiments 20
2.5.1 Semi-preparative preparation of modified 35RNA_polyA^{E}A and 35RNA_polyA^{V}A 21
2.6 Post-synthetic modifications of modified poly(A) 21
2.6.1 Click reaction of 35RNA_polyA^{E}A 21
2.6.2 Thiol-ene addition reaction of 35RNA_polyA^{V}A 22
3. Uncropped gels 23
4. Copies of NMR spectra 27
5. Copies of UPLC-MS chromatograms and spectra 35
6. Analysis of positive and negative controls in SNI experiments 59

7. References 61

Experimental part

1. Chemical synthesis

General remarks

All chemicals were purchased of analytical grade from commercial suppliers and used as received (Fluorochem, Sigma Aldrich, Jena Biosciences). Phosphoryl chloride ($POCl_3$) and trimethyl phosphate [$PO(OMe)_3$] were distilled prior to use. The course of reactions was followed by silica gel thin-layer chromatography (TLC) in silica gel 60 F_{254} (Merck Life Sciences) plates with UV light detection (254 and 365 nm), in the case of triphosphates IPAV (isopropyl alcohol /NH_4OH /H_2O, ratio 11/7/2) was used. The masses of individual spots on TLC plate were measured by Advion Expression Compact Mass Spectrometer connected with Plate Express® TLC Plate Reader (TLC-MS) using electrospray ionization (ESI). Purification of the ribonucleosides was carried out on CombiFlash Rf+ with columns filled with Silica gel on a CombiFlash Teledyne ISCO system. Purification of the ribonucleoside triphosphates was performed using HPLC (Waters modular HPLC system) on a SepharoseQ (VWR International or Merck Life Sciences) column and Phenomenex Kinetex 5 µm EVO C18 100 Å. NMR spectra were measured on a Bruker AVANCE 500 NMR spectrometer (^{1}H at 500.0 MHz, ^{13}C at 125.7 MHz and ^{31}P at 202.3 MHz) in hexadeuteriodimethylsufoxide (DMSO-d6) or deuterium oxide (D_2O) referenced to the residual solvent signal. Chemical shifts are given in ppm (δ scale), coupling constants (*J*) in Hz. Low resolution mass spectra were measured on LCQ Fleet (Thermo Fisher Scientific) using electrospray ionization (ESI). High resolution mass spectra were measured on LTQ Orbitrap XL (Thermo Fisher Scientific). All mass spectra were acquired by the MS service at IOCB. Starting ribonucleosides (2-chloroadenosine, 2-fluoroadenosine, 2-aminoadenosine, 2-iodoadenosine) were purchased from Fluorochem, 2-vinyladenosine, 2-ethynyladenosine and 2-methyladenosine were synthesized according to previously reported procedures. [1,2]

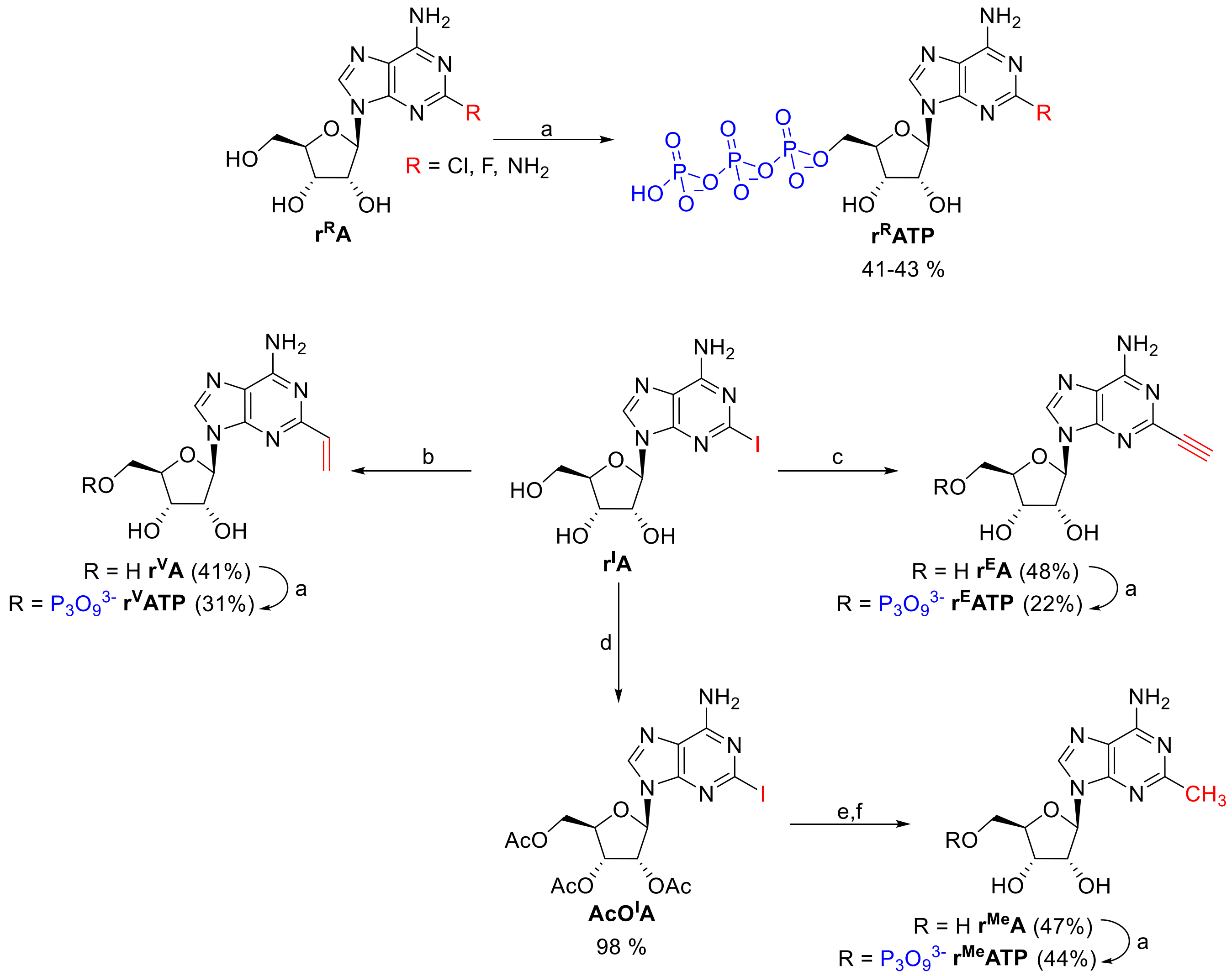

Scheme S1. a) 1. $POCl_3$, $PO(OMe)_3$, 0 °C, 3 h; 2. $(Bu_3NH)_2H_2P_2O_7$, Bu_3N, DMF, 0 °C, 1 h; 3. 1 M TEAB, 0–22 °C, 1 min; b) potassium vinyl(trifluoro)borate, Cs_2CO_3, TPPTS, $Pd(OAc)_2$, H_2O/MeCN (2:1), 80 °C, 2.5 h; c) 1. TMS-acetylene, $PdCl_2(PPh_3)_2$, CuI, Et_3N, DMF, rt, 3 h; 2. K_2CO_3, MeOH, 22 °C, 1.5 h; d) DMAP, TEA, Ac_2O, MeCN, 22 °C, 1 h; e) Me_4Sn, $Pd(PPh_3)_4$, NMP, 80 °C, 2 h; f) K_2CO_3, MeOH, 22 °C, overnight.

General procedure A: Starting nucleoside (1 equiv.) was dried overnight in vacuo. It was dissolved in dry $PO(OMe)_3$ and cooled down to 0 °C, then freshly distilled $POCl_3$ (1.2 equiv.) was added dropwise, and reaction mixture was stirred at the same temperature for 3h. After that, ice-cold solution of $(Bu_3NH)_2H_2P_2O_7$ (5 equiv.) and n-Bu_3N (4 equiv.) in dry MeCN was added dropwise and let to stir for another 1 h at 0 °C. After the reaction was complete it was treated with 2M TEAB (triethylammonium bicarbonate), concentrated, co-evaporated with distilled water (3x). The crude product was purified by two semi-preparative HPLC purifications – first by using SepharoseQ column (H_2O/800 mM TEAB in H_2O 1:0 → 0:1) and second purification with Kinetex EVO (0.1 M TEAB in H_2O/0.1 M TEAB in H_2O/MeOH 1:9 → 0:1).[3]

2-Chloroadenosine-5'-*O*-triphosphate (r^{Cl}ATP), triethylammonium salt

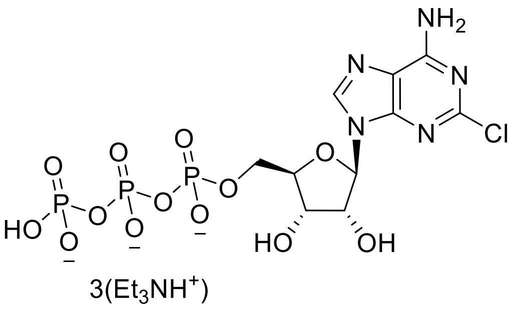

2-Chloroadenosine-5'-*O*-triphosphate (**r^{Cl}ATP**) was synthesized according to a **general procedure A** from 2-chloroadenosine (50 mg, 0.17 mmol) to obtain white solid (57 mg, 41%). ^{1}H NMR (600.1 MHz, D_2O): 4.22 (ddd, 1H, J_{gem} = 11.7 Hz, $J_{5'a,P}$ = 4.5 Hz, $J_{5'a,4'}$ = 2.9 Hz, H-5'a); 4.28 (ddd, 1H, J_{gem} = 11.7 Hz, $J_{5'b,P}$ = 6.1 Hz, $J_{5'b,4'}$ = 2.9 Hz, H-5'b); 4.40 (pent, 1H, $J_{4',5'a}$ = $J_{4',5'b}$ = $J_{4',3'}$ = $J_{4',P}$ = 2.9 Hz, H-4'); 4.59 (dd, 1H, $J_{3',2'}$ = 5.1 Hz, $J_{3',4'}$ = 3.4 Hz, H-3'); 4.78 (bt, 1H, $J_{2',1'}$ = $J_{2',3'}$ = 5.5 Hz, H-2'); 6.05 (d, 1H, $J_{1',2'}$ = 5.9 Hz, H-1'); 8.50 (s, 1H, H-8); ^{13}C NMR (150.9 MHz, D_2O): 67.22 (d, $J_{C,P}$ = 5.5 Hz, CH_2-5'); 72.39 (CH-3'); 76.34 (CH-2'); 86.14 (d, $J_{C,P}$ = 9.0 Hz, CH-4'); 88.71 (CH-1'); 119.57 (C-5); 142.05 (CH-8); 152.32 (C-4); 155.86 and 158.31 (C-2,6); ^{31}P NMR (202.4 MHz, D_2O): –22.60 (t, 1P, $J_{\beta,\alpha}$ = $J_{\beta,\gamma}$ = 19.9 Hz, P_β); –10.78 (d, 1P, $J_{\alpha,\beta}$ = 20.3 Hz, P_α); –10.09 (d, 1P, $J_{\gamma,\beta}$ = 19.6 Hz, P_γ). HR-ESI-MS: *found*: 539.9490 ([M + H]$^+$, calcd for $C_{10}H_{14}O_{13}N_5ClP_3^-$: 539.9495).

2-Aminoadenosine-5'-*O*-triphosphate ($r^{NH2}ATP$), triethylammonium salt

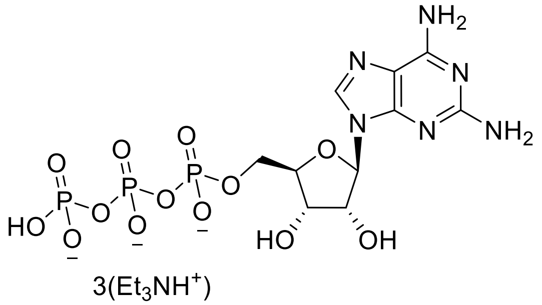

2-Aminoadenosine-5'-*O*-triphosphate (**r^{NH2}ATP**) was synthesized according to a **general procedure A** from 2-aminoadenosine (50 mg, 0.17 mmol) to obtain white solid (59.3 mg, 41%). ^{1}H NMR (600.1 MHz, D_2O): 4.21 (bdm, 1H, J_{gem} = 11.7 Hz, H-5′a); 4.27 (ddd, 1H, J_{gem} = 11.7 Hz, $J_{5'b,P}$ = 6.2 Hz, $J_{5'b,4'}$ = 3.1 Hz, H-5′b); 4.37 (bpent, 1H, $J_{4',5'a} = J_{4',5'b} = J_{4',3'} = J_{4',P}$ = 2.9 Hz, H-4′); 4.58 (dd, 1H, $J_{3',2'}$ = 5.2 Hz, $J_{3',4'}$ = 3.2 Hz, H-3′); 4.78 (dd, 1H, $J_{2',1'}$ = 6.3 Hz, $J_{2',3'}$ = 5.2 Hz, H-2′); 5.95 (d, 1H, $J_{1',2'}$ = 6.3 Hz, H-1′); 8.21 (s, 1H, H-8); ^{13}C NMR (150.9 MHz, D_2O): 67.28 (d, $J_{C,P}$ = 5.5 Hz, CH_2-5′); 72.43 (CH-3′); 75.82 (CH-2′); 85.88 (d, $J_{C,P}$ = 9.1 Hz, CH-4′); 88.14 (CH-1′); 114.73 (C-5); 139.57 (CH-8); 153.38 (C-4); 157.54 and 161.54 (C-2,6); ^{31}P NMR (202.4 MHz, D_2O): –22.45 (t, 1P, $J_{\beta,\alpha} = J_{\beta,\gamma}$ = 19.9 Hz, P_β); –10.74 (d, 1P, $J_{\alpha,\beta}$ = 19.9 Hz, P_α); –9.34 (d, 1P, $J_{\gamma,\beta}$ = 19.8 Hz, P_γ). HR-ESI-MS: *found*: 520.9985 ([M + H]$^+$, calcd for $C_{10}H_{16}O_{13}N_6P_3^-$: 520.9994).

2-Fluoroadenosine-5'-*O*-triphosphate ($r^{F}ATP$), triethylammonium salt

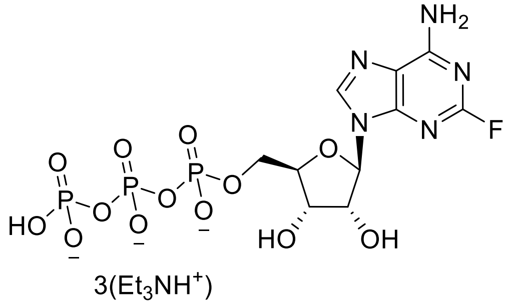

2-Fluoroadenosine-5'-*O*-triphosphate (**rA^{F}ATP**) was synthesized according to a **general procedure A** from 2-fluorodenosine (53.1 mg, 0.19 mmol) to obtain white solid (67.1 mg, 43%). ^{1}H NMR (600.1 MHz, D_2O): 4.23 (bdt, 1H, J_{gem} = 11.7 Hz, $J_{5'a,P} = J_{5'a,4'}$ = 3.7 Hz, H-5′a); 4.29 (ddd, 1H, J_{gem} = 11.7 Hz, $J_{5'b,P}$ = 6.2 Hz, $J_{5'b,4'}$ = 3.0 Hz, H-5′b); 4.40 (pent, 1H, $J_{4',5'a} = J_{4',5'b}$ =

$J_{4',3'} = J_{4',P} = 2.8$ Hz, H-4′); 4.59 (dd, 1H, $J_{3',2'} = 5.1$ Hz, $J_{3',4'} = 3.5$ Hz, H-3′); 4.79 (t, 1H, $J_{2',1'} = J_{2',3'} = 5.6$ Hz, H-2′); 6.01 (d, 1H, $J_{1',2'} = 6.0$ Hz, H-1′); 8.47 (s, 1H, H-8); ^{13}C NMR (150.9 MHz, D_2O): 67.22 (d, $J_{C,P} = 5.5$ Hz, CH_2-5′); 72.37 (CH-3′); 76.20 (CH-2′); 86.10 (d, $J_{C,P} = 9.2$ Hz, CH-4′); 88.69 (CH-1′); 118.77 (d, $J_{C,F} = 3.7$ Hz, C-5); 141.95 (CH-8); 152.53 (d, $J_{C,F} = 19.0$ Hz, C-4); 159.10 (d, $J_{C,F} = 19.6$ Hz, C-6); 161.00 (d, $J_{C,F} = 211.2$ Hz, C-2); ^{31}P NMR (202.4 MHz, D_2O): –22.54 (t, 1P, $J_{\beta,\alpha} = J_{\beta,\gamma} = 20.0$ Hz, P_β); –10.78 (d, 1P, $J_{\alpha,\beta} = 20.0$ Hz, P_α); –9.64 (d, 1P, $J_{\gamma,\beta} = 19.9$ Hz, P_γ); ^{19}F NMR (470.4 MHz, D_2O): –48.77 (s, 1F, F-2). HR-ESI-MS: *found*: 523.9787 ([M + H]$^+$, calcd for $C_{10}H_{14}O_{13}N_5FP_3^-$: 523.9791).

2-Vinyladenosine (r^{V}A)

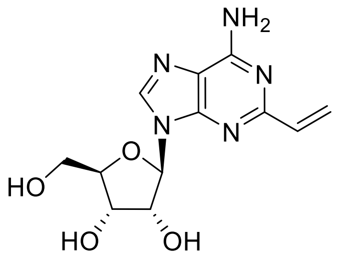

2-Iodoadenosine (109 mg, 0.28 mmol), potassium vinyltrifluoroborate (55.7 mg, 0.42 mmol), Cs_2CO_3 (27.1 mg, 0.83 mmol), $Pd(OAc)_2$ (3.1 mg, 5mol%) and TPPTS (19.7 mg, 12.5 mol%) were dissolved in H_2O/MeCN (3 mL) mixture under argon. The reaction mixture was stirred at 80 °C for 2.5 h. HPFC (SiO_2, DCM/MeOH 1:0 → 9:1) and lyophilization from H_2O/t-BuOH gave compound **r^{V}A** (33.2 mg, 41%) as a white powder. ^{1}H NMR spectra is in agreement with the literature.[4]

2-Vinyladenosine-5'-*O*-triphosphate (r^{V}ATP), triethylammonium salt

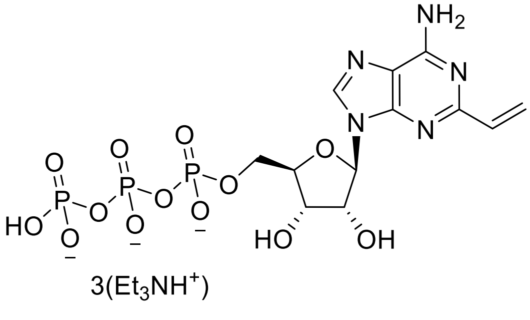

2-Vinyladenosine-5'-*O*-triphosphate (**r^{V}ATP**) was synthesized according to a **general procedure A** from 2-vinyladenosine (26 mg, 0.089 mmol) to obtain white solid (23.2 mg, 31%). ^{1}H NMR

(600.1 MHz, D_2O): 4.24 (ddd, 1H, J_{gem} = 11.7 Hz, $J_{5'a,P}$ = 4.7 Hz, $J_{5'a,4'}$ = 3.1 Hz, H-5′a); 4.29 (ddd, 1H, J_{gem} = 11.7 Hz, $J_{5'b,P}$ = 6.1 Hz, $J_{5'b,4'}$ = 3.0 Hz, H-5′b); 4.40 (pent, 1H, $J_{4',5'a}$ = $J_{4',5'b}$ = $J_{4',3'}$ = $J_{4',P}$ = 3.0 Hz, H-4′); 4.60 (dd, 1H, $J_{3',2'}$ = 5.1 Hz, $J_{3',4'}$ = 3.4 Hz, H-3′); 4.81 (bt, 1H, $J_{2',1'}$ = $J_{2',3'}$ = 5.6 Hz, H-2′); 5.78 (dd, 1H, $J_{CH2a,CH}$ = 10.8 Hz, J_{gem} = 1.1 Hz, CH=C**H2**-a); 6.17 (d, 1H, $J_{1',2'}$ = 6.0 Hz, H-1′); 6.43 (dd, 1H, $J_{CH2b,CH}$ = 17.3 Hz, J_{gem} = 1.1 Hz, CH=C**H2**-b); 6.73 (dd, 1H, $J_{CH,CH2b}$ = 17.3 Hz, $J_{CH, CH2a}$ = 10.8 Hz, C**H**=CH_2); 8.52 (s, 1H, H-8); ^{13}C NMR (150.9 MHz, D_2O): 67.28 (d, $J_{C,P}$ = 5.5 Hz, CH_2-5′); 72.41 (CH-3′); 76.28 (CH-2′); 86.00 (d, $J_{C,P}$ = 9.2 Hz, CH-4′); 88.61 (CH-1′); 119.55 (C-5); 126.38 (CH=**C**H_2); 135.98 (**C**H=CH_2); 142.55 (CH-8); 151.98 (C-4); 156.72 (C-6); 160.43 (C-2); ^{31}P NMR (202.4 MHz, D_2O): –22.60 (bt, 1P, $J_{\beta,\alpha}$ = $J_{\beta,\gamma}$ = 19.1 Hz, P_β); –10.73 (d, 1P, $J_{\alpha,\beta}$ = 19.9 Hz, P_α); –10.18 (bd, 1P, $J_{\gamma,\beta}$ = 18.5 Hz, P_γ). HR-ESI-MS: *found*: 532.0039 ($[M + H]^+$, calcd for $C_{12}H_{17}O_{13}N_5P_3^-$: 532.0041).

2-Ethynyladenosine (r^{E}A)

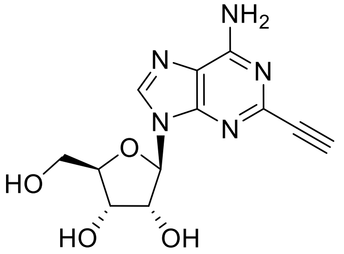

2-Iodoadenosine (216mg, 0.55 mmol), $PdCl_2(PPh_3)_2$ (38.6 mg, 10 mol%), CuI (10.5 mg, 10 mol%) were dissolved in dry DMF (4 mL) under argon. Trimethylsilylacetylene (0.16 mL, 1.1 mmol) and TEA (0.15 mL, 1.1 mmol) were added dropwise, and reaction mixture was stirred at 80 °C for 1 h. HPFC (SiO_2, DCM/MeOH 1:0 → 9:1) and lyophilization from H_2O/t-BuOH gave compound **r^{E}A** (76.3 mg, 48%) as a pale-brown powder. ^{1}H NMR spectra is in agreement with the literature.[5]

2-Ethynyladenosine-5'-*O*-triphosphate (r^{E}ATP), triethylammonium salt

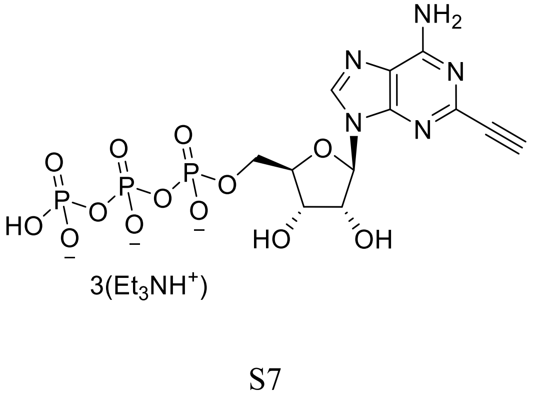

2-Ethynyladenosine-5'-*O*-triphosphate (**r^{E}ATP**) was synthesized according to a **general procedure A** from 2-ethynyladenosine (48.2 mg, 0.17 mmol) to obtain white solid (30.9 mg, 22%). ^{1}H NMR spectra is in agreement with the literature.[6]

2',3',5'-tri-*O*-Acetyl-2-iodoadenosine (AcO^{I}A)

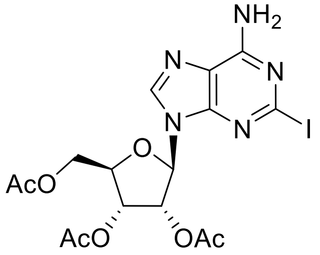

To suspension of 2-iodoadenosine (201 mg, 0.51 mmol) and DMAP (5 mg, 0.041 mmol) in a mixture of MeCN (8 mL) and TEA (0.28 mL, 2.04 mmol), acid anhydride (0.17 mL, 1.79 mmol) was added and let to stir for 1 h. After the completion of the reaction, MeOH was added and stirring was continued for 5 min. HPFC (SiO_2, EtOAC) and lyophilization from H_2O/t-BuOH gave compound **AcOA^{I}** (259.0 mg, 98%) as a white powder. ^{1}H NMR spectra is in agreement with the literature.[7]

2',3',5'-tri-*O*-Acetyl-2-methyladenosine (AcO^{Me}A)

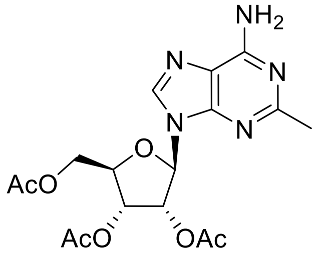

AcOA^{I} (176 mg, 0.34 mmol) and $Pd(PPh_3)_4$ (39.2 mg, 10 mol%) were dissolved in NMP (0.9 mL) and Me_4Sn (182 mg, 1.02 mmol) was added under argon. The mixture was heated at 60 °C overnight. After the completion of the reaction, it was partitioned between ethyl acetate and water, extracted, dried over anhydrous Na_2SO_4 and evaporated. HPFC (SiO_2, cH → DCM/MeOH 1:0 → 1:4) gave compound **AcO^{Me}A** (104 mg, 75%) as a glassy solid. Characterization was done after deprotection.

2-Methyladenosine (r^{Me}A)

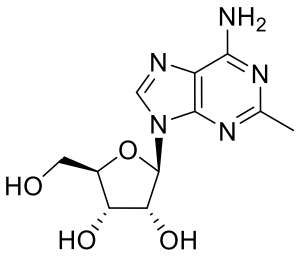

AcO^{Me}A (104 mg, 0.26 mmol) and K_2CO_3 (106 mg, 0.77 mmol) were dissolved in MeOH and stirred at rt overnight. HPFC (SiO_2, DCM/MeOH 1:0 → 9:1) and lyophilization from H_2O/t-BuOH gave compound r^{Me}**A** (44.8 mg, 62%) as a white powder. ^{1}H NMR spectra is in agreement with the literature.[8]

2-Methyladenosine-5'-*O*-triphosphate (A^{Me}TP), triethylammonium salt

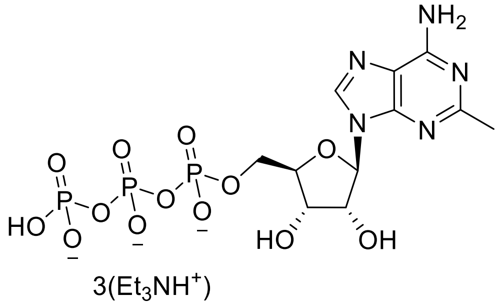

2-Methyladenosine-5'-*O*-triphosphate (**rA^{Me}TP**) was synthesized according to a **general procedure A** from 2-methyladenosine (49.3 mg, 0.18 mmol) to obtain white solid (63.9 mg, 44%). ^{1}H NMR (401 MHz, D_2O): 2.53 (s, 3H, CH_3); 4.19 (ddd, 1H, J_{gem} = 11.7 Hz, $J_{5'a,P}$ = 4.5 Hz, $J_{5'a,4'}$ = 3.0 Hz, H-5'a); 4.27 (ddd, 1H, J_{gem} = 11.7 Hz, $J_{5'b,P}$ = 6.1 Hz, $J_{5'b,4'}$ = 2.8 Hz, H-5'b); 4.39 (pent, 1H, $J_{4',5'a} = J_{4',5'b} = J_{4',3'} = J_{4',P}$ = 2.8 Hz, H-4'); 4.58 (dd, 1H, $J_{3',2'}$ = 5.1 Hz, $J_{3',4'}$ = 3.2 Hz, H-3'); 4.79 (m, 1H, H-2'); 6.11 (d, 1H, $J_{1',2'}$ = 6.2 Hz, H-1'); 8.48 (s, 1H, H-8); ^{13}C NMR (150.9 MHz, D_2O): 26.08 (CH_3); 67.05 (d, $J_{C,P}$ = 5.3 Hz, CH_2-5'); 72.33 (CH-3'); 76.14 (CH-2'); 86.13 (d, $J_{C,P}$ = 9.1 Hz, CH-4'); 88.21 (CH-1'); 118.78 (C-5); 141.36 (CH-8); 152.13 (C-4); 157.44 (C-6); 165.16 (C-2); ^{31}P NMR (202.4 MHz, D_2O): –21.08 (m, 1P, P_β); –10.40 (d, 1P, $J_{\alpha,\beta}$ = 19.3 Hz, P_α); –5.39 (bs, 1P, P_γ). HR-ESI-MS: *found*: 520.0040 ($[M + H]^+$, calcd for $C_{11}H_{17}O_{13}N_5P_3^-$: 520.0041).

2. Enzymatic synthesis

General remarks

All polyacrylamide gel electrophoresis (PAGE) gels were analyzed by fluorescence imaging using Amersham Typhoon (Cytiva). Single-stranded DNA oligonucleotides for preparation of double-stranded DNA templates, synthetic oligonucleotides (non-modified, 5’-6-FAM labeled with 6-carboxyfluorescein) were purchased from Generi Biotech (Czech Republic), Eurofins, IDT or Biomers. DNase I, T7 RNA polymerase (50 U/µL) and HiScribe T7 High Yield RNA Synthesis Kit with the corresponding transcription buffer, RiboLock RNase Inhibitor, the natural ribonucleoside triphosphates (rNTPs), RiboRuler Low RangeRNA ladder, precision RNA mass marker were purchased from New England Biolabs, ThermoScientific and FUTUREsynthesis. Engineered DNA polymerases TGK, SFM4-3 and 2M were prepared according to the literature protocols[9,10,11,12] and were expressed in-house and kindly donated by different group members. Fluorescent label (**Cy3-N$_3$**) was purchased from Jena BioScience and fluorescent thiol (**CM-SH**) was a courtesy gift by a group member synthesized according to previously published procedure[13]. Poly(A) yeast polymerase ScPAP (*Saccharomyces cerevisiae*) was purchased from Jena BioScience. Samples after reactions were purified with Monarch RNA purification kits (50 µg) (New England Biolabs) or/and QIAquick Nucleotide Removal Kit (QIAGEN) from BioTech (Czech Republic). RNase/DNase free solutions for biochemical reactions were prepared using Milli-Q water, that was treated with DEPC and sterilized by autoclaving. The reaction was stopped by the addition of 2X PAGE stop solution (95% [v/v] formamide, 0.5 mM EDTA, 0.025% [w/v] bromophenol blue, 0.025% [w/v] xylene cyanol FF, 0.025% SDS in Mili-Q water). Samples after reactions were analyzed by 10% PAGE (acrylamide/bisacrylamide 19:1, 20% urea) or 15% PAGE (acrylamide/bisacrylamide 19:1, 30% urea) or 20% PAGE (acrylamide/bisacrylamide 19:1, 40% urea) under denaturing conditions (60 min, 30 mA, 50 °C, 1X TBE buffer). Non-labeled products were visualized after the staining with SYBR Gold (Invitrogen). Concentrations of the prepared RNA solutions were calculated using on-line tool at https://www.atdbio.com/tools/oligo-calculator and A260 values measured on Nanodrop (Nanophotometer N60 (Implen)). Fluorescence spectra were measured in a 100 µL quartz cuvette at room temperature on a Fluoromax 4 spectrofluorometer (HORIBA Scientific). LC-ESI-MS spectra were acquired on Agilent 1290 Infinity II Bio system with DAD detector and mass spectrometer MSD XT. LC-ESI-MS analysis

of oligonucleotides were carried out according to standard procedures using mobile phases A (12.2 mM Et_3N, 300 mM HFIP in H_2O) and B (12.2 mM Et_3N, 300 mM HFIP in H_2O in 100% MeOH) by 10 min gradient from 5% B to 100% B in A using bioZen 1.7 µm oligo column 2.1 X 50 mm (Phenomenex) on Agilent UHPLC Bio system. Deconvolutions of LC-ESI-MS spectra were carried out using UniDec program.[14]

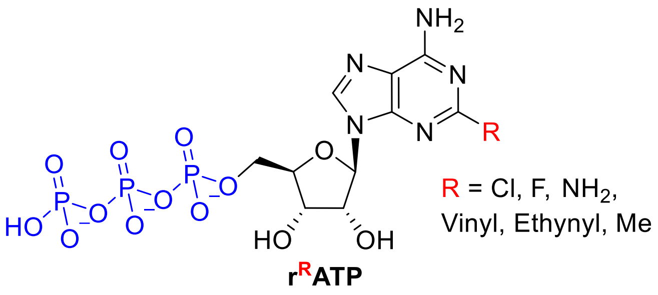

Figure S1. Structures of modified **r^{R}ATP** that were used in this study.

2.1 List of oligonucleotides used in this study

Table S1. List of primers and templates used in this study and list of synthesized RNA.

Name	Modification	Sequence (5'→ 3')	Length (nt)
**87DNA**	Sense –	TAATACGACTCACTATAGGGCCCTTCGCCAACTTGCAGAGACGGTCGGGTCCAGATATTCGTATCTGTCGAGTAGAGTGTGGGCTCC	87
	Anti-sense 5'-[2'-*O*-Me-G]	[mG][mG]AGCCCACACTCTACTCGACAGATACGAATATCTGGACCCGACCGTCTCTGCAAGTTGGCGAAGGGCCCTATAGTGAGTCGTATTA	
**70RNA_^{R}A**	–	GGGCCCUUCGCC**^{R}A^{R}A**CUUGC**^{R}A**G**^{R}A**G**^{R}A**CGGUCGGGUCC**^{R}A**G**^{R}A**U**^{R}A**UUCGU**^{R}A**UCUGUCG**^{R}A**GU**^{R}A**G**^{R}A**GUGUGGGCUCC	70
**52DNA**	Sense –	TAATACGACTCACTATAGGGCCCGTATGTTACTTGCTCTTATCGTCTCTCGC	52
	Anti-sense 5'-[2'-*O*-Me-G]	[mG]CGAGAGACGATAAGAGCAAGTAACATACGGGCCCTATAGTGAGTCGTATTA	
**35RNA**	–	GGGCCCGUAUGUUACUUGCUCUUAUCGUCUCUCGC	35

**rPrim^{15}**	5’-6-FAM	CAUGGGCGGCAUGGG	15
**Temp^{31}**	–	CTAGCATGAGCTCAGTCCCATGCCGCCCATG	31
**31RNA_^{R}A**	5’-6-FAM	CAUGGGCGGCAUGGG^{R}**A**CUG^{R}**A**GCUC^{R}**A**UGCU^{R}**A**G	31
**Temp^{31_SNI}**	–	AGAAGAGAGAGACAATCCCATGCCGCCCATG	31
**16RNA_SNI_ ^{R}A**	5’-6-FAM	CAUGGGCGGCAUGGG^{R}**A**	16
**31RNA_SNI_^{R}A**	5’-6-FAM	CAUGGGCGGCAUGGG^{R}**A**UUGUCUCUCUCUUCU	31

Primer and T7 RNA promotor region is underlined

2.2 *In vitro* transcription

2.2.1 Preparation of double-stranded DNA templates

A solution of complementary single-stranded DNA oligonucleotides (Table S1) (100 μM each) in water was heated up to 95 °C for 5 min in a thermal cycler, and then slowly cooled down to 25 °C. The resulting **87DNA** and **52DNA** (50 μM) was used as a template for transcription reactions.

2.2.2 Synthesis of 70mer RNA

In vitro transcription reactions were performed in the total volume of 10 μL in 40 mM Tris buffer (pH 7.9) containing modified **r^{R}ATP** (4 mM), three natural rNTPs (4 mM), Ribolock RNase inhibitor (0.25 μL, 1 U/μL), DMSO (5%), **87DNA** template (Table S1) (2 μM), T7 RNA polymerase (HiScribe T7 High Yield RNA Synthesis Kit). In the negative control experiment, water was used instead of the solution of modified **r^{R}ATP**, and in the positive control the natural rNTP (4 mM). The transcription reactions were performed at 37 °C for 3 h. Following, the template was removed by treatment with 1 U/μL DNase I (2 μL) for 1 h at 37 °C. EDTA (1 μL/10 μL reaction) 0.5 M was added, and the samples were then purified with Monarch kit as per supplier’s protocol and eluted with 40 μL of H_2O and concentration was determined using NanoDrop. 5 μL of sample was mixed with RNA loading dye (5 μL) and heated at 95 °C for 5 minutes. The samples were then analyzed by gel electrophoresis on 15% denaturing PAGE, stained with SYBR Gold and visualized using fluorescence imaging (Figure 1B) and further analyzed by LC-MS (Table S2, Entry 1-7, chromatograms and copies of MS spectra in Section 5).

2.2.3 Synthesis of 35mer RNA

In vitro transcription reactions were performed in the total volume of 10 µL in 40 mM Tris buffer (pH 7.9) containing all four natural **rNTPs** (5 mM), Ribolock RNase inhibitor (0.25 µL, 1 U/µL), DMSO (10%), **52DNA** template (Table S1) (1 µM), T7 RNA polymerase (1 µL, 50 U/µL). The transcription reactions were performed at 37 °C for 16 h. Following, the template was removed by treatment with 1 U/µL DNase I (2 µL) for 1 h at 37 °C. EDTA (1 µL/10 µL reaction) 0.5 M was added, and the samples were then purified with Monarch kit as per supplier's protocol and eluted with 40 µL of H_2O and concentration was determined using NanoDrop. 5 µL of sample was mixed with RNA loading dye (5 µL) and heated at 95 °C for 5 minutes. The sample was then analyzed by gel electrophoresis on 15% denaturing PAGE, stained with SYBR Gold and visualized using fluorescence imaging (data not shown) and further analyzed by LC-MS (Table S2, Entry 8, chromatograms and copies of MS spectra in Section 5).

Table S2. Mass after UPLC-MS analysis

Entry	RNAs after transcription	Mw [Da] calculated	Mw [Da] observed	Δ [Da]	Figures
1	**70RNA_A**	22763	22790^{a}	27	S12, S13
2	**70RNA_^{Cl}A**	23176	23200^{a}	24	S14, S15
3	**70RNA_^{NH2}A**	22943	22970^{a}	27	S16, S17
4	**70RNA_^{F}A**	22979	23010^{a}	31	S18, S19
5	**70RNA_^{V}A**	23075	23060/23400^{b}	15/325	S20, S21
6	**70RNA_^{E}A**	23051	23030/23080^{a}	21/29	S22, S23
7	**70RNA_^{Me}A**	22931	22960^{a}	29	S24, S25
8	**35RNA**	11261	11300^{c}	39	S26, S27

a – product + Na^{+}; b – product + rAMP; c – product + K^{+}

2.3 Primer extension experiments (PEX)

2.3.1 Multiple incorporations of modified r^{R}ATPs

Reaction mixture (10 µL) was prepared by mixing primer **rPrim**[15] (Table S1) (4 µM), template **Temp**[31] (Table S1) (4.8 µM), engineered polymerase (TGK, 2M or SFM4-3) (0.5 µM), natural rNTPs (0.4 mM), either natural or modified rATP (0.2 mM) and reaction buffer (10X, 1 µL). In the negative control experiment, water was used instead of the solution of modified rNTP. The

reaction was incubated at 60 (2M, SFM4-3) or 65 °C (TGK) for 2 h. The reaction was stopped by the addition of 2X PAGE stop solution and heated at 95 °C for 5 min. Aliquots with **31NA_^{R}A** (DNA RNA hybrid) (10 µL) were subjected to vertical electrophoresis in 15% denaturing PAGE. The gel was visualized by fluorescence imaging (Figure S2-4).

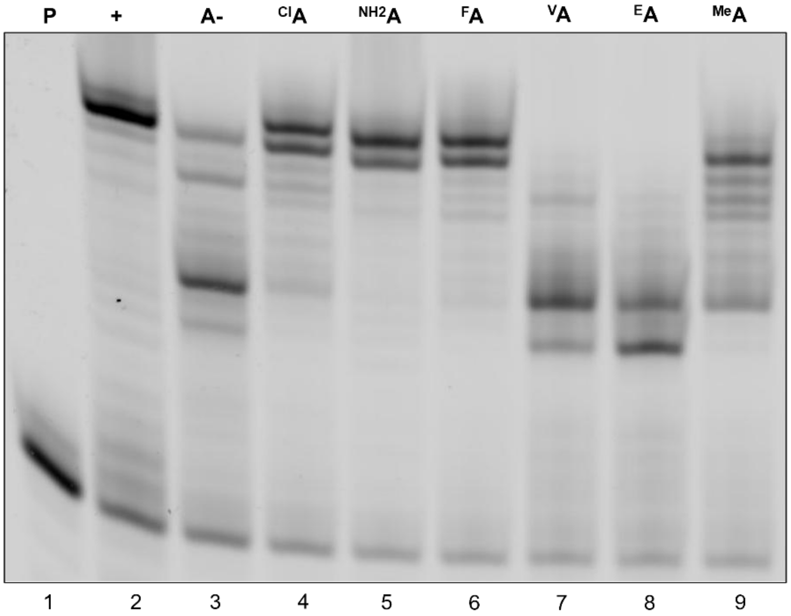

Figure S2. 15% dPAGE of 5'-(6-FAM)-labelled **31NA_^{R}A** after PEX reaction with TGK polymerase at 65 °C. (lane 1, **P**) primer, (lane 2, +) positive control (four natural rNTPs), (lane 3, **A-**) negative control (rGTP, rCTP, rUTP), (lane 4, **^{Cl}A**) chloro modification incorporation (**r^{Cl}ATP**, rGTP, rCTP, rUTP), (lane 5, **^{NH2}A**) amino modification incorporation (**r^{NH2}ATP**, rGTP, rCTP, rUTP), (lane 6, **^{F}A**) fluoro modification incorporation (**r^{F}ATP**, rGTP, rCTP, rUTP), (lane 7, **^{V}A**) vinyl modification incorporation (**r^{V}ATP**, rGTP, rCTP, rUTP), (lane 8, **^{E}A**) ethynyl modification incorporation (**r^{E}ATP**, rGTP, rCTP, rUTP), (lane 9, **^{Me}A**) methyl modification incorporation (**r^{Me}ATP**, rGTP, rCTP, rUTP).

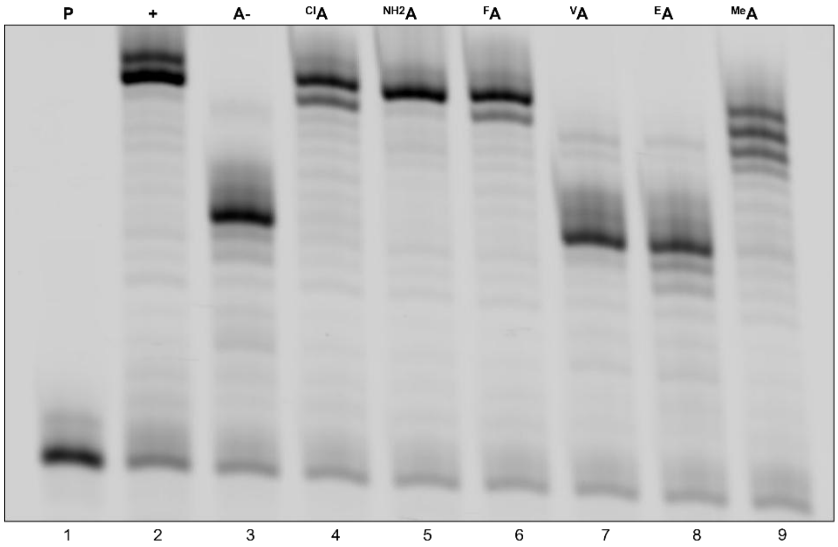

Figure S3. 15% dPAGE of 5’-(6-FAM)-labelled **31NA_^{R}A** after PEX reaction with 2M polymerase at 60 °C. (lane 1, **P**) primer, (lane 2, +) positive control (four natural rNTPs), (lane 3, **A-**) negative control (rGTP, rCTP, rUTP), (lane 4, **^{Cl}A**) chloro modification incorporation (**r^{Cl}ATP**, rGTP, rCTP, rUTP), (lane 5, **^{NH2}A**) amino modification incorporation (**r^{NH2}ATP**, rGTP, rCTP, rUTP), (lane 6, **^{F}A**) fluoro modification incorporation (**r^{F}ATP**, rGTP, rCTP, rUTP), (lane 7, **^{V}A**) vinyl modification incorporation (**r^{V}ATP**, rGTP, rCTP, rUTP), (lane 8, **^{E}A**) ethynyl modification incorporation (**r^{E}ATP**, rGTP, rCTP, rUTP), (lane 9, **^{Me}A**) methyl modification incorporation (**r^{Me}ATP**, rGTP, rCTP, rUTP).

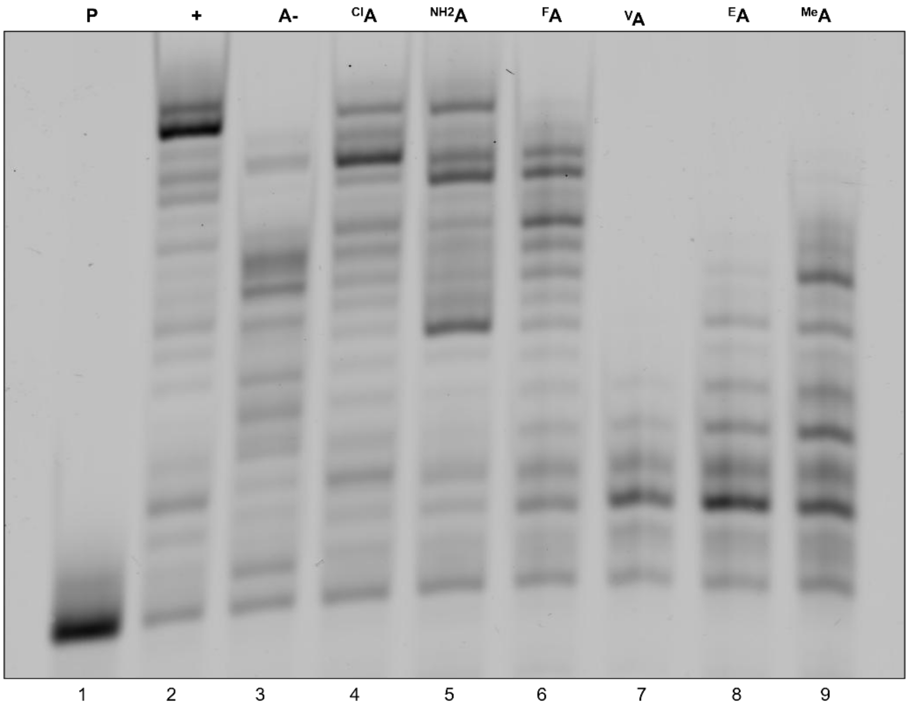

Figure S4. 15% dPAGE of 5'-(6-FAM)-labelled **31NA_^{R}A** after PEX reaction with SFM4-3 polymerase at 60 °C. (lane 1, **P**) primer, (lane 2, +) positive control (four natural rNTPs), (lane 3, **A-**) negative control (rGTP, rCTP, rUTP), (lane 4, **^{Cl}A**) chloro modification incorporation (**r^{Cl}ATP**, rGTP, rCTP, rUTP), (lane 5, **^{NH2}A**) amino modification incorporation (**r^{NH2}ATP**, rGTP, rCTP, rUTP), (lane 6, **^{F}A**) fluoro modification incorporation (**r^{F}ATP**, rGTP, rCTP, rUTP), (lane 7, **^{V}A**) vinyl modification incorporation (**r^{V}ATP**, rGTP, rCTP, rUTP), (lane 8, **^{E}A**) ethynyl modification incorporation (**r^{E}ATP**, rGTP, rCTP, rUTP), (lane 9, **^{Me}A**) methyl modification incorporation (**r^{Me}ATP**, rGTP, rCTP, rUTP).

2.3.2 Single nucleotide incorporation (SNI) experiments

Reaction mixture (7 µL) was prepared by mixing primer **rPrim^{15}** (Table S1) (4 µM), **Temp^{31_SNI}** (Table S1) (4.8 µM), 2M or TGK DNA polymerase (0.71 µM), either natural or modified rATP (0.29 mM) and reaction buffer (10X, 1 µL). In the negative control experiment, water was used instead of the solution of modified rNTP. The reaction was incubated at 60 °C for 30 min. After this time natural rNTPs (0.8 mM) except rATP were added together with additional 2M polymerase (0.3 µM) and reaction was continued for additional 1 h. The reaction was stopped by the addition of 2X PAGE stop solution. Aliquots **31NA_SNI_^{R}A** (DNA·RNA hybrid) (10 µL) were subjected

to vertical electrophoresis in 15% denaturing PAGE. The gel was visualized by fluorescence imaging (Figure S5-S6).

Experiments concerning positive **31NA_SNI_A(+)** and negative **31NA_SNI_A(-)** controls were also repeated in triplicate experiments for both DNA polymerases and further analyzed by 15% dPAGE (see Section 6, Figure S60 for 2M, Figure S61 for TGK) and UPLC-MS (see Section 5, Figures S50 – S55 for 2M, Figures S56 – S59 for TGK and Section 6, Table S4).

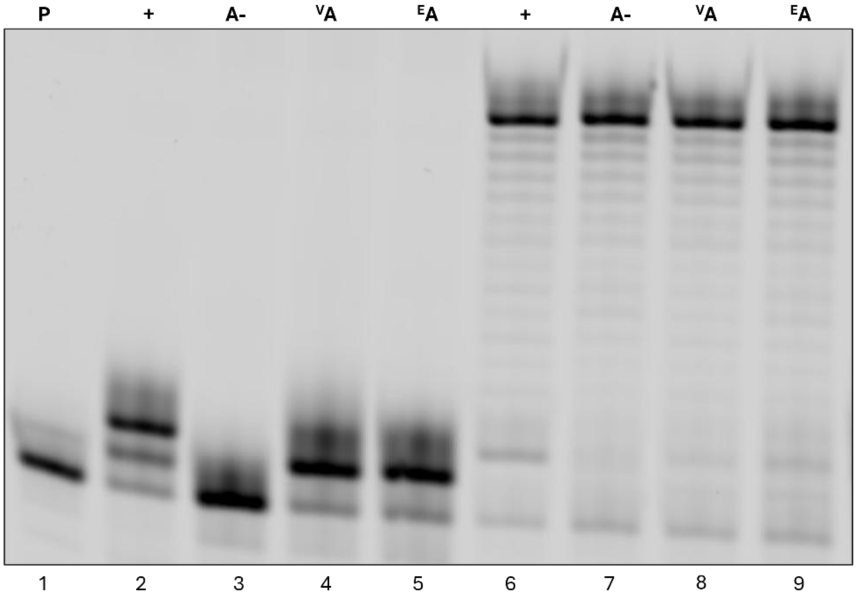

Figure S5. 15% dPAGE of 5'-(6-FAM)-labelled **16NA_^{R}A** and **31NA_^{R}A** after SNI and PEX reactions with 2M polymerase. (lane 1, **P**) primer, (lane 2, +) positive control (natural **rATP**), (lane 3, **A-**) negative control (water), (lane 4, ^{V}**A**) single vinyl modification incorporation (**r^{V}ATP**), (lane 5, ^{E}**A**) single ethynyl modification incorporation, (**r^{E}ATP**), (lane 6-9, +) PEX with addition of rGTP, rCTP, rUTP.

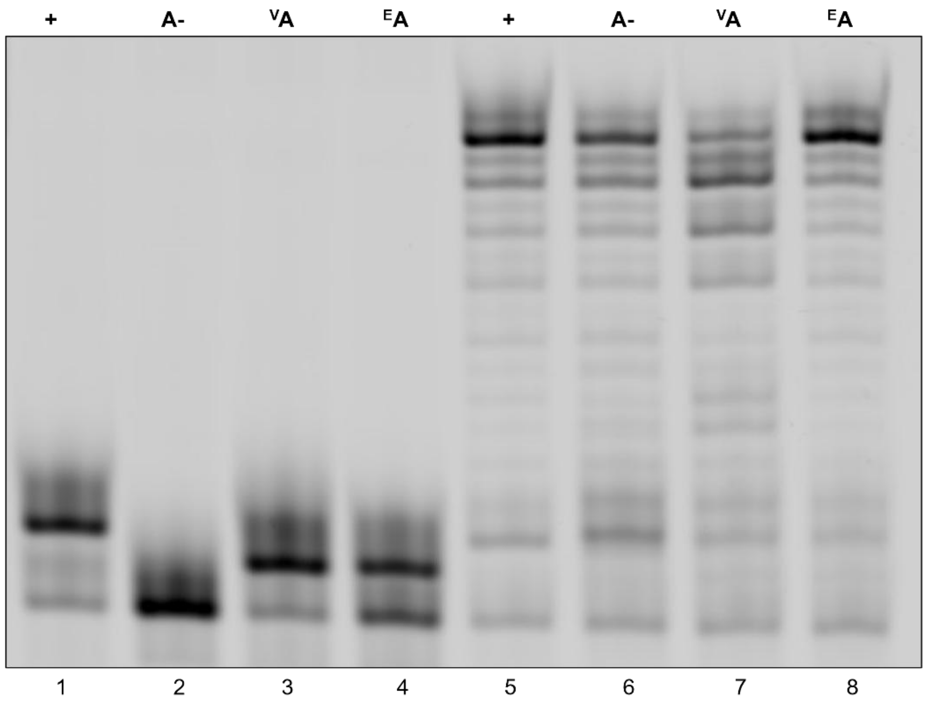

Figure S6. 15% dPAGE of 5’-(6-FAM)-labelled **16NA_^{R}A** and **31NA_^{R}A** after SNI and PEX reactions with TGK polymerase. (lane 1, **P**) primer, (lane 2, +) positive control (natural **rATP**), (lane 3, **A-**) negative control (water), (lane 4, ^{V}**A**) single vinyl modification incorporation (**r^{V}ATP**), (lane 5, ^{E}**A**) single ethynyl modification incorporation, (**r^{E}ATP**), (lane 6-9, +) PEX with addition of rGTP, rCTP, rUTP.

2.3.3 Semi-preparative preparation of modified RNA

For mass detection analysis, analytical reactions were scaled up four times. For post-synthetic modifications, reaction was scaled up ten times. In both cases after the reaction the template was removed by treatment with 1 U/µL Turbo DNase for 30 min at 37 °C. The samples were purified with QIAquick nucleotide removal kit, eluted with 100 µL of MilliQ water and concentration was determined using NanoDrop and further analyzed by LC-MS (Table S3, Entry 1-9).

2.4 Post-synthetic modifications of modified RNA

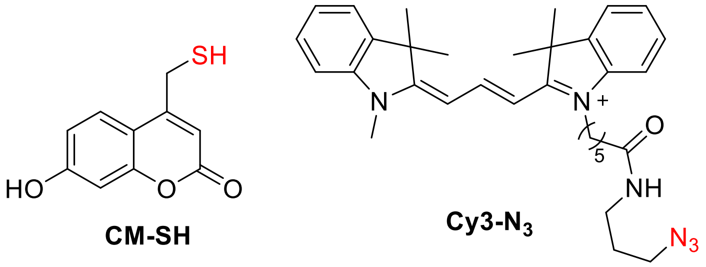

Figure S7. Structures of fluorescent labels (**CM-SH** and **Cy3-N$_3$**) that were used in this study.

2.4.1 Click reaction of 31RNA_SNI_^{E}A

31RNA_SNI_^{E}A (0.2 nmol) was dissolved in H_2O:DMSO:*t*BuOH mixture (9:3:1, 8 µL). The reaction mixture was prepared by adding azide (**Cy3-N$_3$**, 50 mM in DMSO, 5.6 µL), sodium ascorbate (5 mM in H_2O, 4 µL) and CuI solution, which was prepared just before by mixing CuBr (100 mM in DMSO, 0.4 µL) with TBTA (tris(benzyltriazolylmethyl)amine) (100 mM in DMSO, 2 µL). Total volume of the reaction is 20 µL. Negative control experiments were performed using the same conditions, but instead of **31RNA_SNI_^{E}A**, natural **31RNA_SNI_A** was used. The reaction mixture was incubated at 37 °C and 500 rpm overnight. The reaction was stopped by the addition of 2X PAGE stop solution. Aliquots (10 µL) were subjected to vertical electrophoresis in 15% denaturing PAGE. The gel was visualized by fluorescence imaging (Figure 4B) and further analyzed by LC-MS (Table S3, Entry 10).

For steady-state fluorescence measurements the product of click reaction was purified with QIAquick nucleotide removal kit, eluted with 100 µL of MilliQ water and concentration was determined using NanoDrop. Concentration of RNA was adjusted to 1 µM in MilliQ H_2O. Emission spectra were recorded by adding 10 µL of **31RNA_SNI_^{Cy3}A** solution. The excitation wavelength for **31RNA_SNI_^{Cy3}A** was 540 nm and the range of the emission spectra was 550-750 nm. Control experiments were performed using **31RNA_SNI_^{E}A** and non-modified **31RNA_SNI_A** (after negative control reaction) following the same procedure (Figure 4C).

2.4.2 Thiol-ene addition reaction of 31RNA_SNI_^{V}A

31RNA_SNI_^{V}A (0.2 nmol) was dissolved in H_2O mixture (5 µL). The reaction mixture was prepared by adding **CM-SH** (25 µL, 70 mM in 0.5 M TEAA buffer, pH 7). Total volume of the reaction is 30 µL. Negative control experiments were performed using the same conditions, but instead of **31RNA_SNI_^{V}A**, natural **31RNA_SNI_A** was used. The reaction mixture was incubated at 37 °C and 500 rpm for 3 days. The reaction was stopped by the addition of 2X PAGE stop solution. Aliquots (10 µL) were subjected to vertical electrophoresis in 20% denaturing PAGE. The gel was visualized by fluorescence imaging (Figure 4E) and further analyzed by LC-MS (Table S3, Entry 11).

Table S3. Mass after UPLC-MS analysis

Entry	RNAs after PEX	Mw [Da] calculated	Mw [Da] observed	Δ [Da]	Figures
1	**31RNA_A**	10566	10890^{a}	324	S28, S29
2	**31RNA_^{Cl}A**	10704	10700/11063^{b}	4/359	S30, S31
3	**31RNA_^{NH2}A**	10626	10622/10966^{a}	4/340	S32, S33
4	**31RNA_^{F}A**	10638	10633/10980^{a}/10288^{c}	5/342/350	S34, S35
5	**31RNA_^{Me}A**	10622	10270^{c}/10610	352/12	S36, S37
6	**16RNA_SNI_^{V}A**	5778	5776	2	S38, S39
7	**16RNA_SNI_^{E}A**	5776	5774	2	S40, S41
8	**31RNA_SNI_^{V}A**	10404	10401	3	S42, S43
9	**31RNA_SNI_^{E}A**	10402	10400	2	S44, S45
10	**31RNA_SNI_^{CM}A**	10612	10610	2	S46, S47
11	**31RNA_SNI_^{Cy3}A**	10941	10940	1	S48, S49

a – product + rAMP; b – product + rGMP; c – product – rGMP.

2.5 Polyadenylation experiments

The experiment part is the same as in the literature.[15] Yeast Poly(A) polymerase (ScPAP) was used together with synthesized **35RNA** sequence (Section 2.2.3, Table S1). The reaction was performed in total volume of 25 µL which contains 0.2 µM **35RNA** (Table S1), 0.5 mM **rATP** or modified **r^{R}ATP**, and 600 U ScPAP in 1X reaction buffer. Reactions were incubated for 20 min at 37 °C, then 10 min at 65 °C. The samples were then purified with Monarch kit as per supplier's protocol, eluted with 40 µL of MilliQ water and concentration was determined using NanoDrop. 5 µL of sample was mixed with RNA loading dye (5 µL) and heated at 95 °C for 5 min. The

samples (**35RNA_poly^{R}A**) were then analyzed by gel electrophoresis on 10% denaturing PAGE, stained with SYBR Gold and visualized by fluorescence imaging (Figure 5B).

2.5.1 Semi-preparative preparation of modified 35RNA_polyA^{E}A and 35RNA_polyA^{V}A

For post-synthetic modifications, reaction was done in the same manner as in section 2.5, but instead of **r^{E}ATP** or **r^{V}ATP** the mixture with natural **rATP** was used (9:1, **rATP**:**r^{E}ATP** or **r^{V}ATP)** for the synthesis of **35RNA_polyA^{E}A** and **35RNA_polyA^{V}A**. Also, reaction was scaled up ten times. The samples were purified with Monarch kit as per supplier's protocol, eluted with 40 µL of MilliQ water and concentration was determined using NanoDrop.

2.6 Post-synthetic modifications of modified poly(A)

2.6.1 Click reaction of 35RNA_polyA^{E}A

35RNA_polyA^{E}A (5.53 µL, 318 ng/µL) was mixed with DMSO:*t*BuOH (3:1, 2.47 µL). The reaction mixture was prepared by adding azide (**Cy3-N$_3$**, 50 mM in DMSO, 5.6 µL), sodium ascorbate (5 mM in H_2O, 4 µL) and CuI solution, which was prepared just before by mixing CuBr (100 mM in DMSO, 0.4 µL) and TBTA (100 mM in DMSO, 2 µL). Total volume of the reaction is 20 µL. Negative control experiments were performed using the same conditions, but instead of **35RNA_polyA^{E}A**, natural **35RNA_polyA** was used. The reaction mixture was incubated at 37 °C and 500 rpm overnight. The reaction was stopped by the addition of 2X PAGE stop solution and heated at 95 °C for 5 min. Aliquots (10 µL) were subjected to vertical electrophoresis in 15% denaturing PAGE. The gel was visualized by fluorescence imaging (Figure 6B).

For steady-state fluorescence measurements the product of click reaction was purified with QIAquick nucleotide removal kit, eluted with 100 µL of MilliQ water and concentration was determined using NanoDrop. Emission spectra were recorded in MilliQ H_2O by adding 45 ng of **35RNA_polyA^{Cy3}A**. The excitation wavelength for **35RNA_polyA^{Cy3}A** was 540 nm and the range of the emission spectra was 550-750 nm. Control experiments were performed using **35RNA_polyA^{E}A** and non-modified **35RNA_polyA** (after negative control reaction) following the same procedure (Figure 6C).

2.6.2 Thiol-ene addition reaction of 35RNA_polyA^{V}A

To **35RNA_polyA^{V}A** (3.86 µL, 341 ng/µL) additional H_2O (1.14 µL) was added. The reaction mixture was prepared by adding **CM-SH** (25 µL, 70 mM in 0.5 M TEAA buffer, pH 7). Total volume of the reaction is 30 µL. Negative control experiments were performed using the same conditions, but instead of **35RNA_polyA^{V}A**, natural **35RNA_polyA** was used. The reaction mixture was incubated at 37 °C and 500 rpm for 3 days. The reaction was stopped by the addition of 2X PAGE stop solution and heated at 95 °C for 5 min. Aliquots (10 µL) were subjected to vertical electrophoresis in 15% denaturing PAGE. The gel was visualized by fluorescence imaging (Figure 6E).

For steady-state fluorescence measurements the product of thiol-ene reaction was purified with QIAquick nucleotide removal kit (eluted with 100 µL of MilliQ water) and additional Monarch kit purification (eluted with 40 µL of MilliQ water). Concentration was determined using NanoDrop. Emission spectra were recorded in MilliQ water by adding 70 ng of **35RNA_polyA^{CM}A**. The excitation wavelength for **35RNA_polyA^{CM}A** was 370 nm and the range of the emission spectra was 380-650 nm. Control experiments were performed using **35RNA_poly^{V}A** and non-modified **35RNA_polyA** (after negative control reaction) following the same procedure (Figure 6F).

3. Uncropped gels

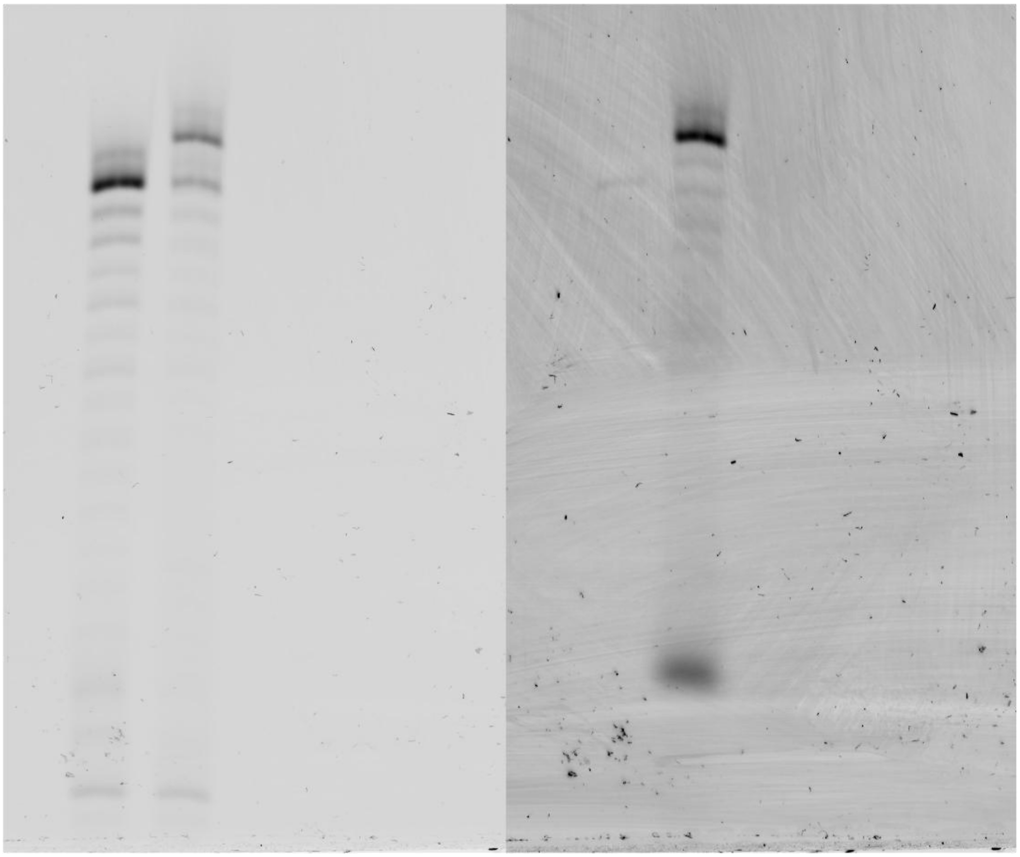

Figure S8. Uncropped gel of Figure 4B.

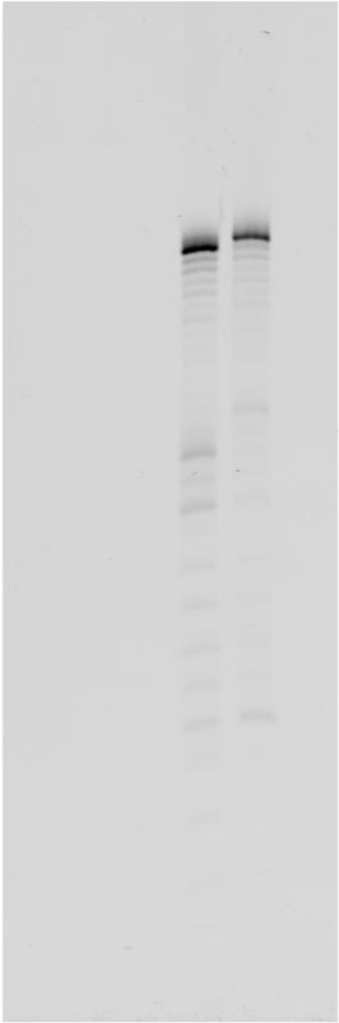

Figure S9. Uncropped gel of Figure 4E.

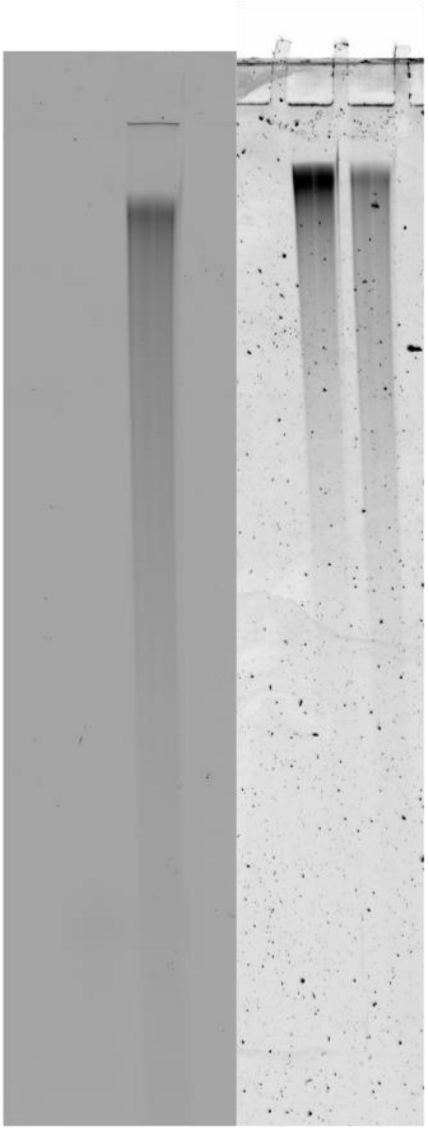

Figure S10. Uncropped gel of Figure 6B.

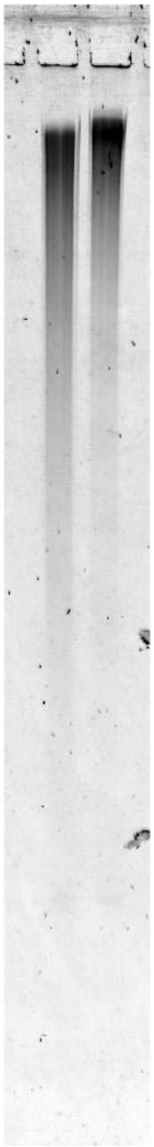

Figure S11. Uncropped gel of Figure 6E.

4. Copies of NMR spectra

^{1}H, ^{13}C, ^{31}P{^{1}H} NMR spectra of **r^{Cl}ATP**

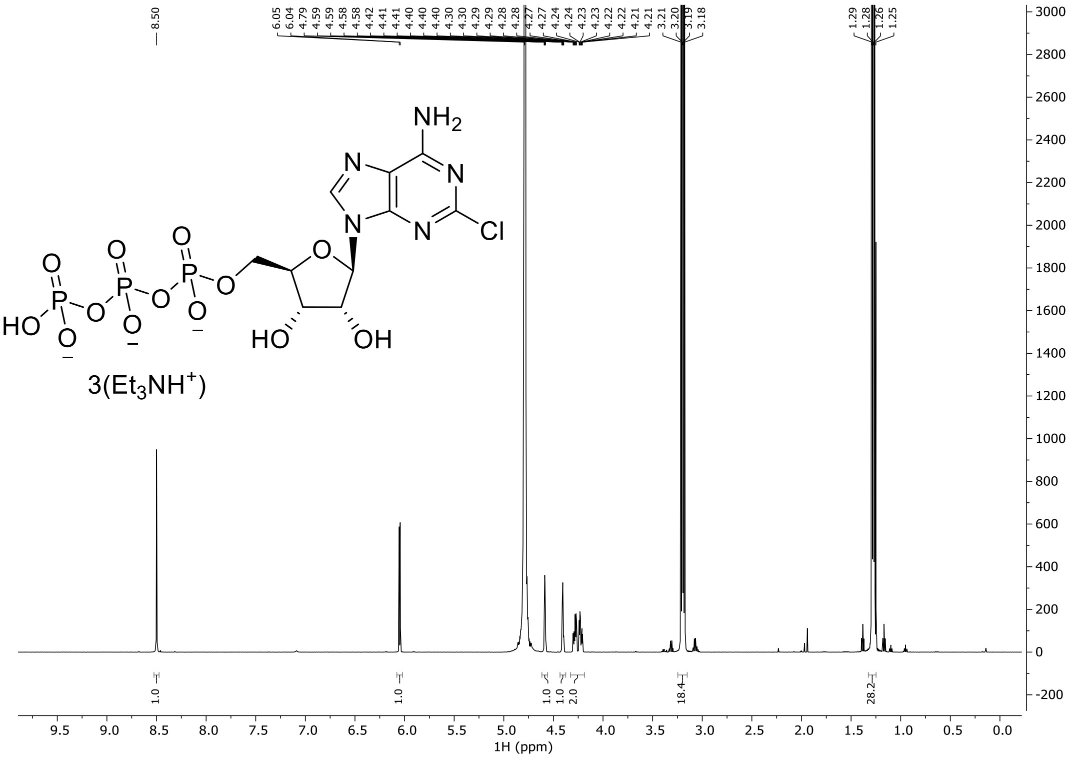

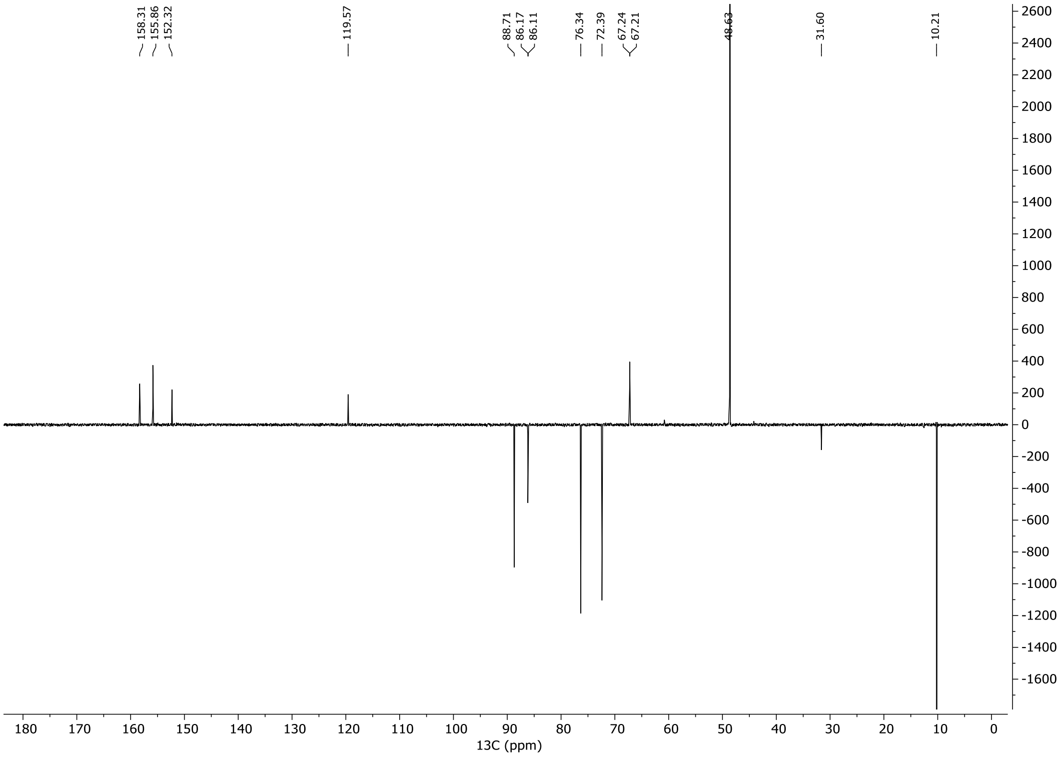

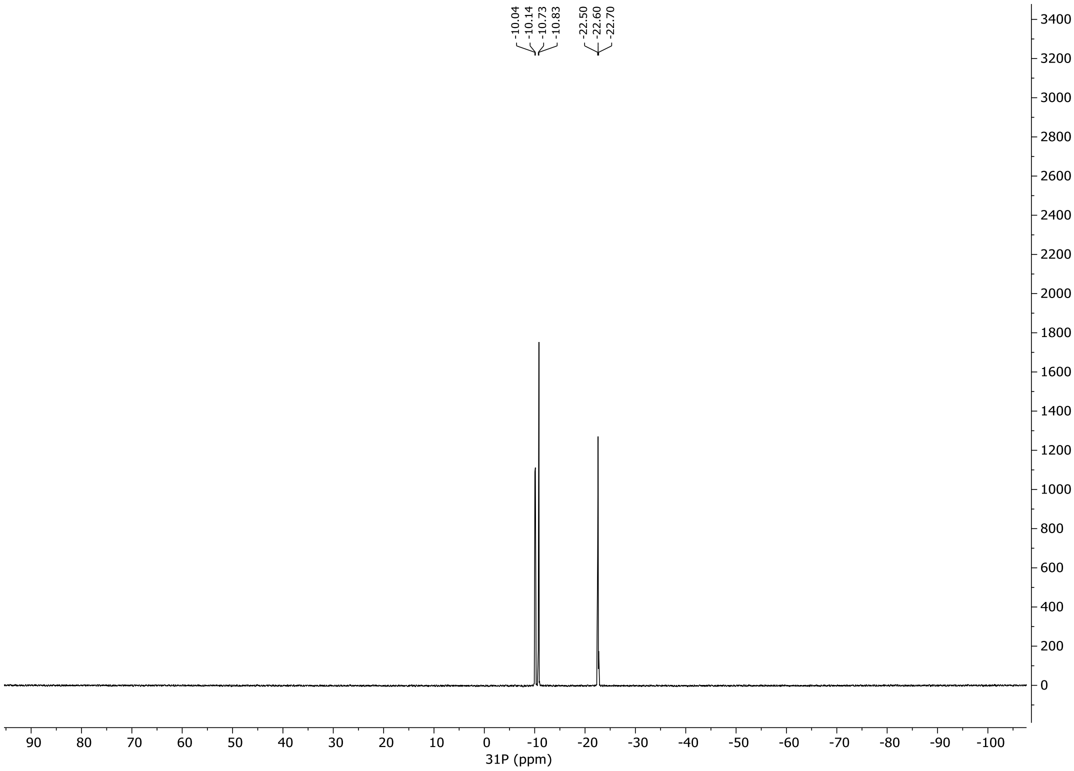

^{1}H, ^{13}C, $^{31}P\{^{1}H\}$ NMR spectra of **r^{NH2}ATP**

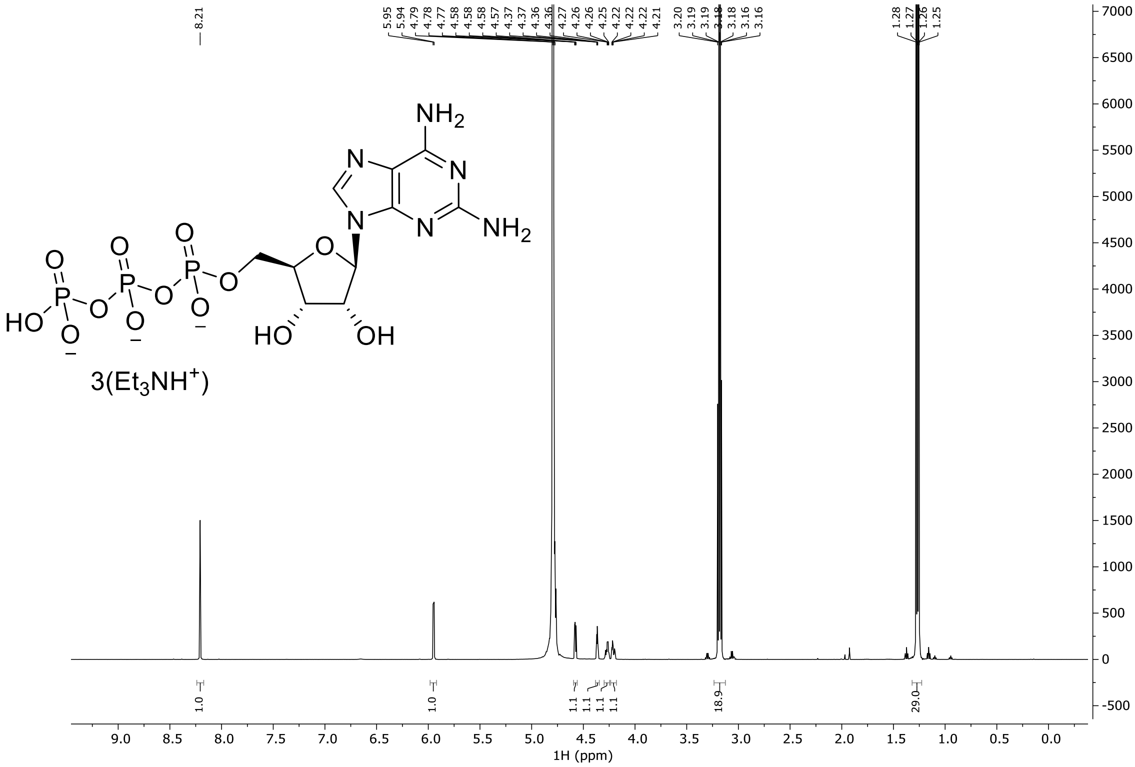

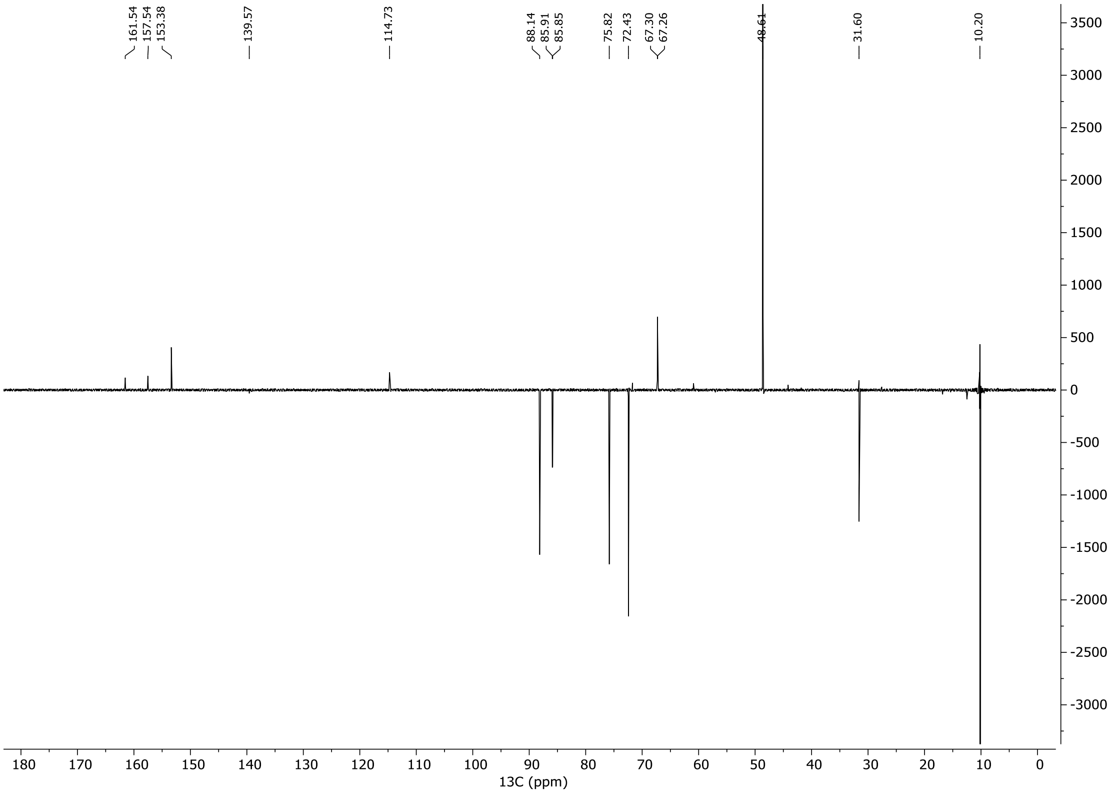
161.54
157.54
153.38
139.57
114.73
88.14
85.91
85.85
75.82
72.43
67.30
67.26
48.61
31.60
10.20
13C (ppm)

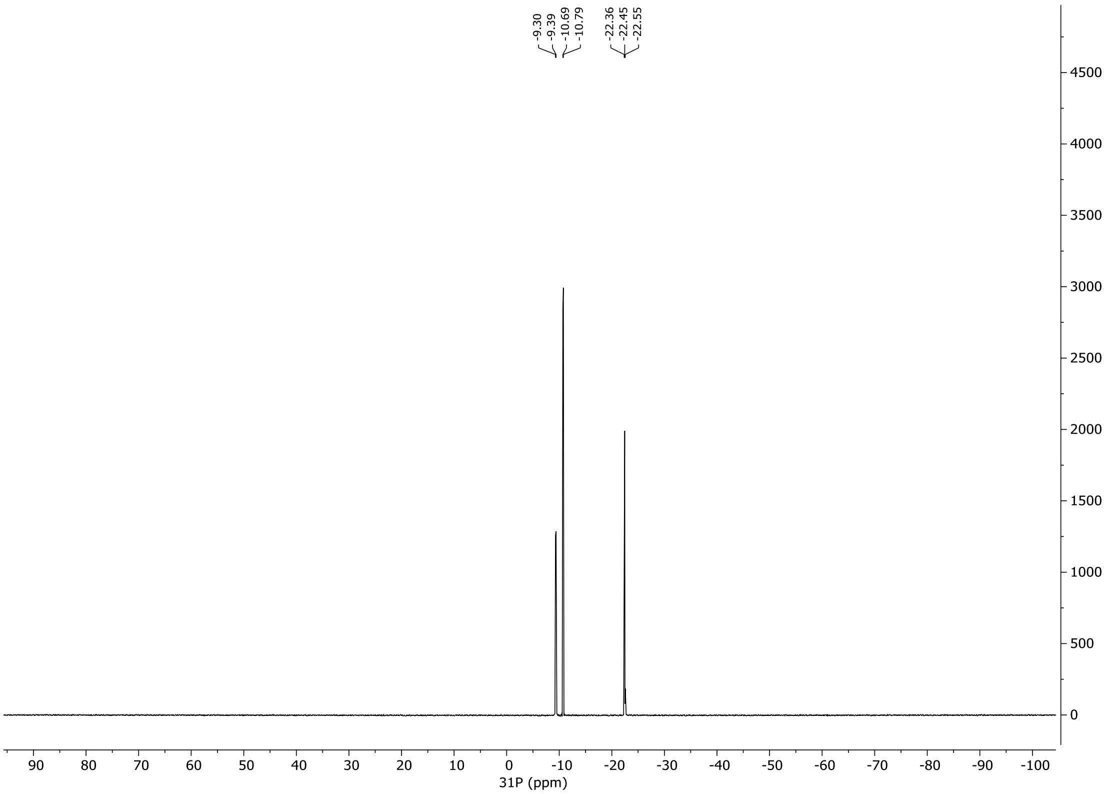
-9.30
-9.39
-10.69
-10.79
-22.36
-22.45
-22.55
31P (ppm)

^{1}H, ^{13}C, $^{31}P\{^{1}H\}$, ^{19}F NMR spectra of **r^F^ATP**

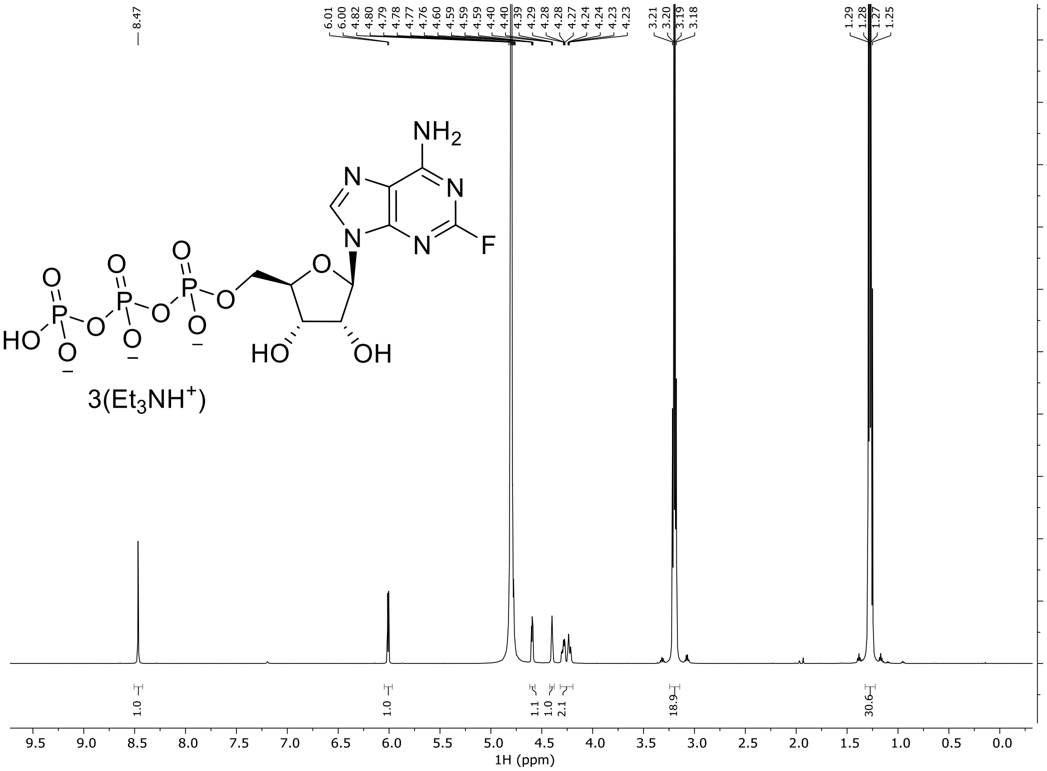

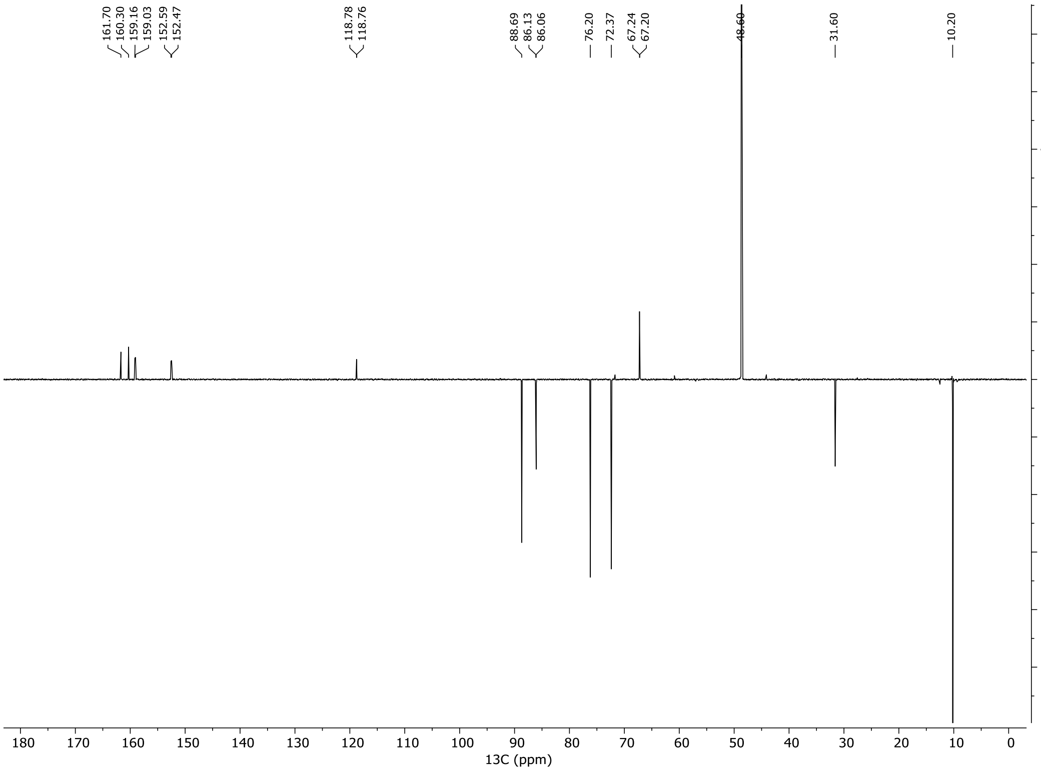

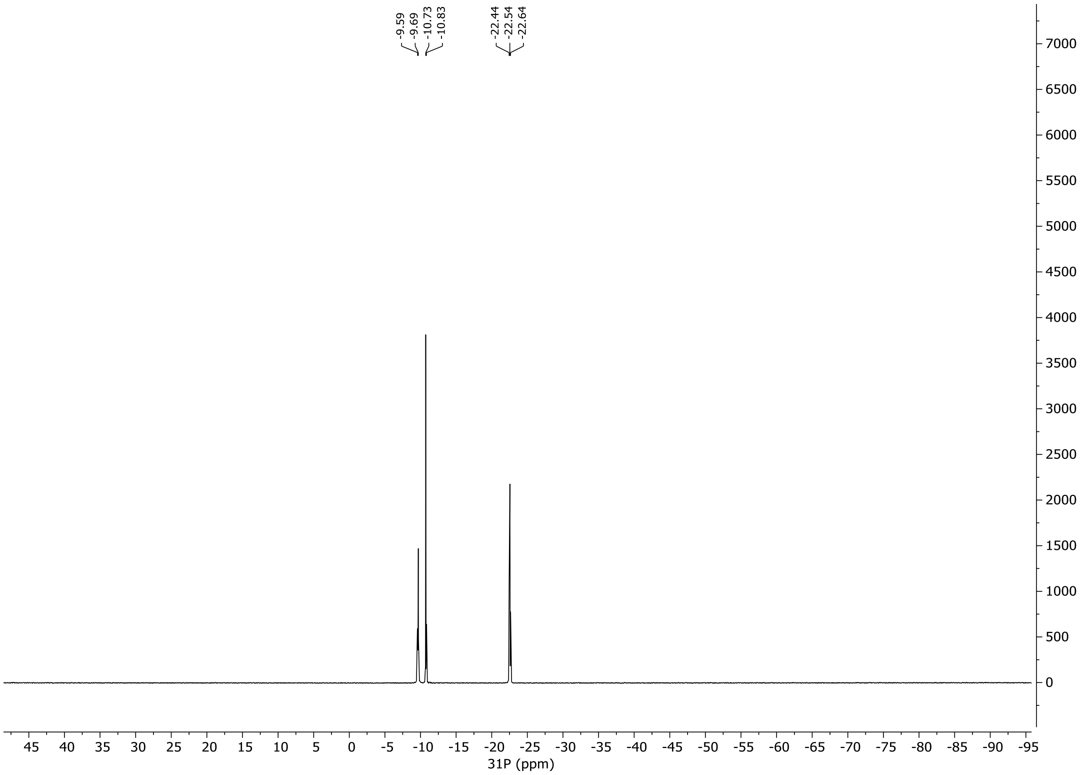
-9.59
-9.69
-10.73
-10.83
-22.44
-22.54
-22.64
31P (ppm)

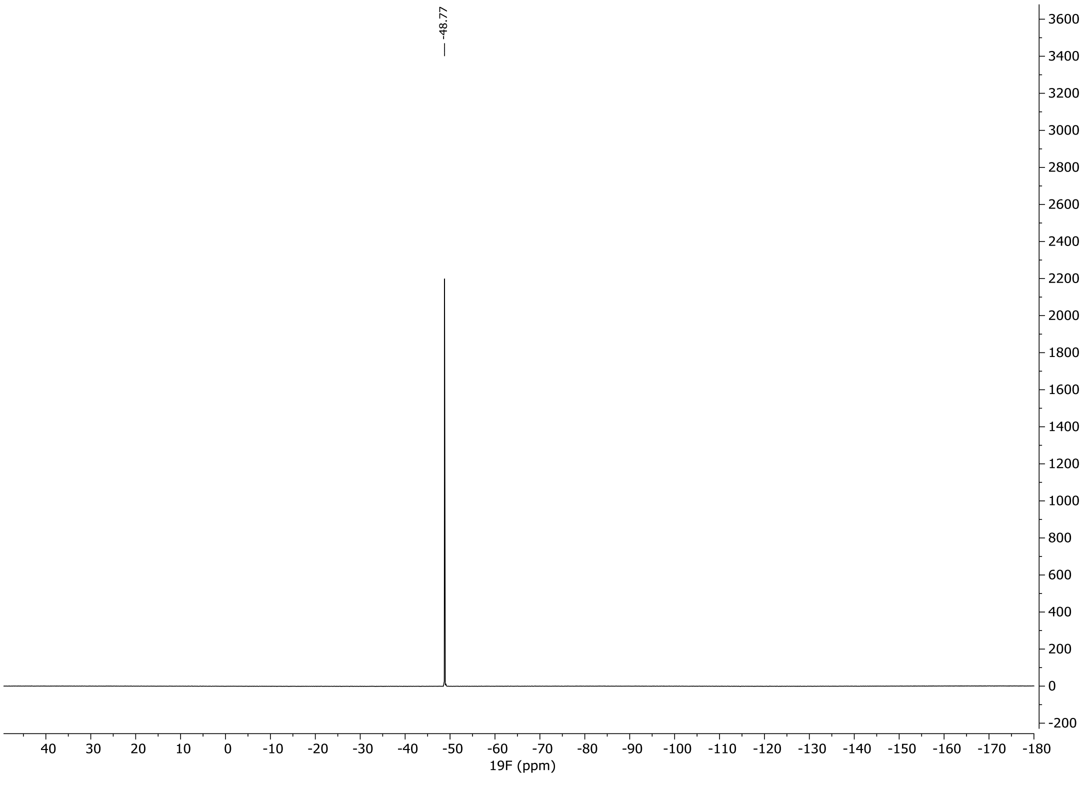
-48.77
19F (ppm)

^{1}H, ^{13}C, $^{31}P\{^{1}H\}$ NMR spectra of **r^{V}ATP**

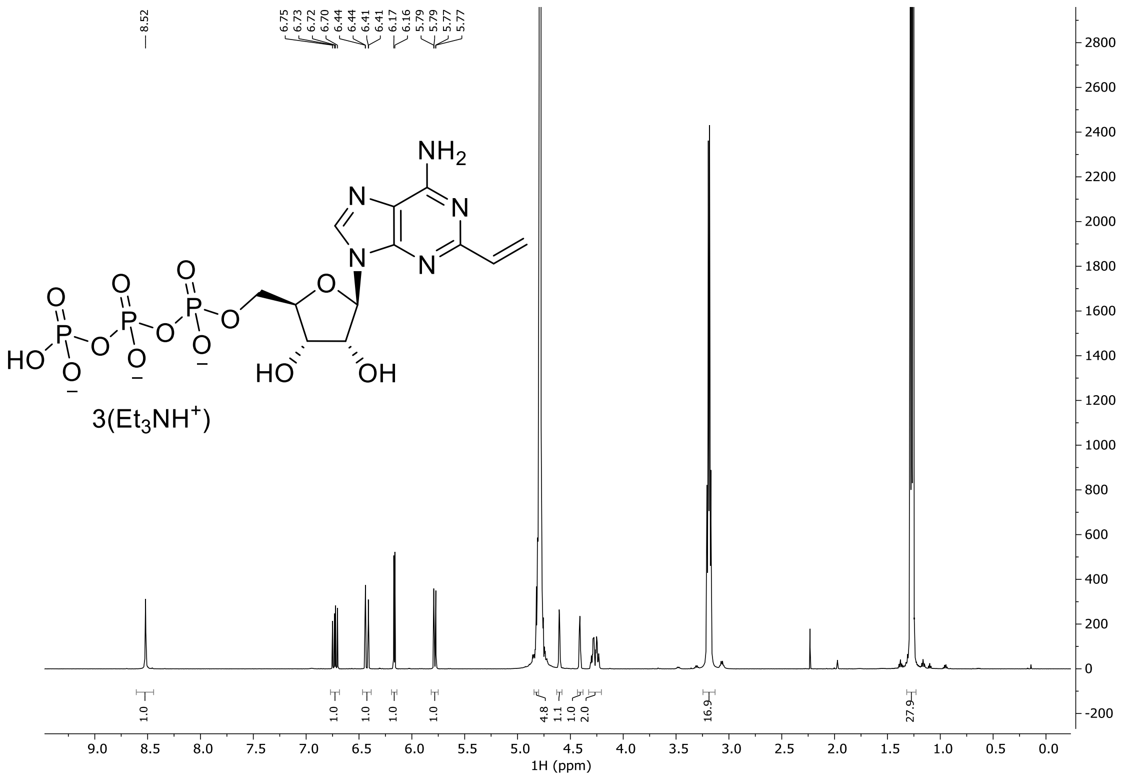

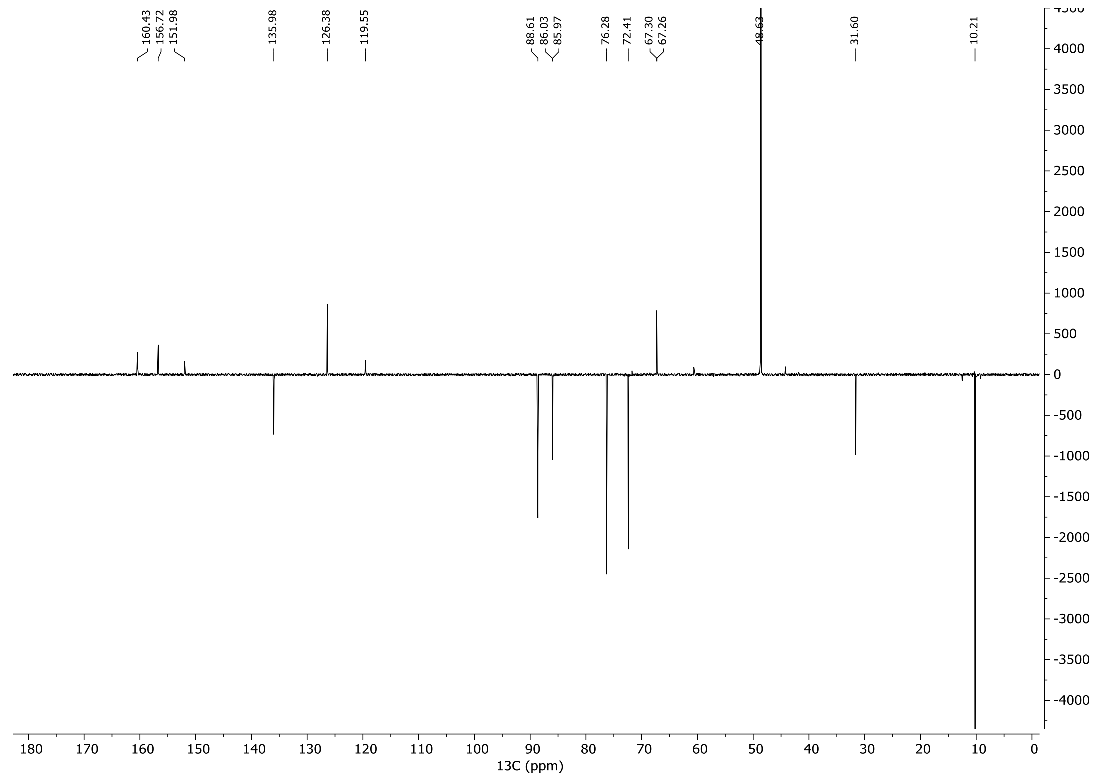

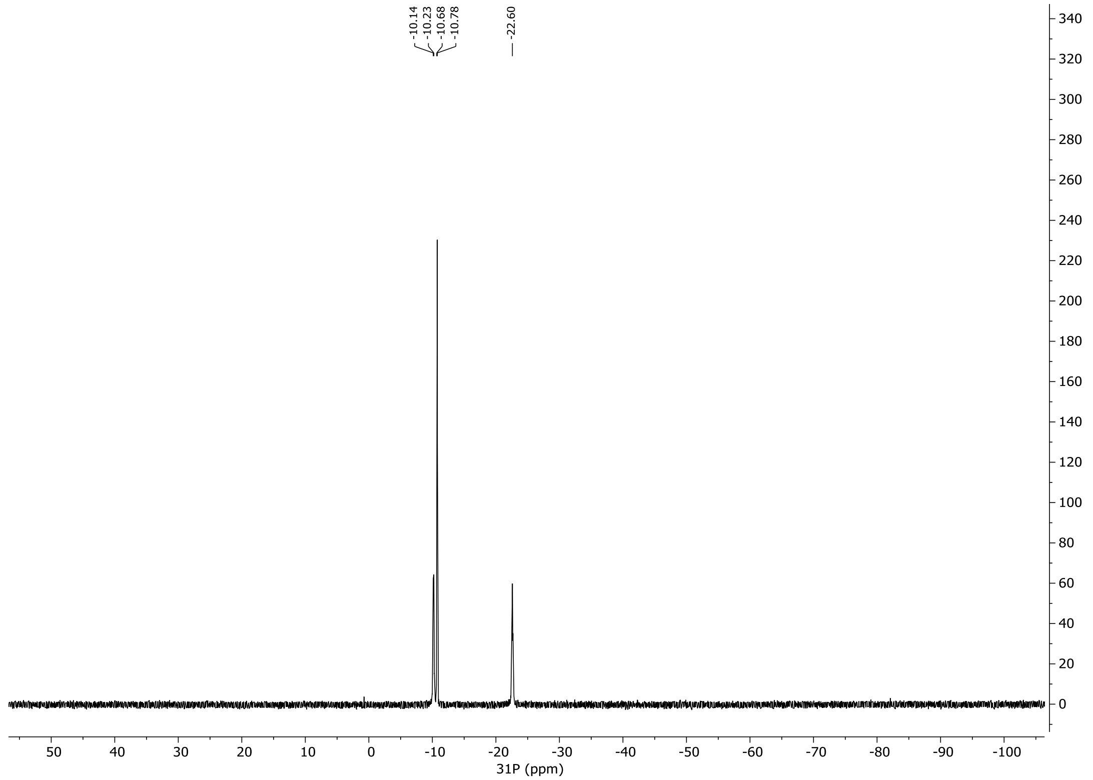

^{1}H, ^{13}C, ^{31}P{^{1}H} NMR spectra of **r^{Me}ATP**

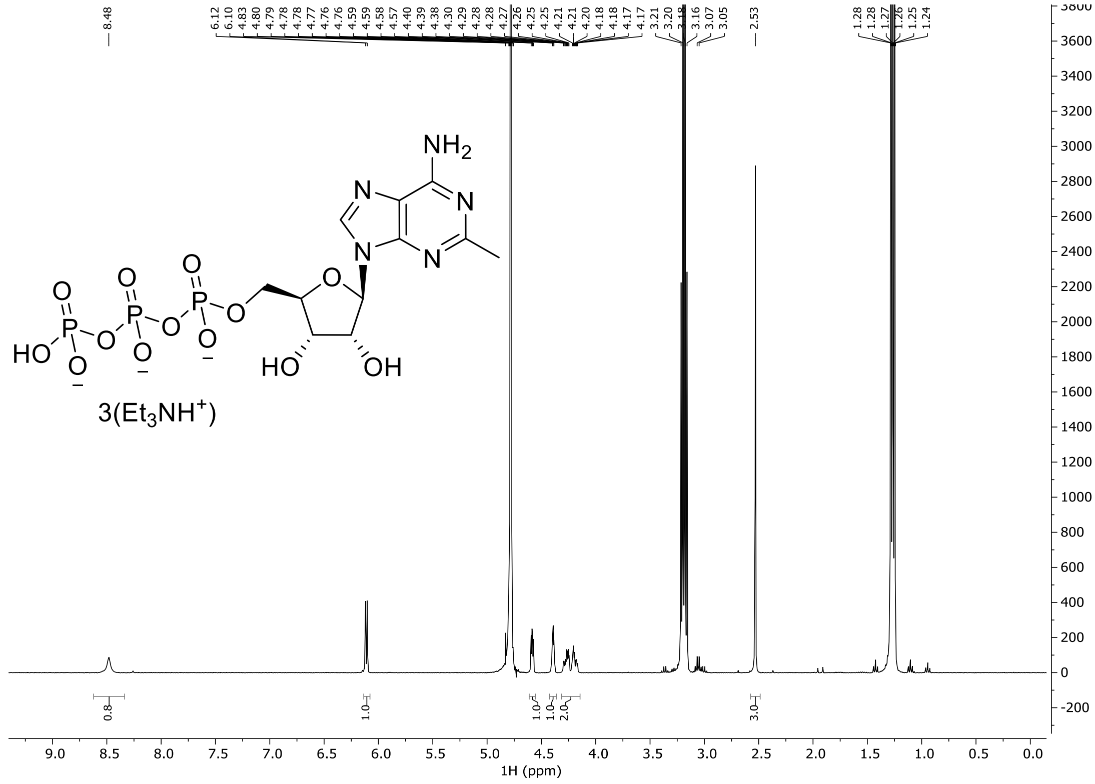

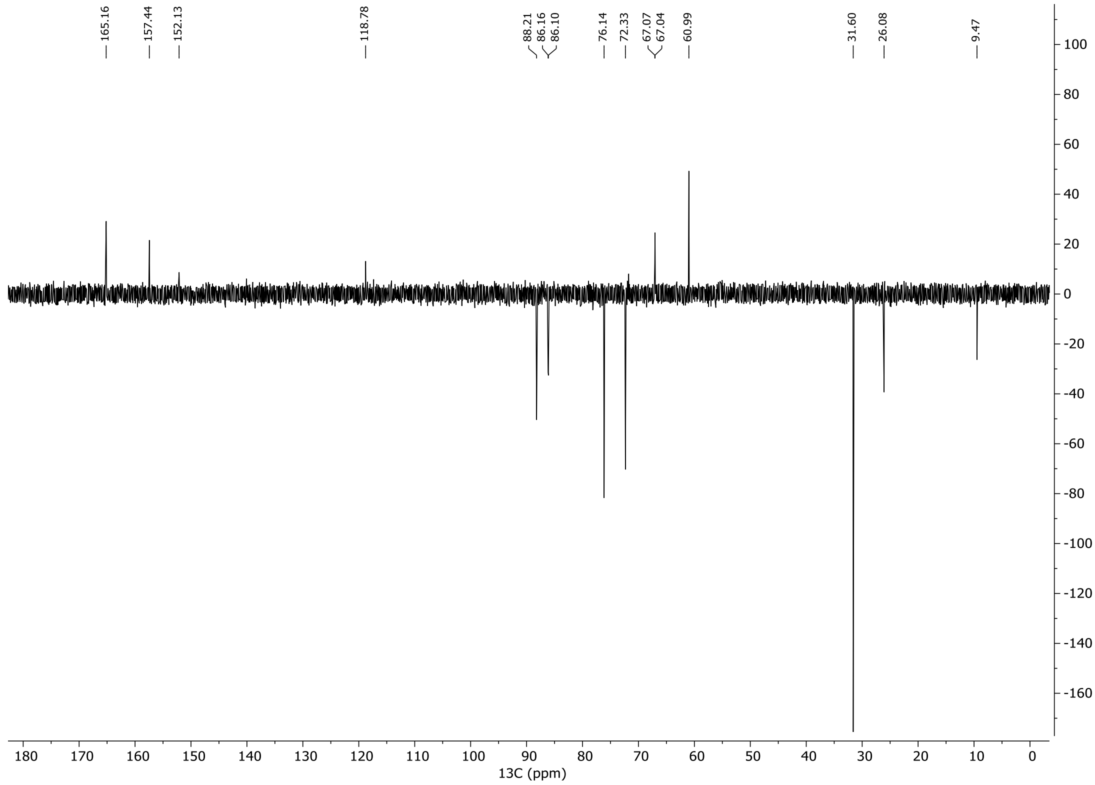
165.16
157.44
152.13
118.78
88.21
86.16
86.10
76.14
72.33
67.07
67.04
60.99
31.60
26.08
9.47
13C (ppm)

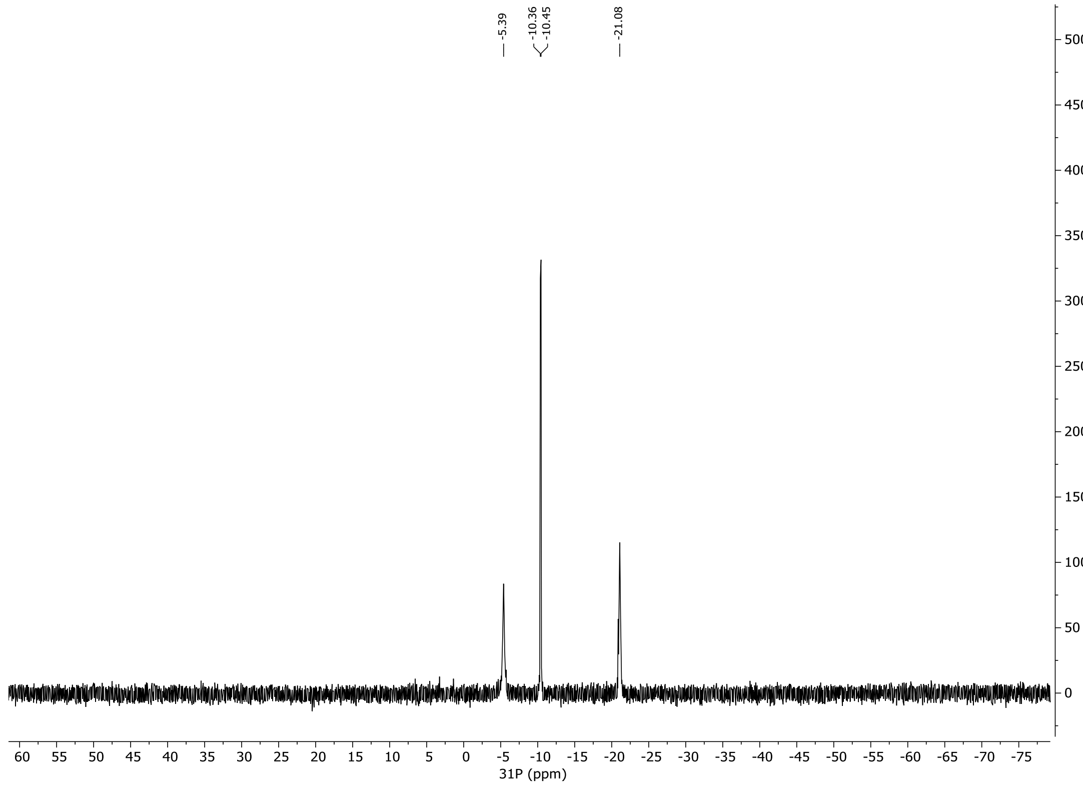
-5.39
-10.36
-10.45
-21.08
31P (ppm)

5. Copies of UPLC-MS chromatograms and spectra

70RNA_A

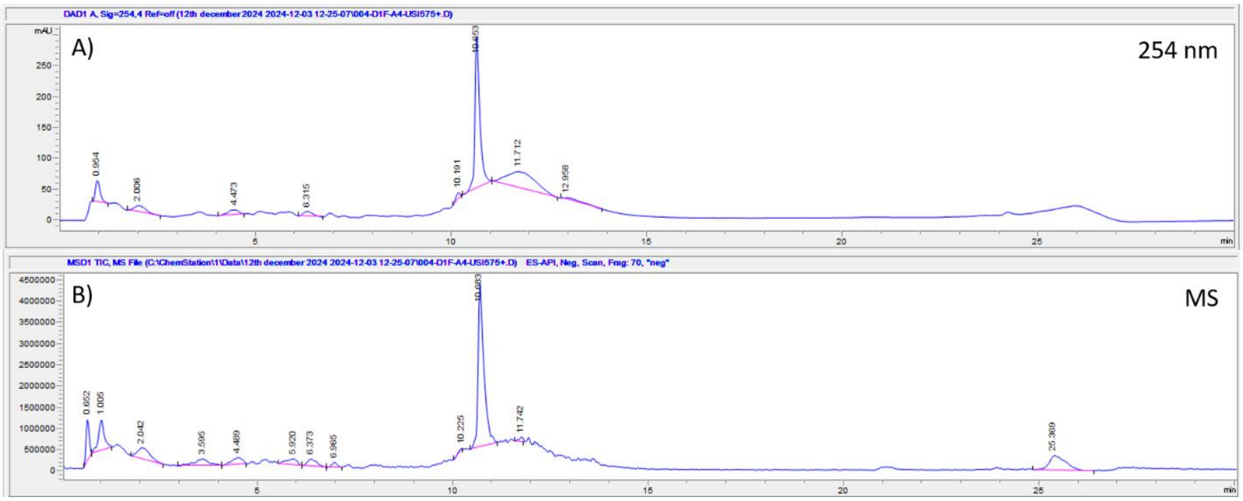

Figure S12. UPLC and MS chromatograms of **70RNA_A** after IVT reaction acquired at A) absorbance 254 nm; and B) MSD TIC.

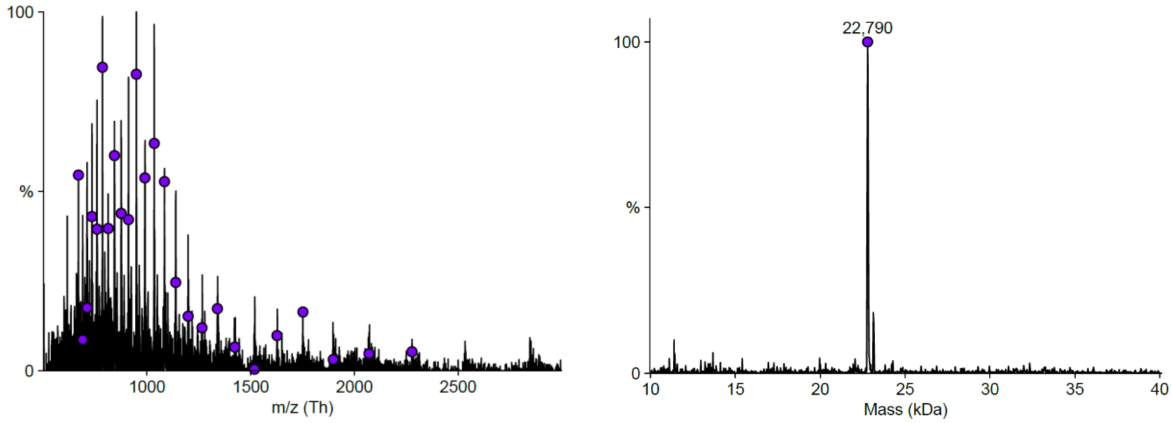

Figure S13. MS spectrum from UPLC-MS analysis of **70RNA_A** after IVT reaction. Left – raw spectrum, right - deconvoluted mass spectrum. Calculated mass: 22763 Da; found mass: 22790 Da (product + Na^+).

70RNA_^{Cl}A

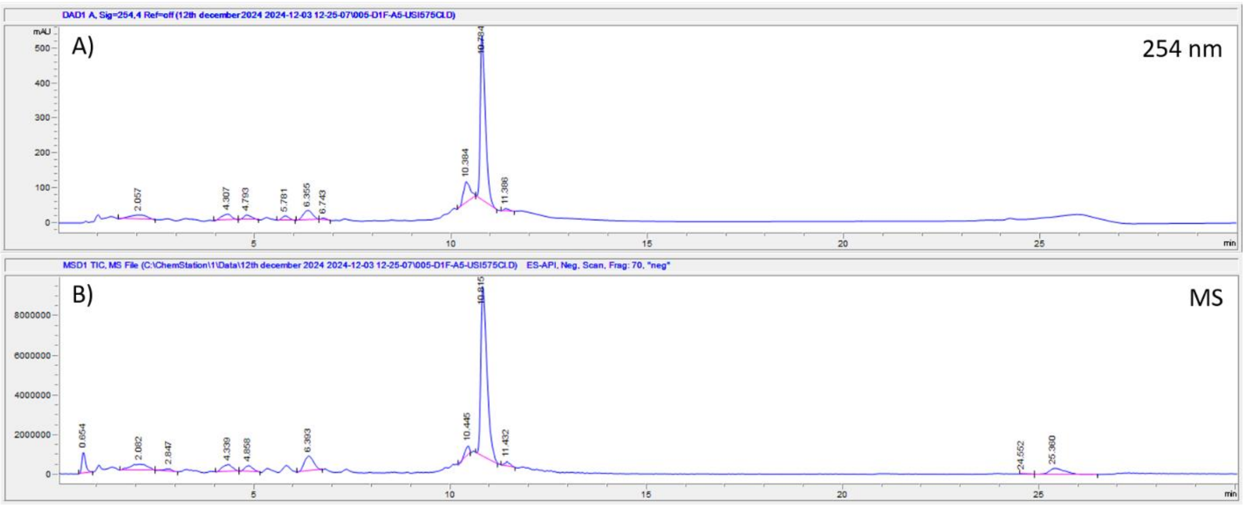

Figure S14. UPLC and MS chromatograms of **70RNA_^{Cl}A** after IVT reaction acquired at A) absorbance 254 nm; and B) MSD TIC.

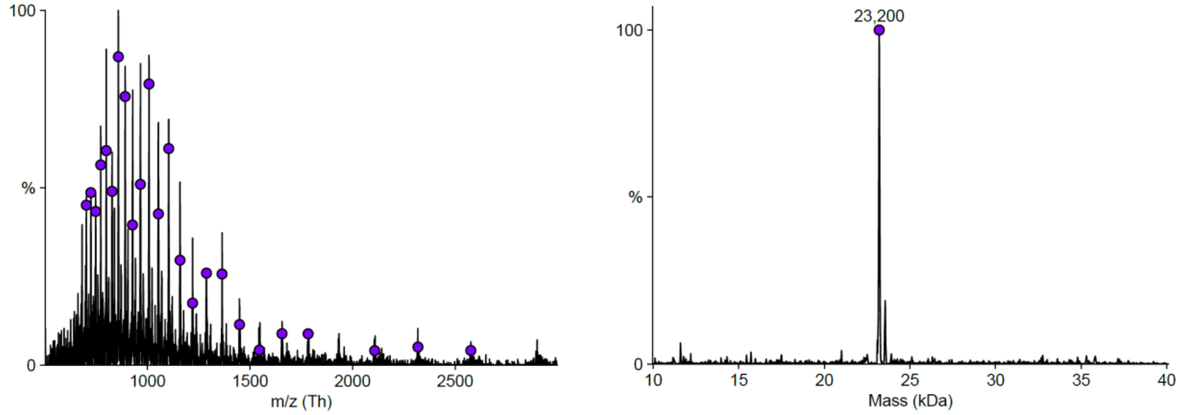

Figure S15. MS spectrum from UPLC-MS analysis of **70RNA_^{Cl}A** after IVT reaction. Left – raw spectrum, right - deconvoluted mass spectrum. Calculated mass: 23176 Da; found mass: 23200 Da (product + Na^+).

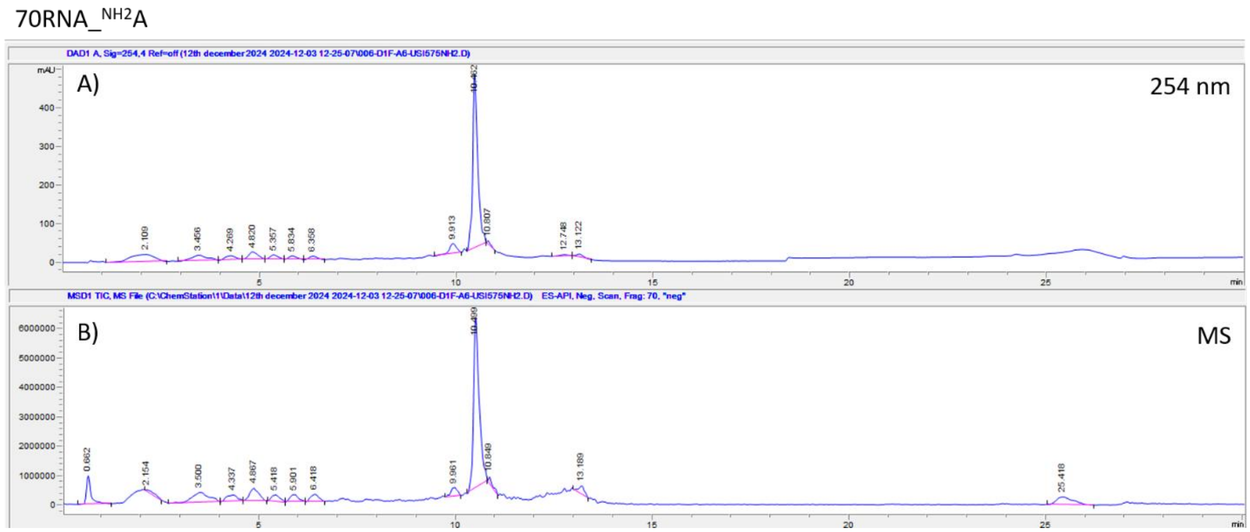

Figure S16. UPLC and MS chromatograms of **70RNA_ ^{NH2}A** after IVT reaction acquired at A) absorbance 254 nm; and B) MSD TIC.

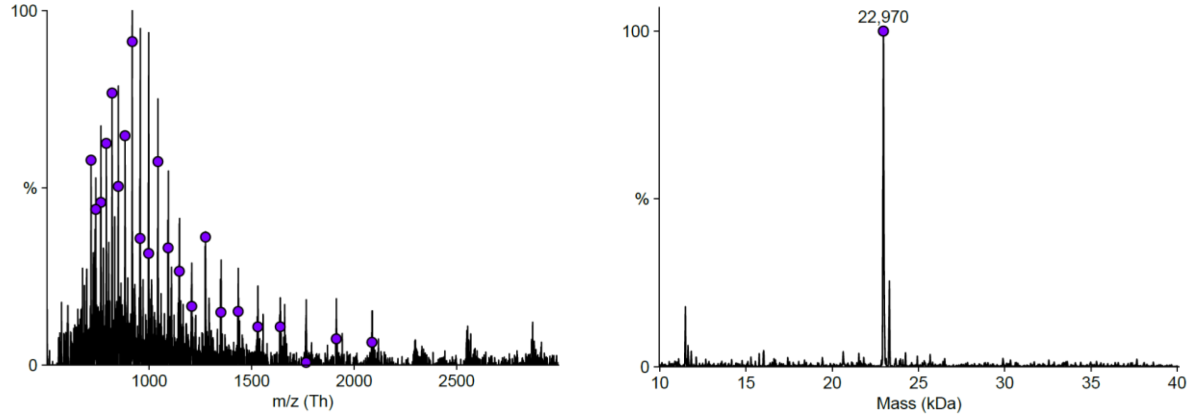

Figure S17. MS spectrum from UPLC-MS analysis of **70RNA_ ^{NH2}A** after IVT reaction. Left – raw spectrum, right - deconvoluted mass spectrum. Calculated mass: 22943 Da; found mass: 22970 Da (product + Na^+).

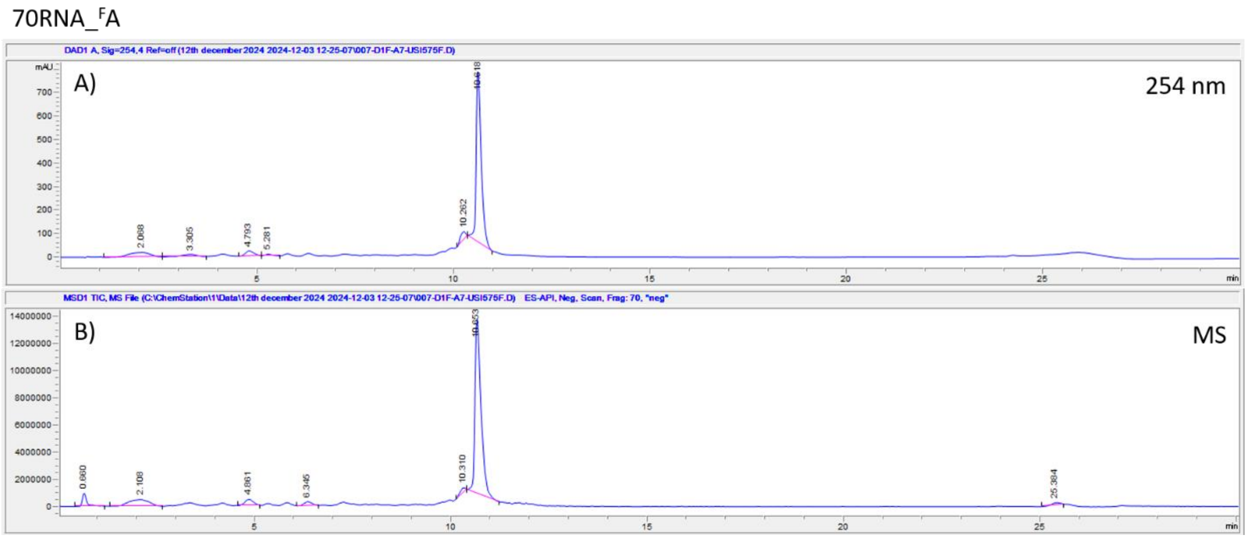

Figure S18. UPLC and MS chromatograms of **70RNA_^{F}A** after IVT reaction acquired at A) absorbance 254 nm; and B) MSD TIC.

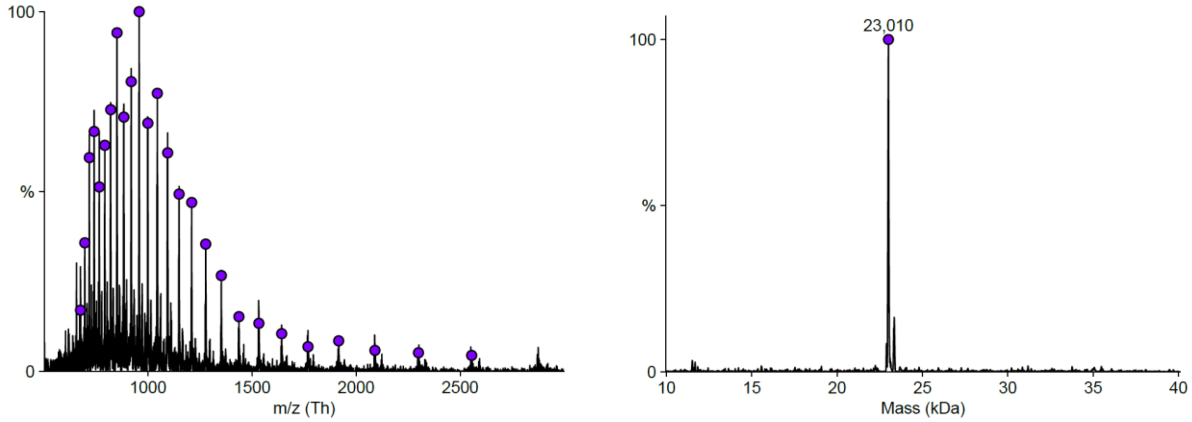

Figure S19. MS spectrum from UPLC-MS analysis of **70RNA_^{F}A** after IVT reaction. Left – raw spectrum, right - deconvoluted mass spectrum. Calculated mass: 22979 Da; found mass: 23010 Da (product + Na^{+}).

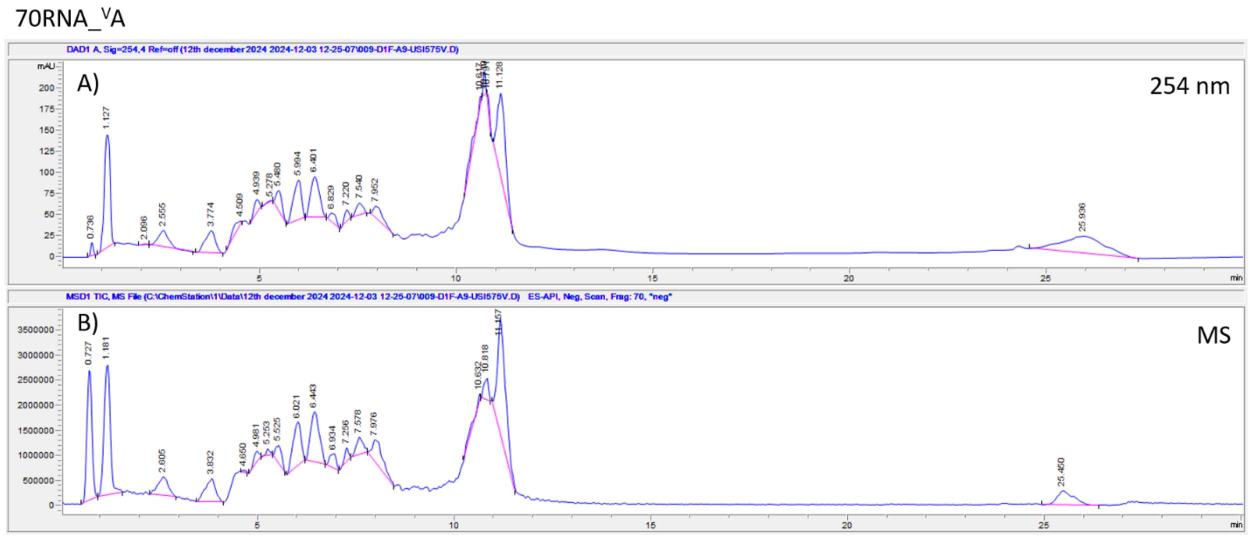

Figure S20. UPLC and MS chromatograms of **70RNA_^{V}A** after IVT reaction acquired at A) absorbance 254 nm; and B) MSD TIC.

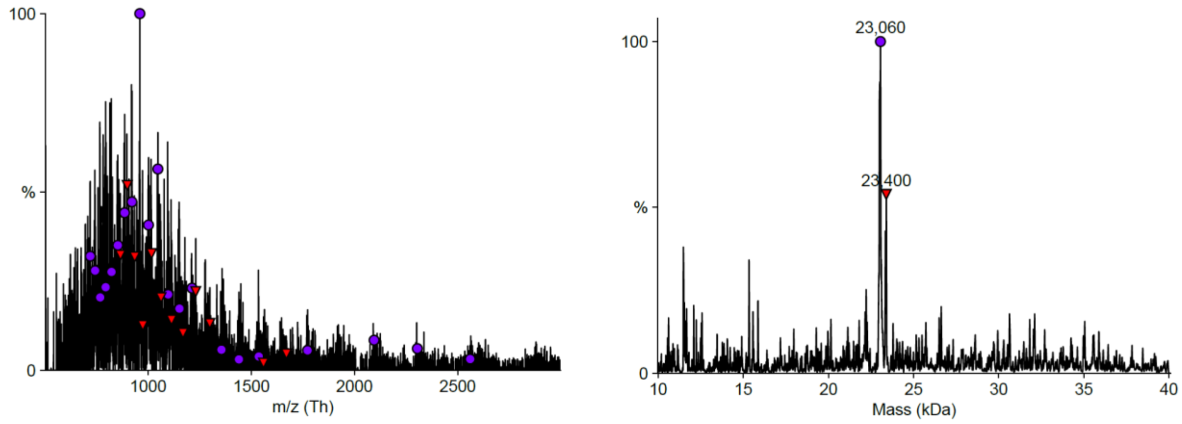

Figure S21. MS spectrum from UPLC-MS analysis of PEX reaction of **70RNA_^{V}A**. Left – raw spectrum, right - deconvoluted mass spectrum. Calculated mass: 23075 Da; found mass: 23060 Da and 23400 Da (product + rAMP).

70RNA_^{E}A

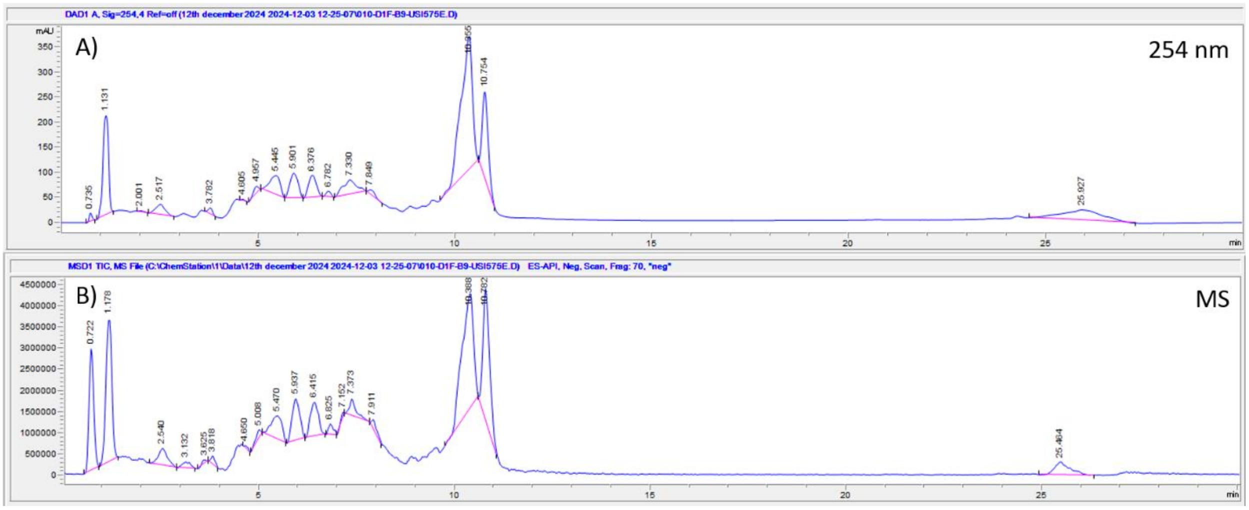

Figure S22. UPLC and MS chromatograms of **70RNA_^{E}A** after IVT reaction acquired at A) absorbance 254 nm; and B) MSD TIC.

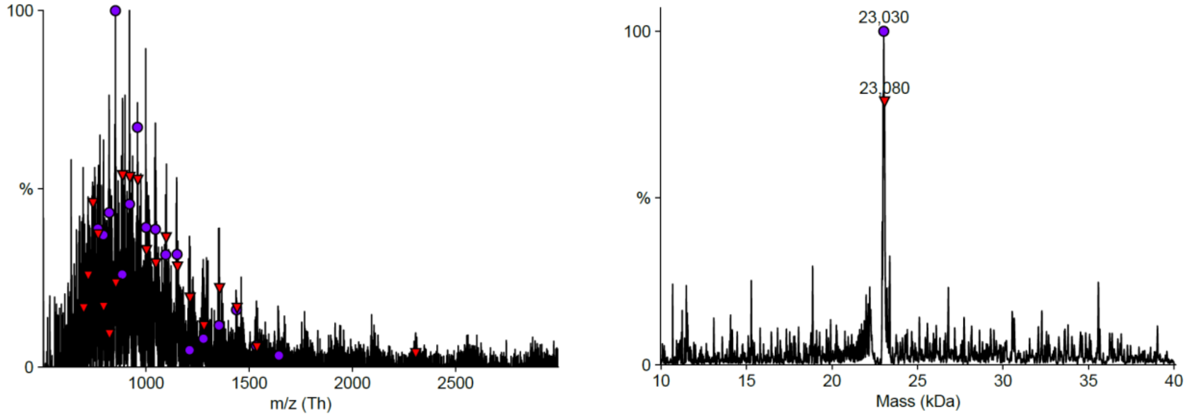

Figure S23. MS spectrum from UPLC-MS analysis of **70RNA_^{E}A** after IVT reaction. Left – raw spectrum, right - deconvoluted mass spectrum. Calculated mass: 23051 Da; found mass: 23030 Da and 23080 Da (product + Na^{+}).

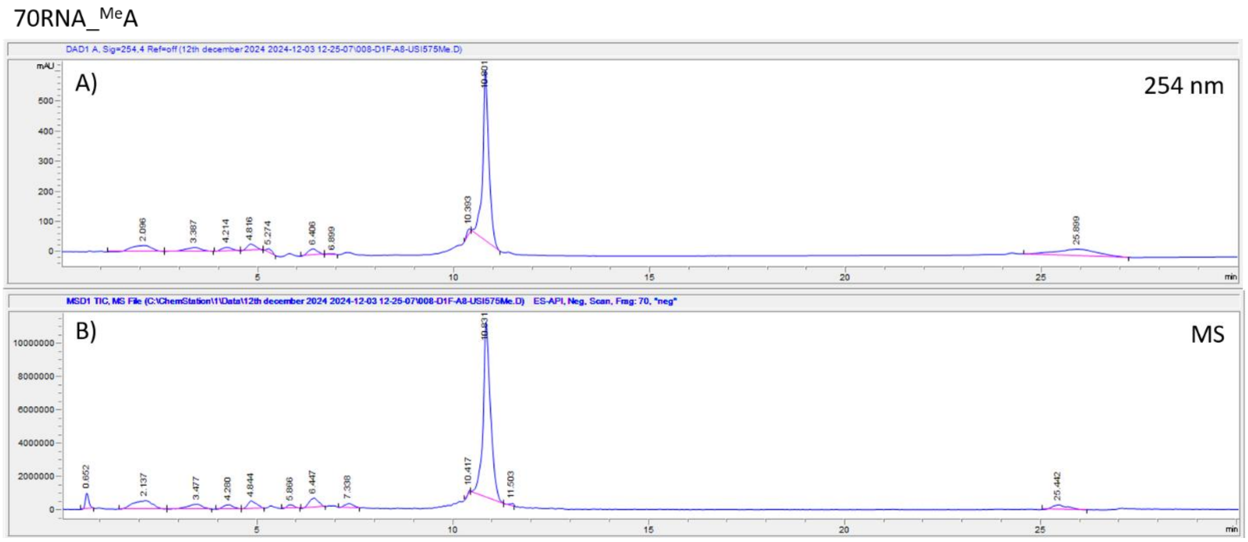

Figure S24. UPLC and MS chromatograms of **70RNA_^{Me}A** after IVT reaction acquired at A) absorbance 254 nm; and B) MSD TIC.

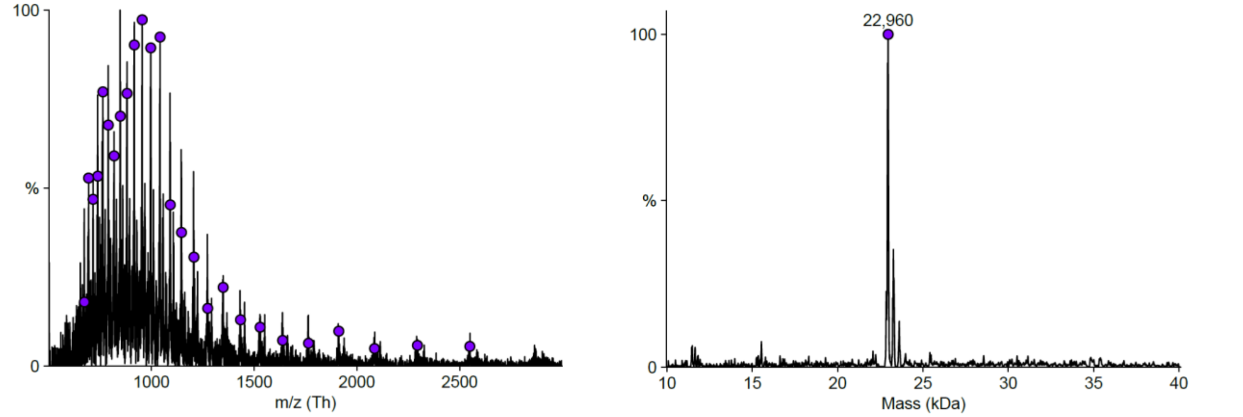

Figure S25. MS spectrum from UPLC-MS analysis of **70RNA_^{Me}A** after IVT reaction. Left – raw spectrum, right - deconvoluted mass spectrum. Calculated mass: 22931 Da; found mass: 22960 Da (product + Na^+).

35RNA

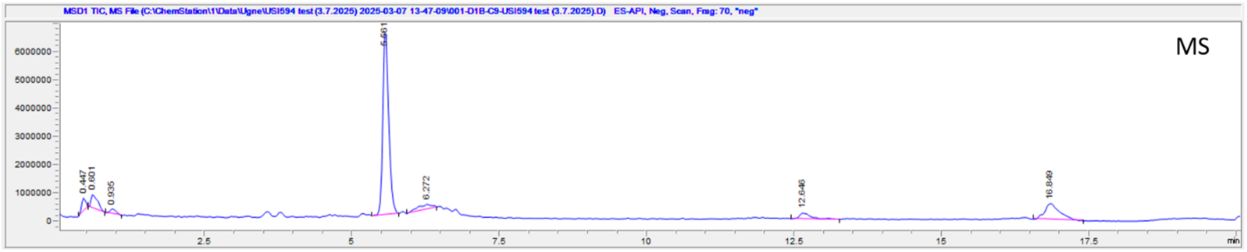

Figure S26. MS chromatogram of **35RNA** after IVT reaction acquired at MSD TIC.

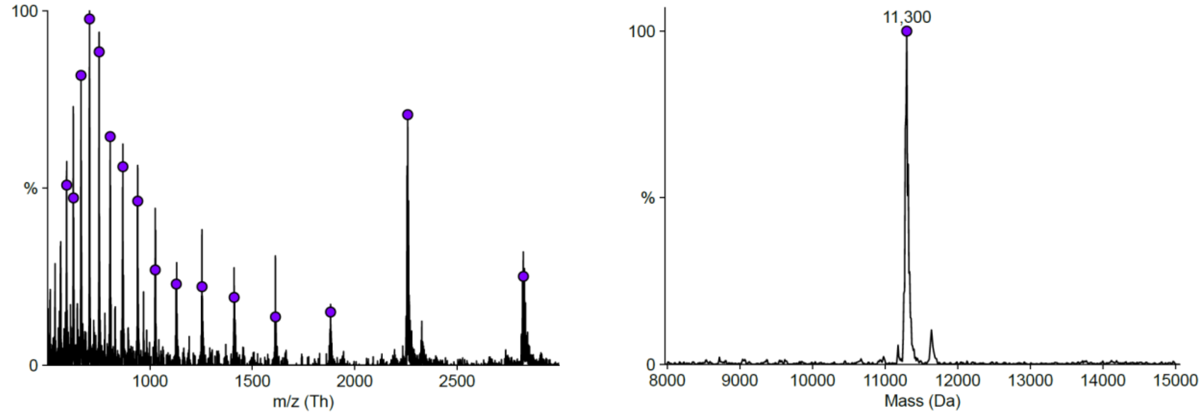

Figure S27. MS spectrum from UPLC-MS analysis of **35RNA** after IVT reaction. Left – raw spectrum, right - deconvoluted mass spectrum. Calculated mass: 11261 Da; found mass: 11300 Da (product + K^+).

31RNA_A

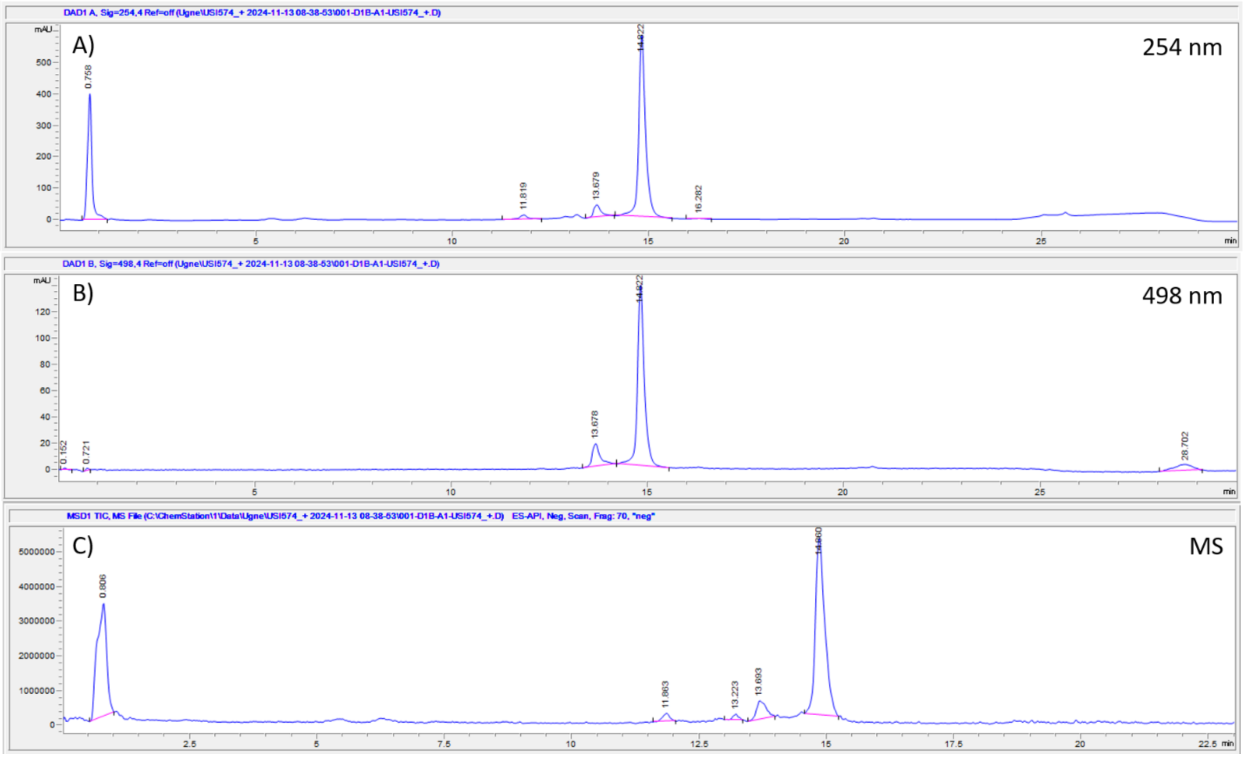

Figure S28. UPLC and MS chromatograms of **31RNA_A** after PEX reaction acquired at A) absorbance 254 nm; B) absorbance 498 nm (FAM); and C) MSD TIC.

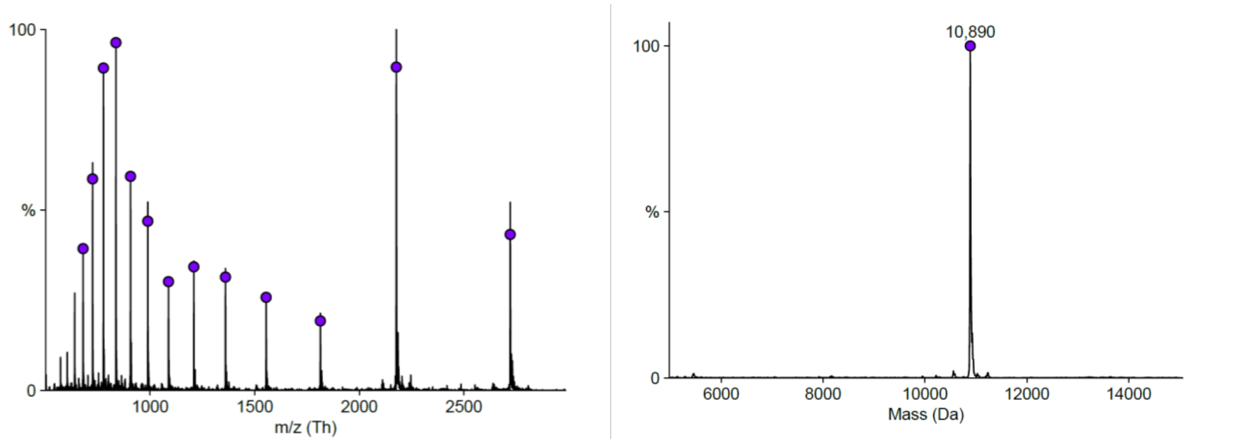

Figure S29. MS spectrum from UPLC-MS analysis of **31RNA_A** after PEX reaction. Left – raw spectrum, right - deconvoluted mass spectrum. Calculated mass: 10566 Da; found mass: 10890 Da (product + rAMP).

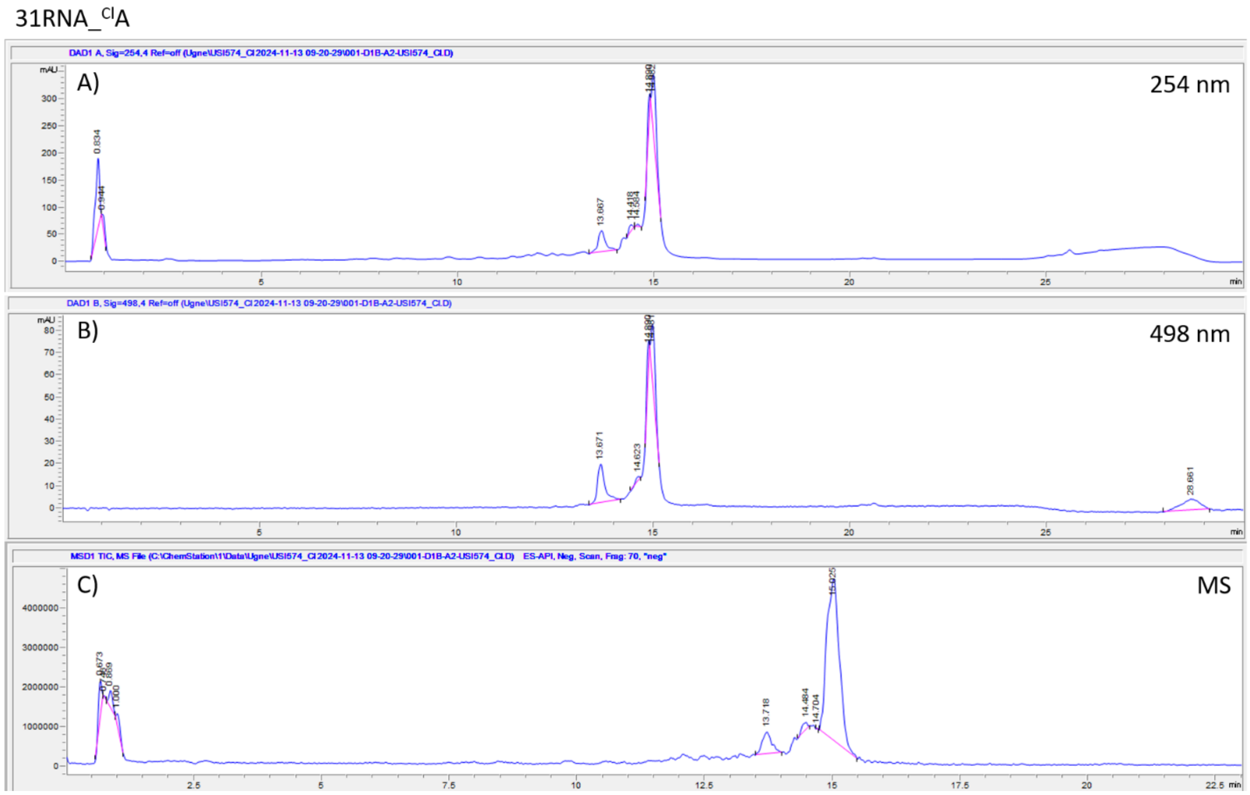

Figure S30. UPLC and MS chromatograms of **31RNA_^{Cl}A** after PEX reaction acquired at A) absorbance 254 nm; B) absorbance 498 nm (FAM); and C) MSD TIC.

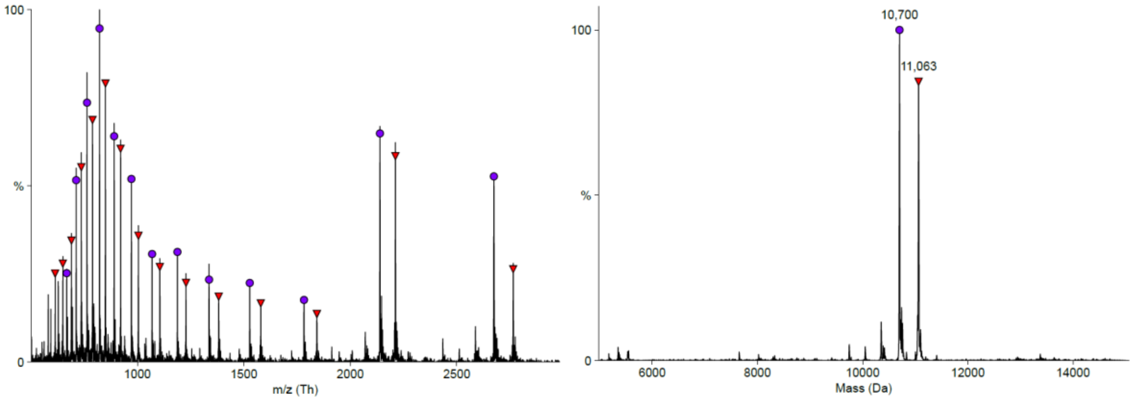

Figure S31. MS spectrum from UPLC-MS analysis of **31RNA_^{Cl}A** after PEX reaction. Left – raw spectrum, right - deconvoluted mass spectrum. Calculated mass: 10704 Da; found mass: 10700 Da and 11063 Da (product + rGMP).

31RNA_^{NH2}A

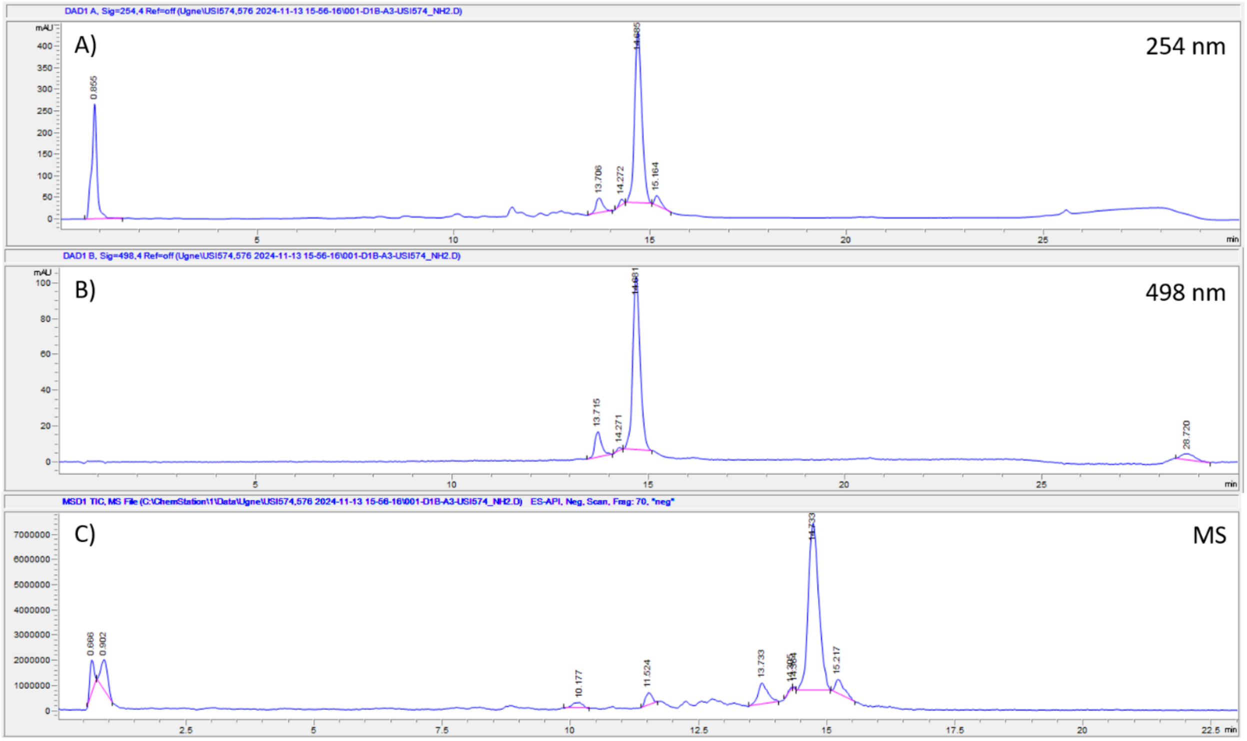

Figure S32. UPLC and MS chromatograms of **31RNA_^{NH2}A** after PEX reaction acquired at A) absorbance 254 nm; B) absorbance 498 nm (FAM); and C) MSD TIC.

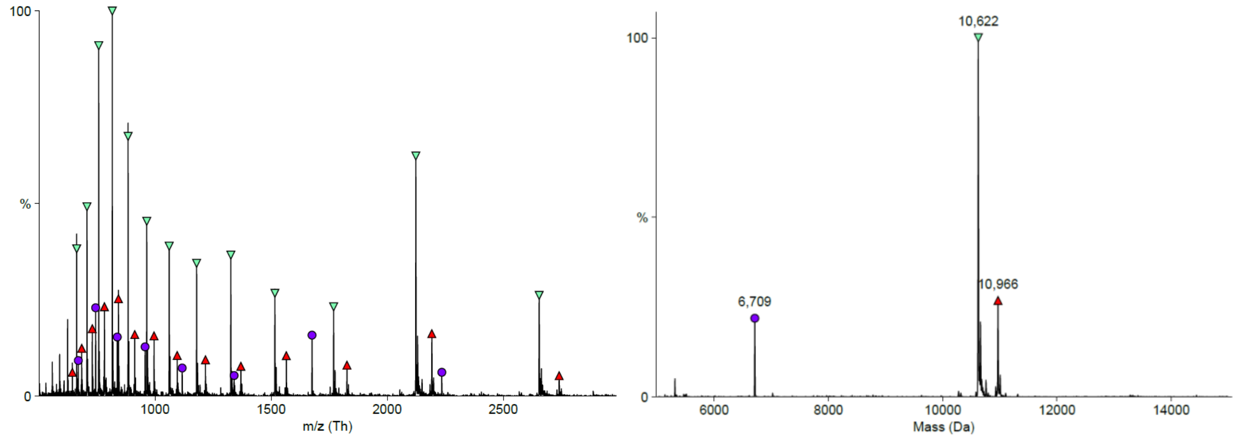

Figure S33. MS spectrum from UPLC-MS analysis of **31RNA_^{NH2}A** after PEX reaction. Left – raw spectrum, right - deconvoluted mass spectrum. Calculated mass: 10626 Da; found mass: 10622 Da and 10966 Da (product + rAMP).

31RNA_FA

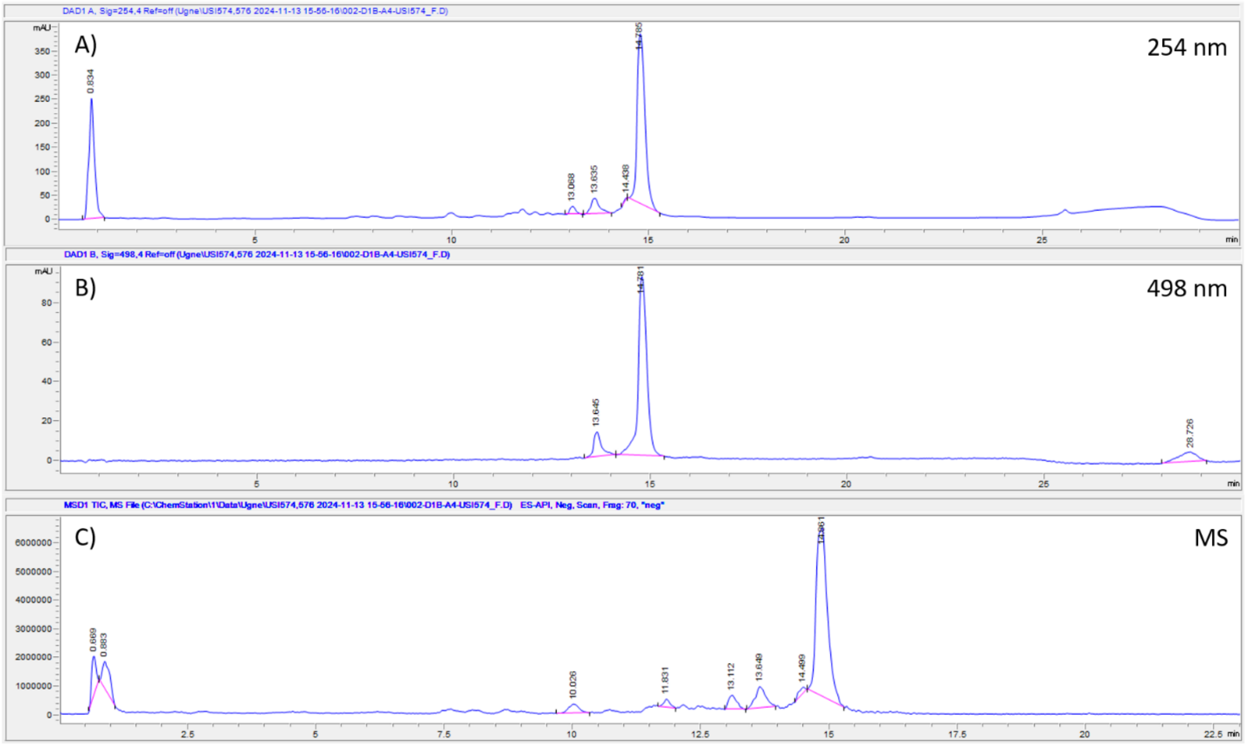

Figure S34. UPLC and MS chromatograms of **31RNA_^{F}A** after PEX reaction acquired at A) absorbance 254 nm; B) absorbance 498 nm (FAM); and C) MSD TIC.

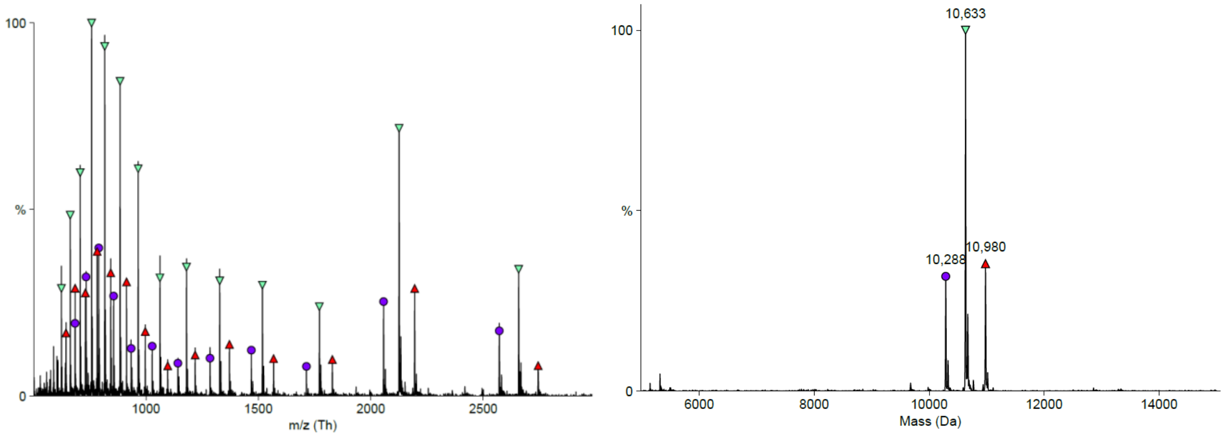

Figure S35. MS spectrum from UPLC-MS analysis of **31RNA_^{F}A** after PEX reaction. Left – raw spectrum, right - deconvoluted mass spectrum. Calculated mass: 10638 Da; found mass: 10633 Da, 10980 Da (product + rAMP) and 10288 Da (product - rGMP).

.

31RNA_^{Me}A

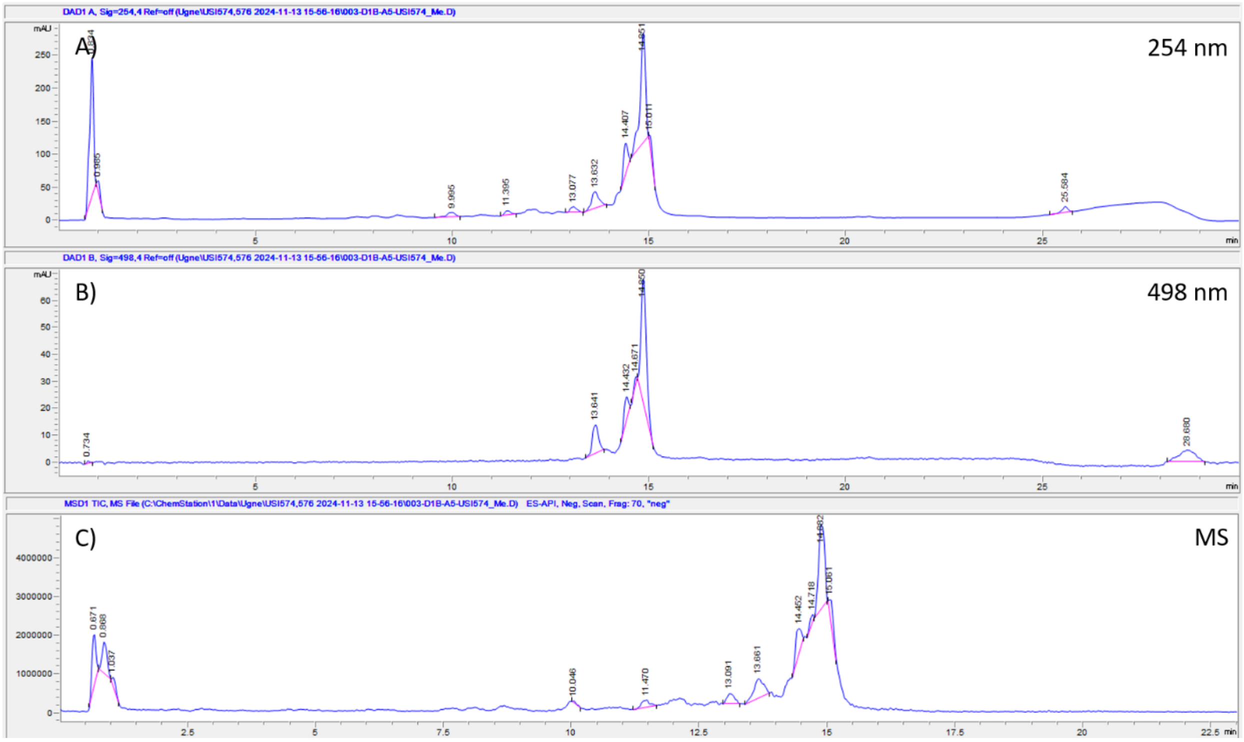

Figure S36. UPLC and MS chromatograms of **31RNA_^{Me}A** after PEX reaction acquired at A) absorbance 254 nm; B) absorbance 498 nm (FAM); and C) MSD TIC.

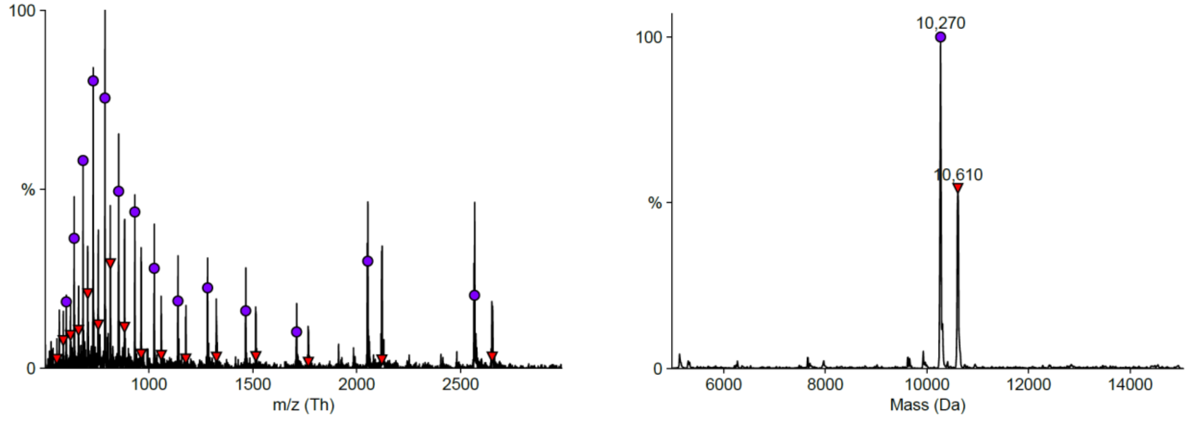

Figure S37. MS spectrum from UPLC-MS analysis of **31RNA_^{Me}A** after PEX. Left – raw spectrum, right - deconvoluted mass spectrum. Calculated mass: 10622 Da; found mass: 10610 Da and 10270 Da (product – rGMP).

16RNA_SNI_^{V}A

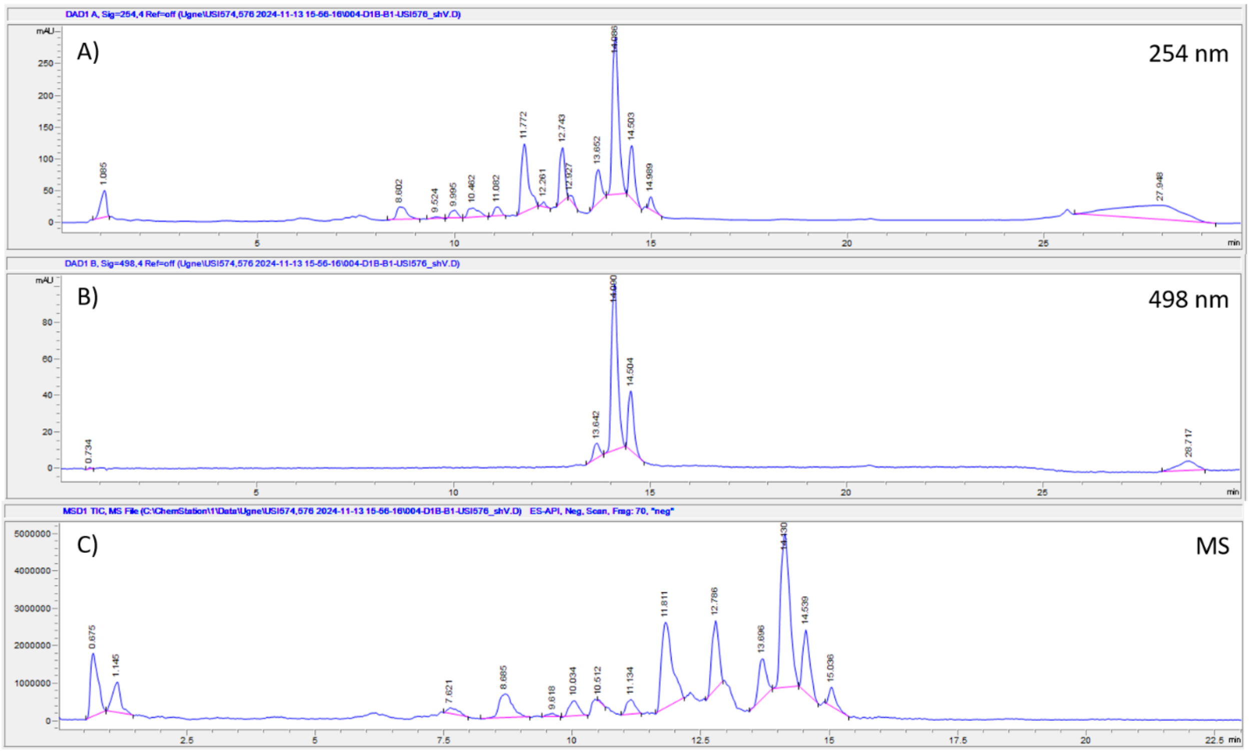

Figure S38. UPLC and MS chromatograms of **16RNA_SNI_^{V}A** after PEX reaction acquired at A) absorbance 254 nm; B) absorbance 498 nm (FAM); and C) MSD TIC.

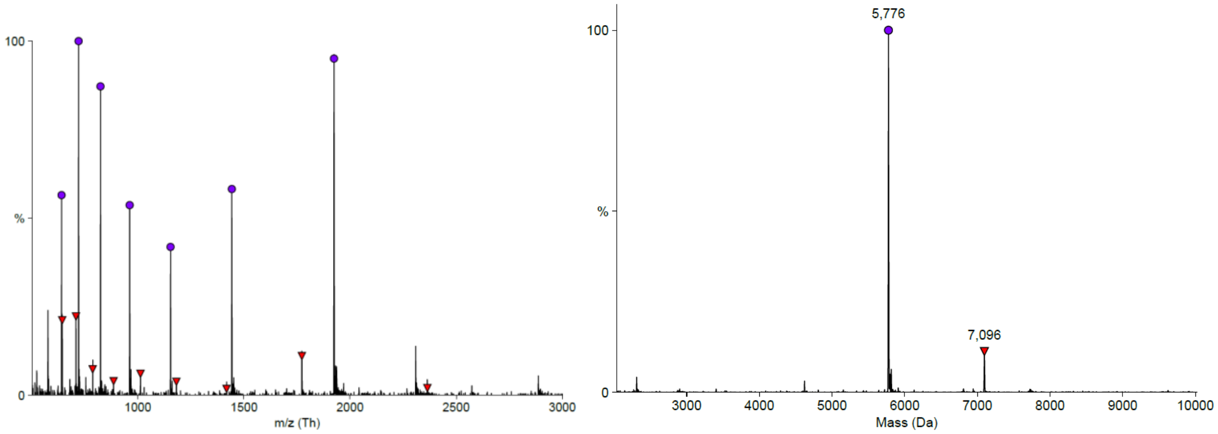

Figure S39. MS spectrum from UPLC-MS analysis of **16RNA_SNI_^{V}A** after PEX reaction. Left – raw spectrum, right - deconvoluted mass spectrum. Calculated mass: 5778 Da; found mass: 5776 Da.

16RNA_SNI_^{E}A

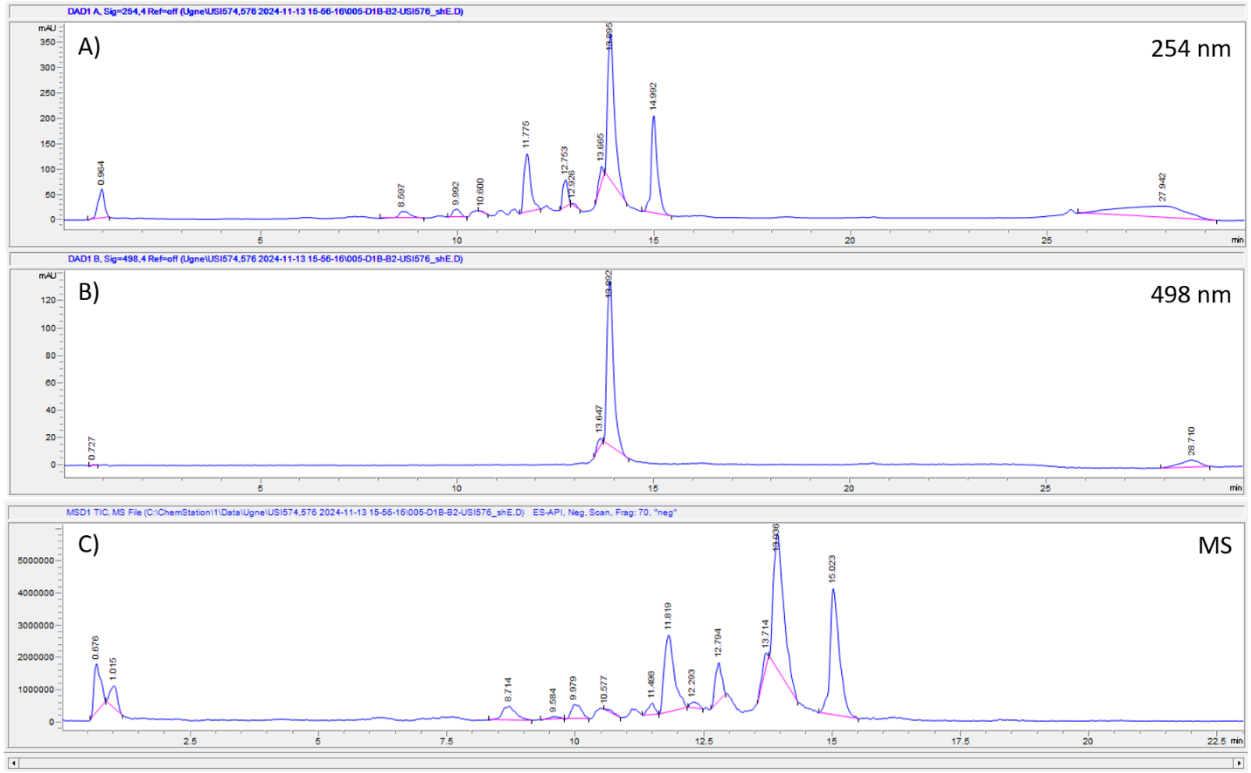

Figure S40. UPLC and MS chromatograms of **16RNA_SNI_^{E}A** after PEX reaction acquired at A) absorbance 254 nm; B) absorbance 498 nm (FAM); and C) MSD TIC.

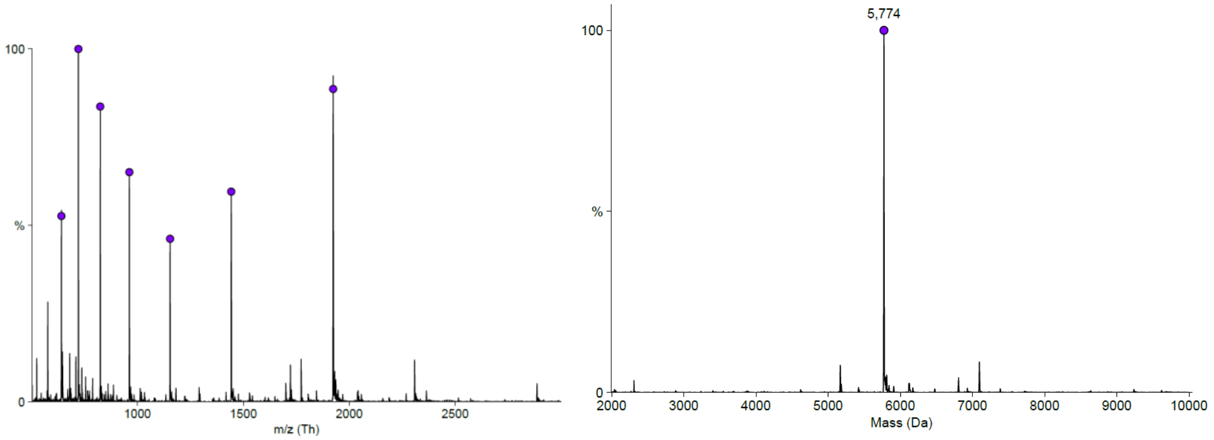

Figure S41. MS spectrum from UPLC-MS analysis **16RNA_SNI_^{E}A** after PEX reaction. Left – raw spectrum, right - deconvoluted mass spectrum. Calculated mass: 5776 Da; found mass: 5774 Da.

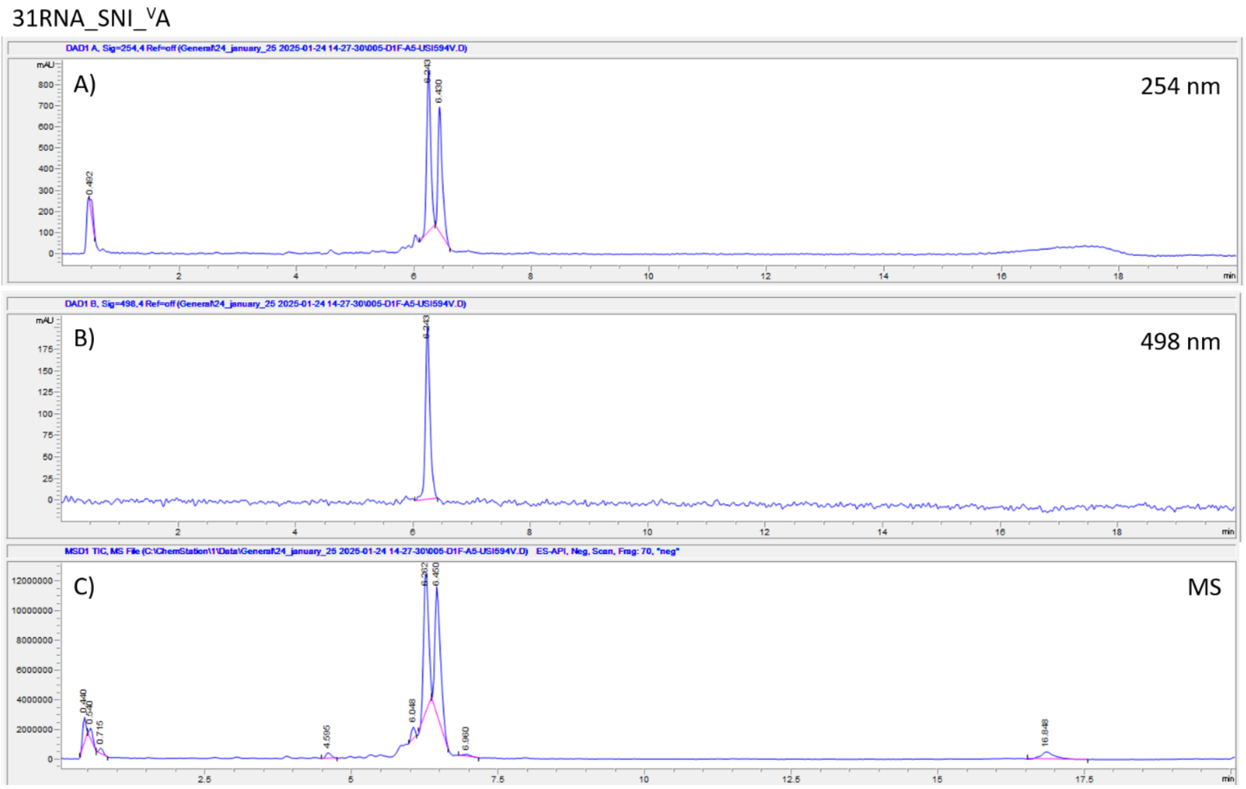

Figure S42. UPLC and MS chromatograms of **31RNA_SNI_^{V}A** after PEX reaction acquired at A) absorbance 254 nm; B) absorbance 498 nm (FAM); and C) MSD TIC.

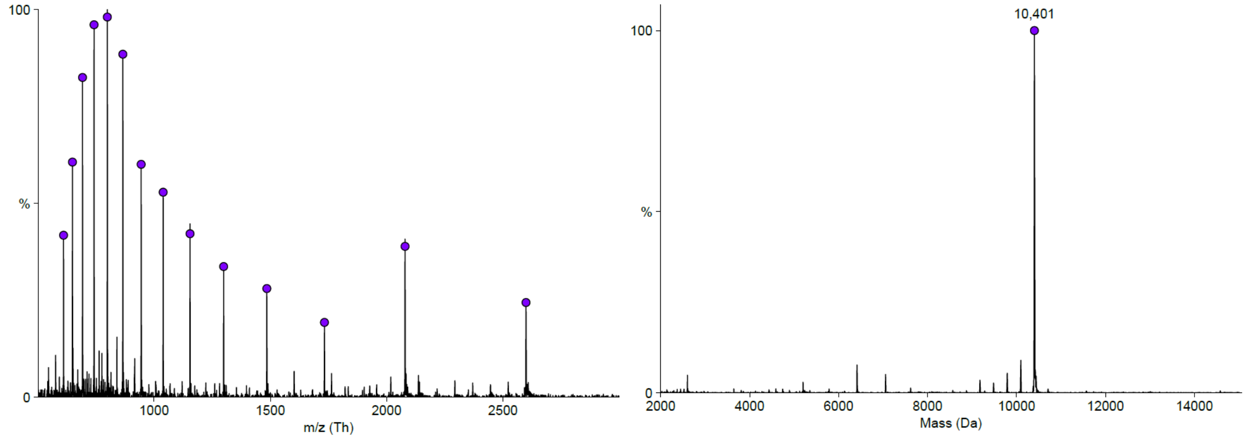

Figure S43. MS spectrum from UPLC-MS analysis of **31RNA_SNI_^{V}A** after PEX reaction. Left – raw spectrum, right - deconvoluted mass spectrum. Calculated mass: 10404 Da; found mass: 10401 Da.

31RNA_SNI_^{E}A

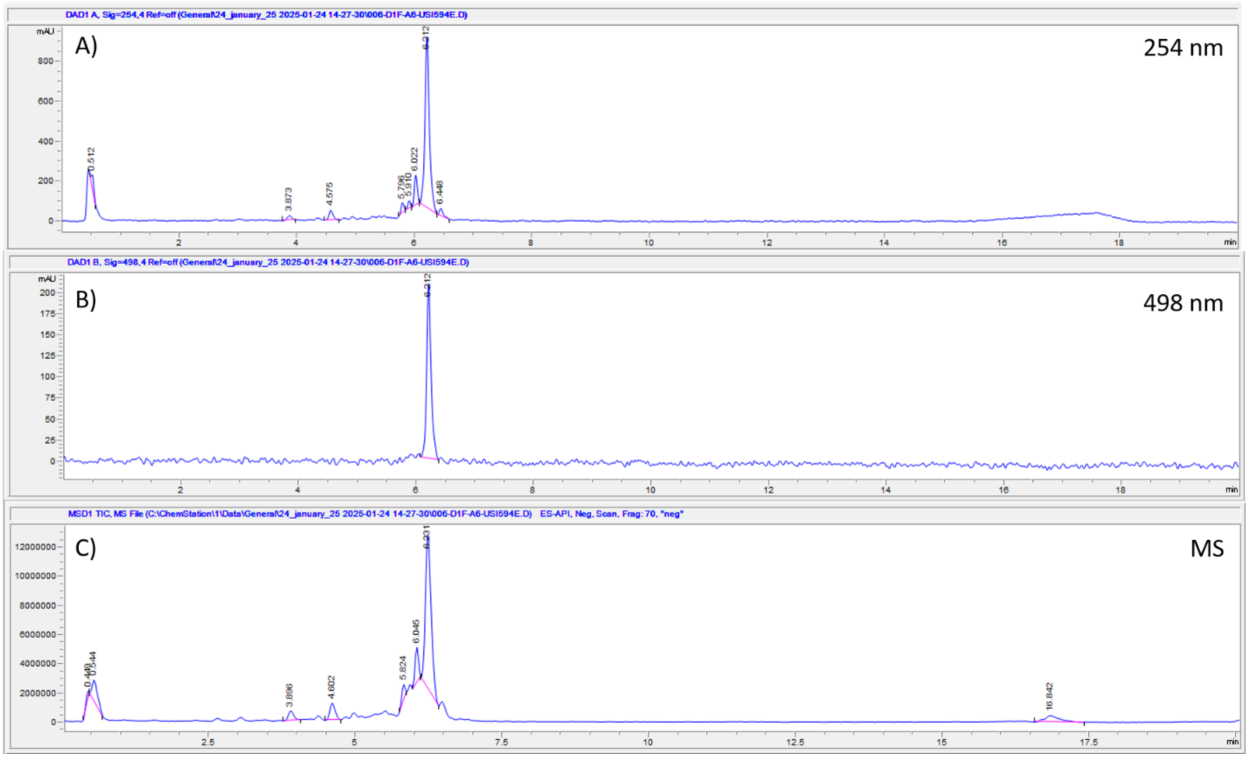

Figure S44. UPLC and MS chromatograms of **31RNA_SNI_^{E}A** after PEX reaction acquired at A) absorbance 254 nm; B) absorbance 498 nm (FAM); and C) MSD TIC.

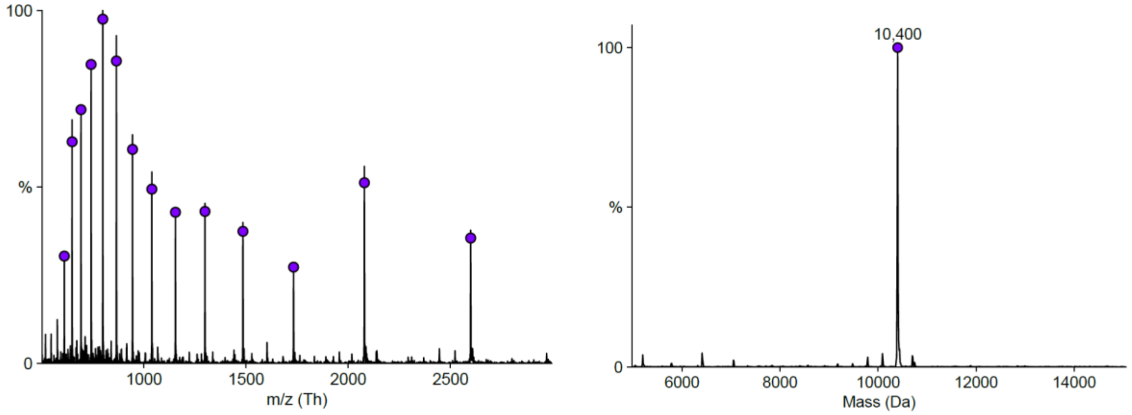

Figure S45. MS spectrum from UPLC-MS analysis of **31RNA_SNI_^{E}A** after PEX reaction. Left – raw spectrum, right - deconvoluted mass spectrum. Calculated mass: 10402 Da; found mass: 10400 Da.

31RNA_SNI_^{CM}A

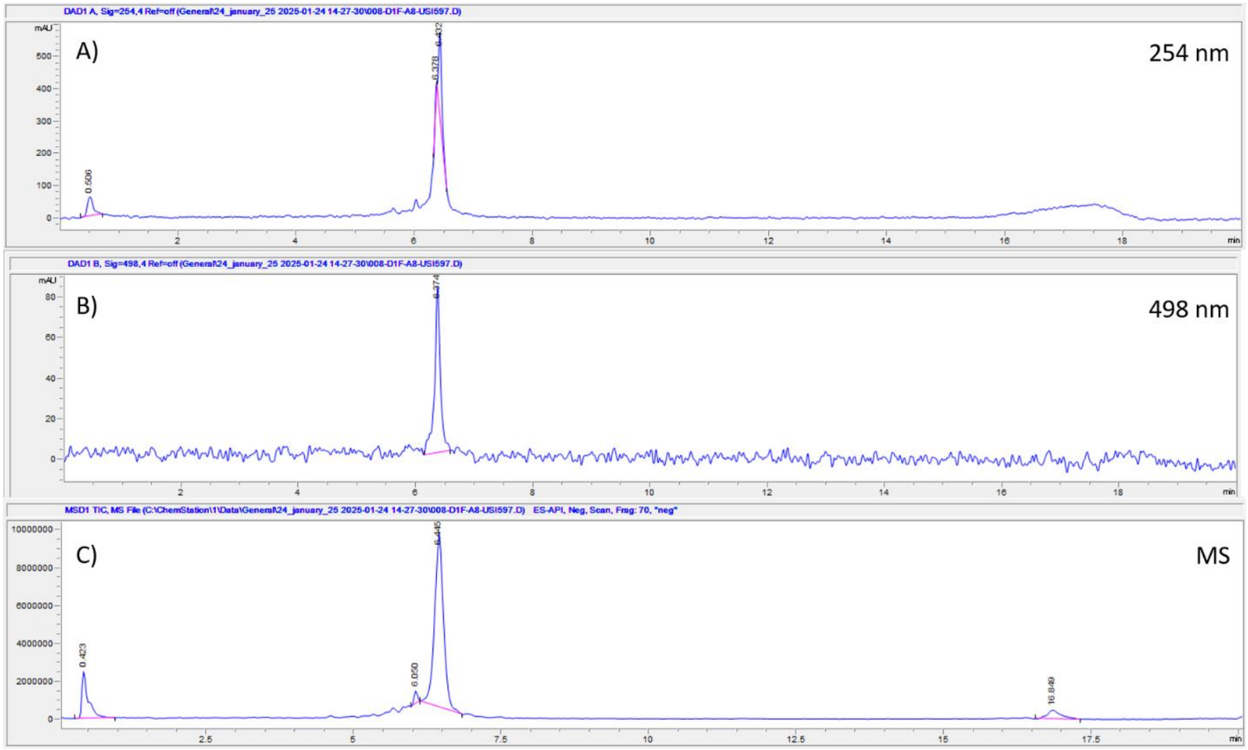

Figure S46. UPLC and MS chromatograms of **31RNA_SNI_ ^{CM}A** after PEX reaction acquired at A) absorbance 254 nm; B) absorbance 498 nm (FAM); and C) MSD TIC.

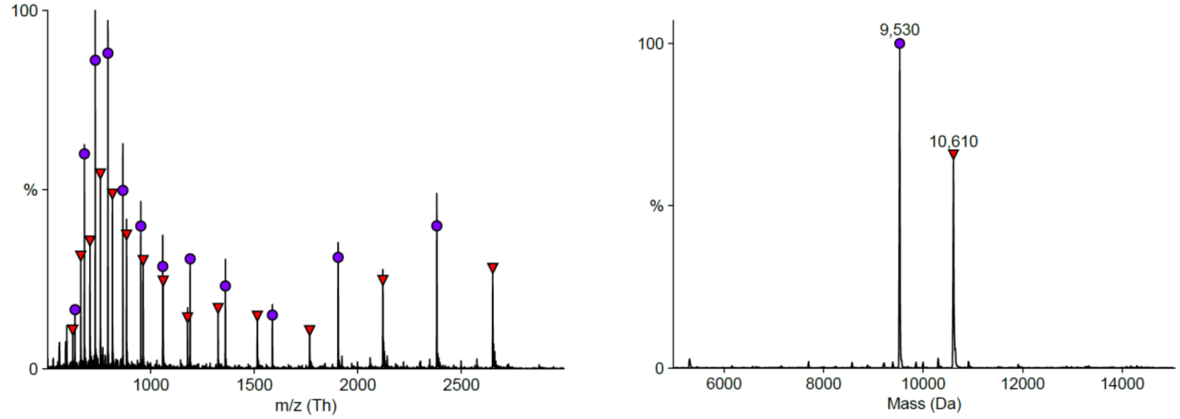

Figure S47. MS spectrum from UPLC-MS analysis of **31RNA_SNI_ ^{CM}A** after PEX reaction. Left – raw spectrum, right - deconvoluted mass spectrum. Calculated mass: 10612 Da; found mass: 10610 Da and 9530 Da (unknow, comes from starting **31RNA_SNI_ ^{V}A**).

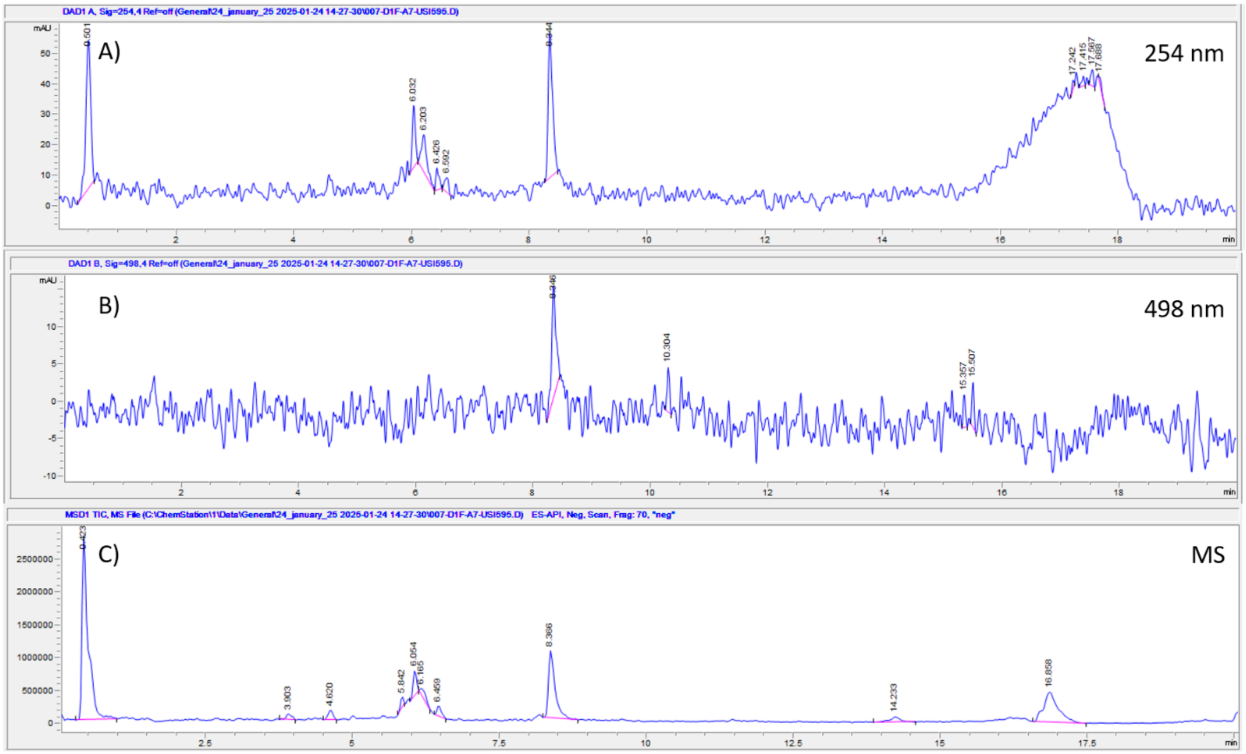

Figure S48. UPLC and MS chromatograms of **31RNA_SNI_ ^{Cy3}A** after PEX reaction acquired at A) absorbance 254 nm; B) absorbance 498 nm (FAM); and C) MSD TIC.

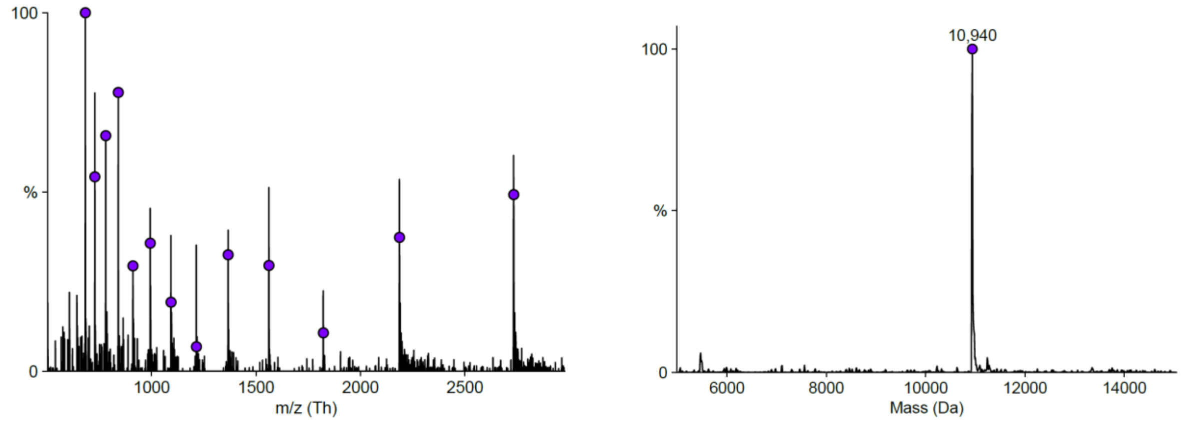

Figure S49. MS spectrum from UPLC-MS analysis of **31RNA_SNI_ ^{Cy3}A** after PEX reaction. Left – raw spectrum, right - deconvoluted mass spectrum. Calculated mass: 10941 Da; found mass: 10940 Da.

31RNA_SNI_A(+)_2M DNA polymerase

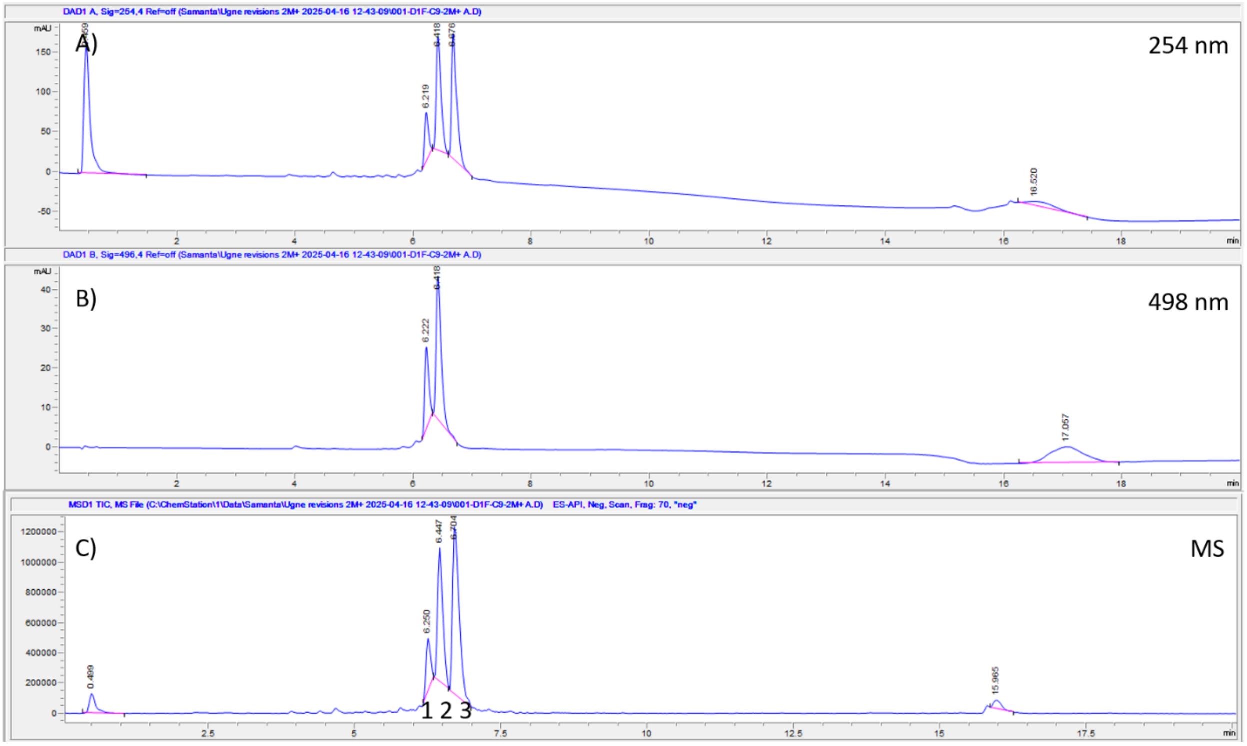

Figure S50. UPLC and MS chromatograms of **31NA_SNI_A(+)** after PEX reaction with 2M DNA polymerase acquired at A) absorbance 254 nm; B) absorbance 498 nm (FAM); and C) MSD TIC.

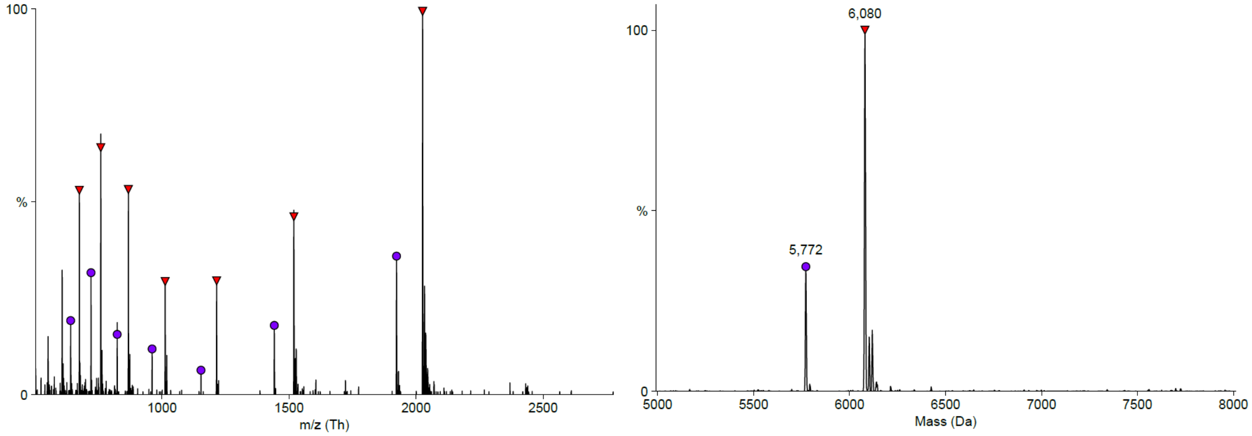

Figure S51. MS spectrum from UPLC-MS analysis of **31NA_SNI_A(+)_peak1** after PEX reaction with 2M DNA polymerase. Left – raw spectrum, right - deconvoluted mass spectrum. Calculated mass: 6075 Da; found mass: 6080 Da – corresponds to rPrim[15] + 2x rAMP (= 16RNA + rAMP).

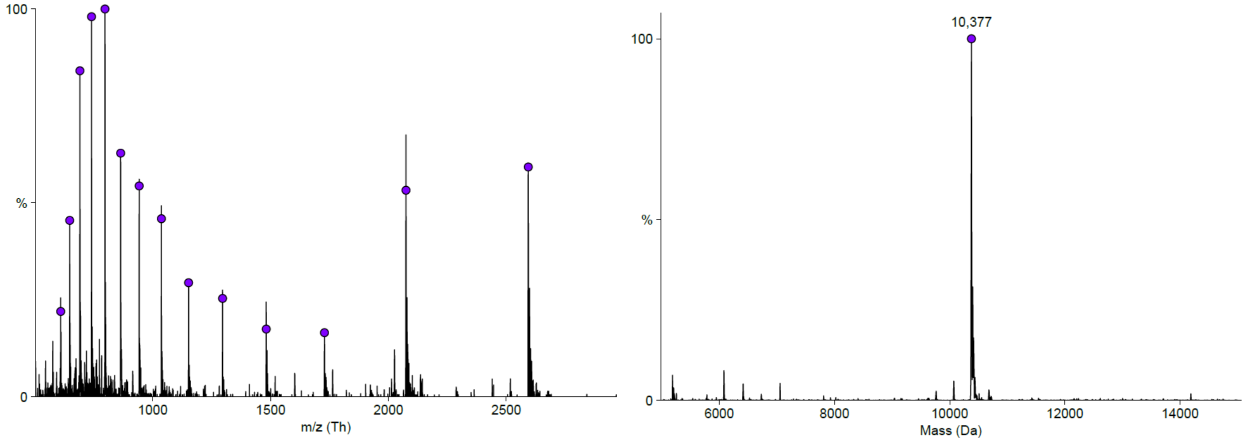

Figure S52. MS spectrum from UPLC-MS analysis of **31NA_SNI_A(+)_peak2** after PEX reaction with 2M DNA polymerase. Left – raw spectrum, right - deconvoluted mass spectrum. Calculated mass: 10375 Da; found mass: 10377 Da corresponding to full-length product **31RNA_SNI_A(+)**.

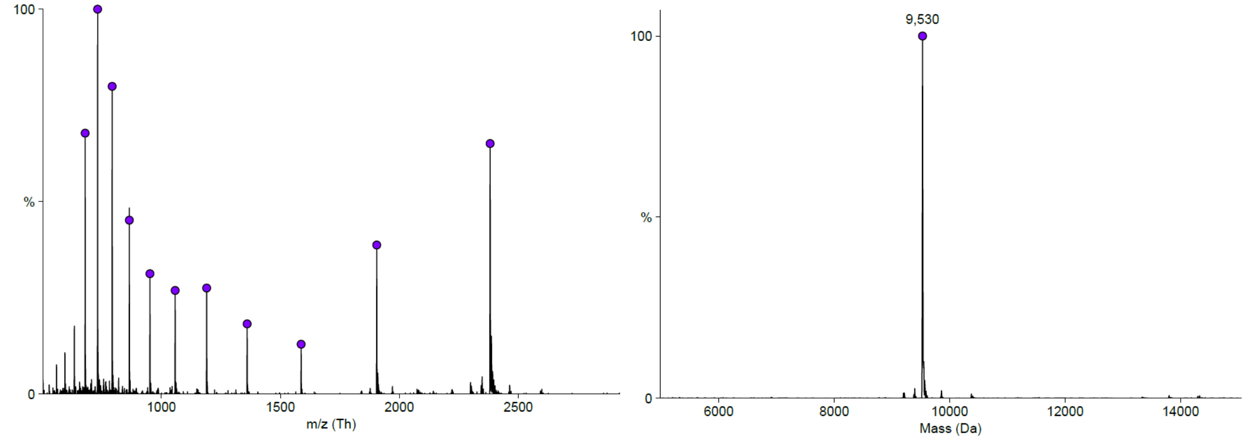

Figure S53. MS spectrum from UPLC-MS analysis of **31NA_SNI_A(+)_peak3** after PEX reaction with 2M DNA polymerase. Left – raw spectrum, right - deconvoluted mass spectrum. Calculated mass: 9532 Da; found mass: 9530 Da – corresponds to the template Temp^{31_SNI}.

31RNA_SNI_A(-)_2M DNA polymerase

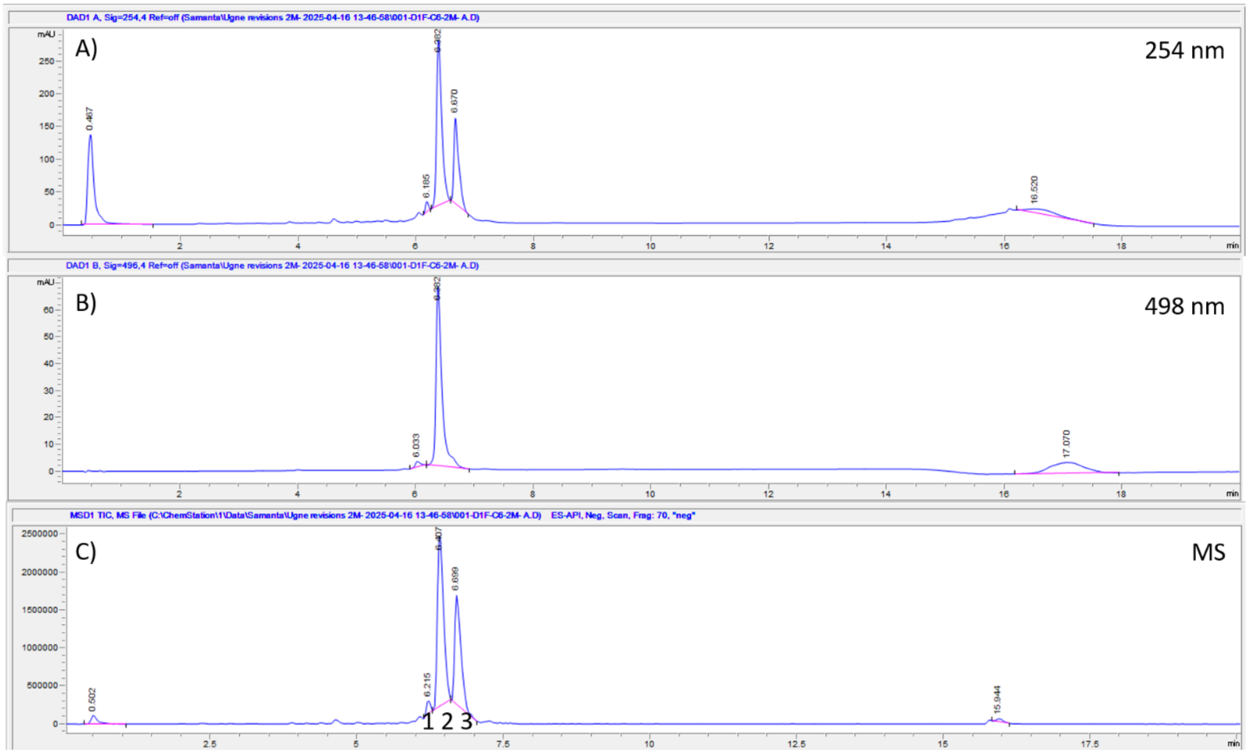

Figure S54. UPLC and MS chromatograms of **31NA_SNI_A(-)** after PEX reaction with 2M DNA polymerase acquired at A) absorbance 254 nm; B) absorbance 498 nm (FAM); and C) MSD TIC.

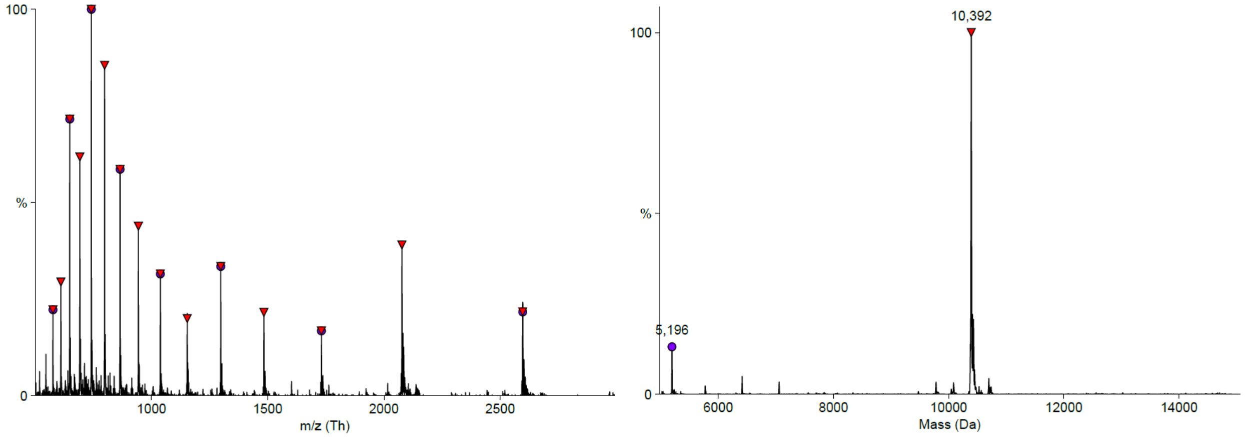

Figure S55. MS spectrum from UPLC-MS analysis of **31NA_SNI_A(-)_peak2** after PEX reaction with 2M DNA polymerase. Left – raw spectrum, right - deconvoluted mass spectrum. Calculated mass: 10395 Da; found mass: 10392 Da – corresponds to **31RNA_SNI_ A(+)** possessing misincorporation by rGMP instead of rAMP.

Note: Peak1 corresponds to $rPrim^{15}$ + 2x rAMP (= 16RNA + rAMP) as in Figure S51

Peak3 corresponds to the template $Temp^{31\_SNI}$ as in Figure S53.

31RNA_SNI_A(+)_TGK DNA polymerase

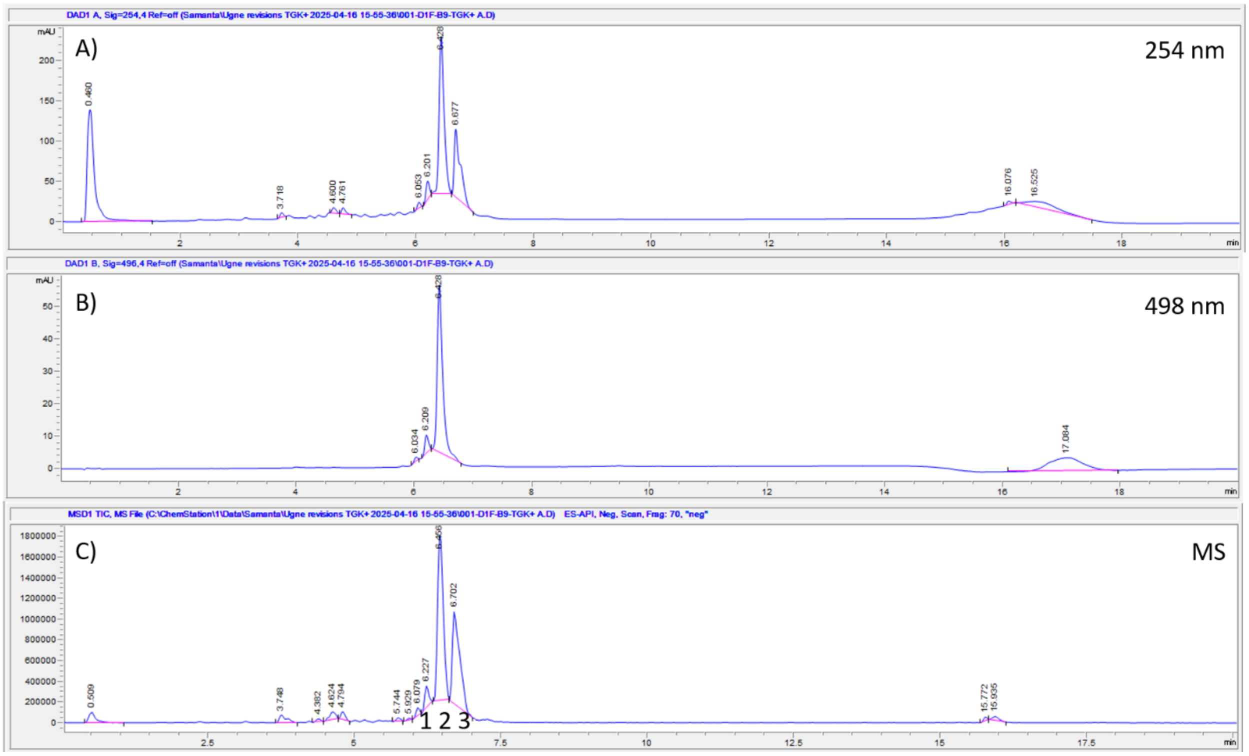

Figure S56. UPLC and MS chromatograms of **31NA_SNI_A(+)** after PEX reaction with TGK DNA polymerase acquired at A) absorbance 254 nm; B) absorbance 498 nm (FAM); and C) MSD TIC.

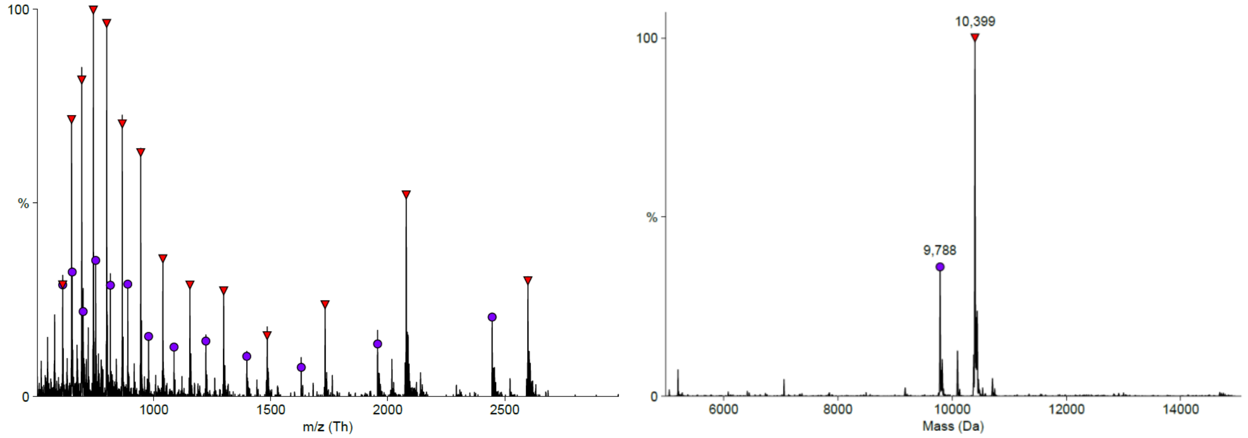

Figure S57. MS spectrum from UPLC-MS analysis of **31NA_SNI_A(+)_peak2** after PEX reaction with TGK DNA polymerase. Left – raw spectrum, right - deconvoluted mass spectrum. Calculated mass: 10375 Da; found mass: 10399 Da – corresponds to Na^+ form of full-length product **31RNA_SNI_ A(+)**, and found mass: 9788 Da – corresponds to Na^+ form of **31RNA_SNI_A(+)** truncated by rCMP and rUMP.

Note: Peak1 corresponds to $rPrim^{15}$ + 2x rAMP (= 16RNA + rAMP) as in Figure S51

Peak3 corresponds to the template $Temp^{31\_SNI}$ as in Figure S53.

31RNA_SNI_A(-)_TGK DNA polymerase

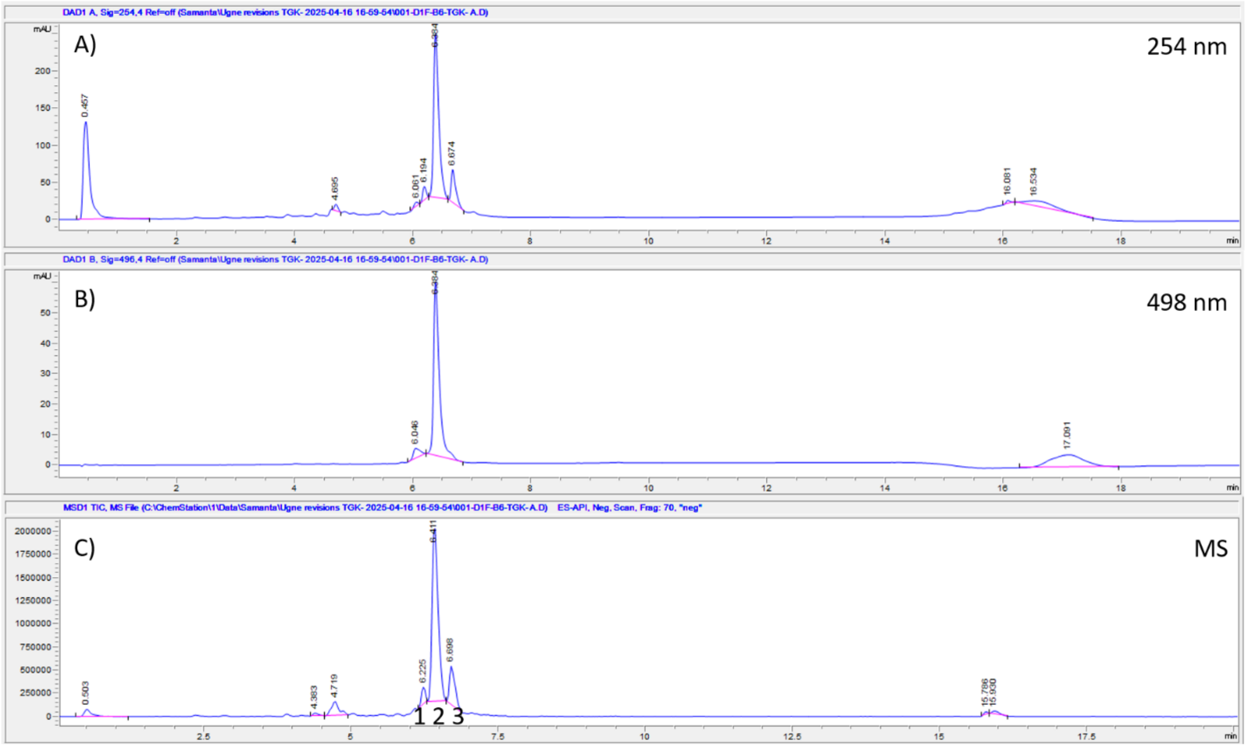

Figure S58. UPLC and MS chromatograms of **31NA_SNI_A(-)** after PEX reaction with TGK DNA polymerase acquired at A) absorbance 254 nm; B) absorbance 498 nm (FAM); and C) MSD TIC.

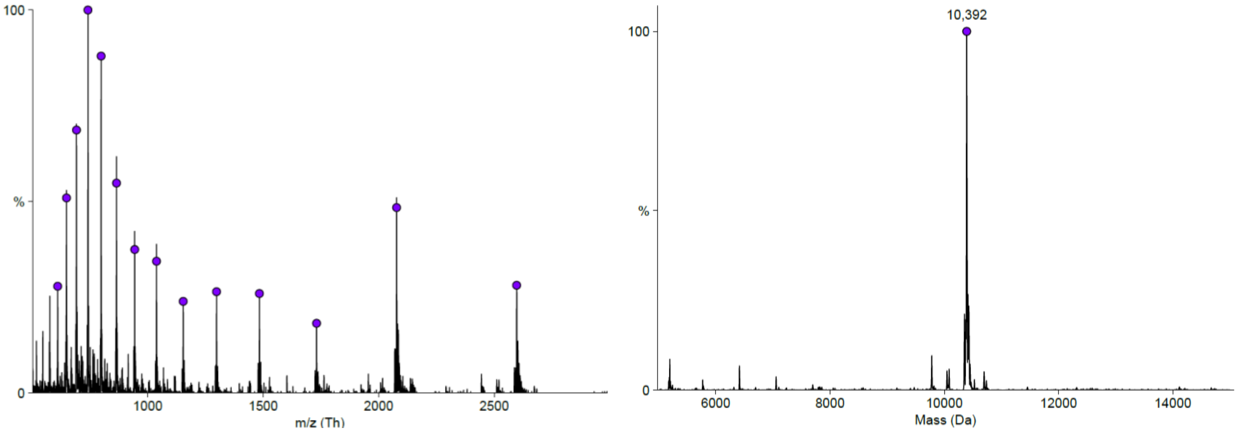

Figure S59. MS spectrum from UPLC-MS analysis of **31NA_SNI_A(-)_peak2** after PEX reaction with TGK DNA polymerase. Left – raw spectrum, right - deconvoluted mass spectrum. Calculated mass: 10395 Da; found mass: 10392 Da – corresponds to **31RNA_SNI_A(+)** possessing misincorporation by rGMP instead of rAMP.

Note: Peak1 corresponds to rPrim^{15} + 2x rAMP (= 16RNA + rAMP) as in Figure S51

Peak3 corresponds to the template Temp^{31_SNI} as in Figure S53.

6. Analysis of positive and negative controls in SNI experiments

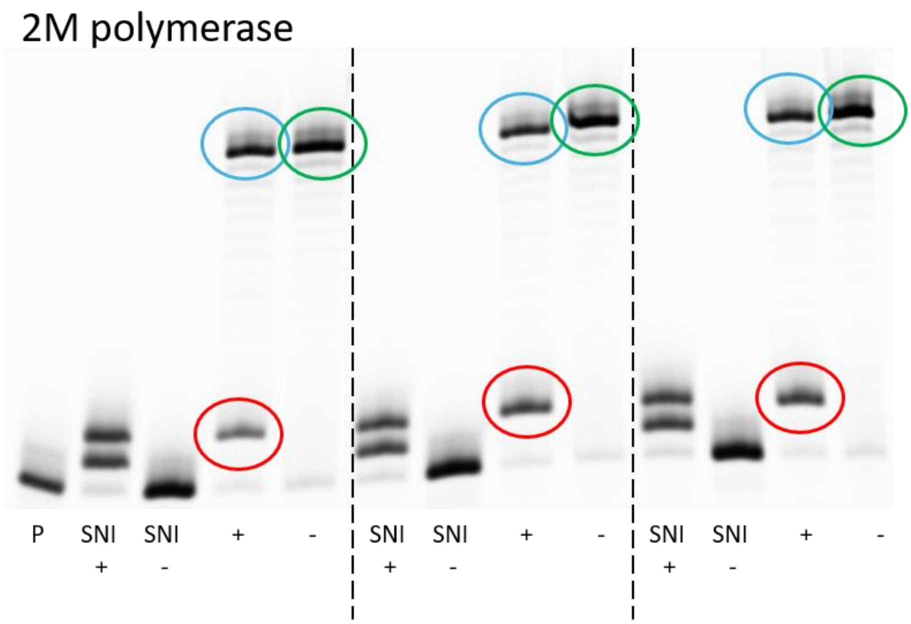

Figure S60. 15% dPAGE of 5'-(6-FAM)-labelled **16NA_A** and **31NA_A** (DNA·RNA hybrid) after SNI and PEX reactions with 2M DNA polymerase done in triplicate. (**P**) primer, (SNI+) positive control (natural rATP), (SNI**-**) negative control (water), (+) positive control (PEX after SNI+ with addition of rGTP, rCTP, rUTP), (-) negative control (PEX after SNI- with addition of rGTP, rCTP, rUTP).

Red circle: by-product corresponding to rPrim^{15} + 2x rAMP (= 16RNA + rAMP)

Blue circle: proper full-length product **31NA_SNI_ A(+)**

Green circle: corresponding to **31NA_SNI_ A(+)** possessing misincorporation by rGMP into the complementar position of TTP in the template. For more details (UPLC-MS) see Figures S50 – S55.

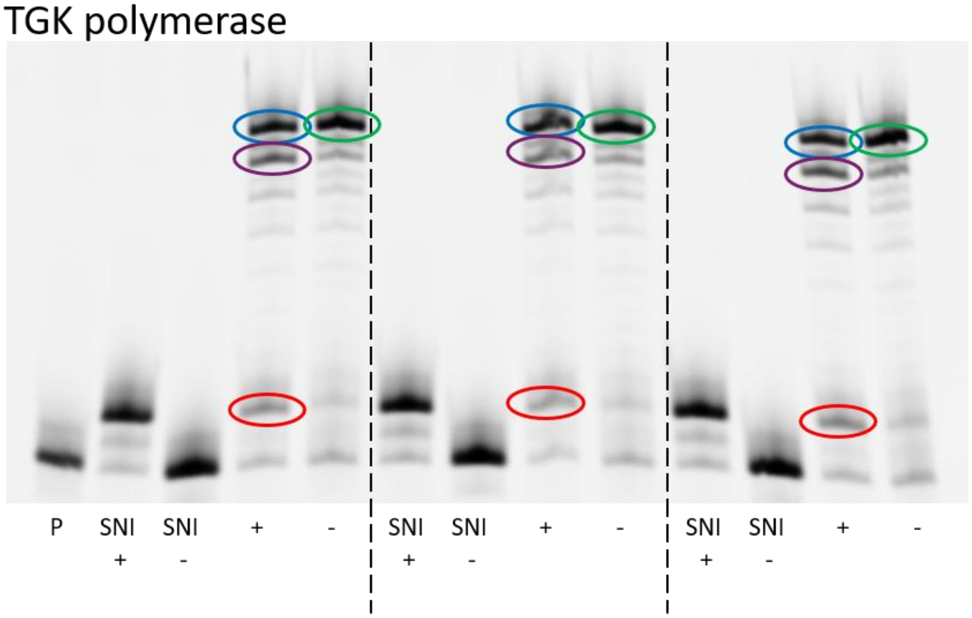

Figure S61. 15% dPAGE of 5'-(6-FAM)-labelled **16NA_A** and **31NA_A** (DNA·RNA hybrid) after SNI and PEX reactions with TGK DNA polymerase done in triplicate. (**P**) primer, (SNI+) positive control (natural rATP), (SNI-) negative control (water), (+) positive control (PEX after SNI+ with addition of rGTP, rCTP, rUTP), (-) negative control (PEX after SNI- with addition of rGTP, rCTP, rUTP).
Red circle: by-product corresponding to rPrim^{15} + 2x rAMP (= 16RNA + rAMP)
Blue circle: proper full-length product **31NA_SNI_ A(+)**
Violet circle: by-product of **31NA_SNI_ A(+)** truncated by rCMP and rUMP
Green circle: corresponding to **31NA_SNI_ A(+)** possessing misincorporation by rGMP into the complementar position of TTP in the template. For more details (UPLC-MS) see Figures S56 – S59.

Table S4. Mass after UPLC-MS analysis

Entry	RNAs after PEX	Mw [Da] calculated	Mw [Da] observed	Δ [Da]	Figures
1	**31RNA_SNI_A(+)_peak2**[a]	10375	10377	2	S50-S53
2	**31RNA_SNI_A(-)_ peak2**[a,b]	10395	10392	3	S54-S55
3	**31RNA_SNI_A(+)_peak2**[c]	10375	10399[d]	24	S56, S7
4	**31RNA_SNI_A(-)_peak2**[c]	10395	10392	3	S58, S59

a – for 2M DNA polymerase, b – product possessing misincorporation by rGMP instead of rAMP, c – for TGK DNA polymerase, d – product + Na^{+}.

7. References

1. J. Matyašovský, P. Perlíková, V. Malnuit, R. Pohl, M. Hocek, *Angew. Chem. Int. Ed.* **2016**, *55*, 15856.

2. F. Yan, J. M. LaMarre, R. Röhrich, J. Wiesner, H. Jomaa, A. S. Mankin, and D. Galonić Fujimori, *J. Am. Chem. Soc.* **2010**, *132*, 3953-3964.

3. T. Kovács, L. Ötvös, *Tetrahedron Lett.* **1988**, *29*, 4525–4528.

4. M. Gupta, M. Singha, D. B. Rasale, Z. Zhou, S. Bhandari, S. Beasley, J. Sakr, S. M. Parker, and R. C. Spitale, *Org. Lett.* **2021**, *23*, 7183-7187.

5. Y. Wang, D. Rösner, M. Grzywa and A. Marx, *Angew. Chem. Int. Ed.*, **2014**, *53*, 8159-8162.

6. C. Creech, M. Kanaujia and C. P. Causey, *Org. Biomol. Chem.*, **2015**, *13*, 8550-8555.

7. E. W. van Tilburg, M. Gremmen, J. von Frijtag Drabbe Künzel, M. de Groote, A. P IJzerman, *Bioorg. Med. Chem.*, **2003**, *11*, 2183-2192.

8. S. Becker, C. Schneider, H. Okamura, A. Crisp, T. Amatov, M. Dejmek and T. Carell, *Nat. Commun.*, **2018**, *9*, 163.

9. C. Cozens, V. B. Pinheiro, A. Vaisman, R. Woodgate and P. A. Holliger, *Proc. Natl. Acad. Sci. U. S. A*, **2012**, *109*, 8067–8072.

10. M. R. Dunn, C. Otto, K. E. Fenton and J. C. Chaput, *ACS Chem. Biol*, **2016**, *11*, 1210–1219.

11. T. Chen, N. Hongdilokkul, Z. Liu, R. Adhikary, S. S. Tsuen and F. E. Romesberg, *Nat. Chem.*, **2016**, *8*, 556-562.

12. N. Freund, A. I. Taylor, S. Arangundy-Franklin, N. Subramanian, S.-Y. Peak-Chew, A. M. Whitaker, B. D. Freudenthal, M. Abramov, P. Herdewijn and P. Holliger, *Nat. Chem.*, **2023**, *15*, 91-100.

13. S. Akita, N. Umezawa, T. Higuchi, *Org. Lett.* **2005**, *7*, 5565-5568.

14. M. T. Marty, A. J. Baldwin, E. G. Marklund, G. K. A. Hochber, J. L. P. Benesch and C. V. Robinson, *Anal. Chem.*, **2015**, *87*, 4370-4376.

15. R. M. Mitton-Fry, J. Eschenbach, H. Schepers, R. Rasche, M. Erguven, D. Kümmel, A. Rentmeister and N. V. Cornelissen, *Chem. Sci.*, **2024**, *15*, 13068-13073.
